# Radical and Cationic Pathways in C(*sp*^3^)–H Bond Oxygenation by Dioxiranes of Bicyclic and Spirocyclic Hydrocarbons Bearing Cyclopropane Moieties

**DOI:** 10.1021/jacs.3c07163

**Published:** 2023-10-24

**Authors:** Marco Galeotti, Woojin Lee, Sergio Sisti, Martina Casciotti, Michela Salamone, K. N. Houk, Massimo Bietti

**Affiliations:** † Dipartimento di Scienze e Tecnologie Chimiche, Università “Tor Vergata”, Via della Ricerca Scientifica 1, I-00133, Rome, Italy; ‡ QBIS Research Group, Institut de Química Computacional i Catàlisi (IQCC) and Departament de Química, Universitat de Girona, Campus Montilivi, Girona E-17071, Catalonia, Spain; § Department of Chemistry and Biochemistry, University of California, Los Angeles, California 90095, United States

## Abstract

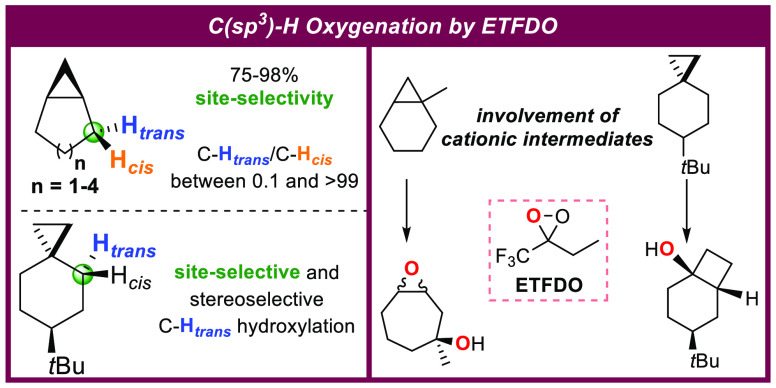

A product and DFT computational study on the reactions of 3-ethyl-3-(trifluoromethyl)dioxirane (ETFDO) with bicyclic and spirocyclic hydrocarbons bearing cyclopropyl groups was carried out. With bicyclo[*n*.1.0]alkanes (*n* = 3–6), diastereoselective formation of the alcohol product derived from C_2_–H bond hydroxylation was observed, accompanied by smaller amounts of products derived from oxygenation at other sites. With 1-methylbicyclo[4.1.0]heptane, rearranged products were also observed in addition to the unrearranged products deriving from oxygenation at the most activated C_2_–H and C_5_–H bonds. With spiro[2.5]octane and 6-*tert*-butylspiro[2.5]octane, reaction with ETFDO occurred predominantly or exclusively at the axial C_4_–H to give unrearranged oxygenation products, accompanied by smaller amounts of rearranged bicyclo[4.2.0]octan-1-ols. The good to outstanding site-selectivities and diastereoselectivities are paralleled by the calculated activation free energies for the corresponding reaction pathways. Computations show that the σ* orbitals of the bicyclo[*n*.1.0]alkane *cis* or *trans* C_2_–H bonds and spiro[2.5]octanes axial C_4_–H bond hyperconjugatively interact with the Walsh orbitals of the cyclopropane ring, activating these bonds toward HAT to ETFDO. The detection of rearranged oxygenation products in the oxidation of 1-methylbicyclo[4.1.0]heptane, spiro[2.5]octane, and 6-*tert*-butylspiro[2.5]octane provides unambiguous evidence for the involvement of cationic intermediates in these reactions, representing the first examples on the operation of ET pathways in dioxirane-mediated C(*sp*^3^)–H bond oxygenations. Computations support these findings, showing that formation of cationic intermediates is associated with specific stabilizing hyperconjugative interactions between the incipient carbon radical and the cyclopropane C–C bonding orbitals that trigger ET to the incipient dioxirane derived 1,1,1-trifluoro-2-hydroxy-2-butoxyl radical.

## Introduction

The cyclopropyl group is an important and versatile motif. Because of its characteristic structural and bonding features,^1^ substitution of cyclopropane can modify the properties of substrates and provide access to a variety of useful synthetic transformations. Accordingly, cyclopropane-containing molecules are finding increasing application in organic synthesis,^2^ in drug development,^3^ and as functional molecules in different fields.^4^ The cyclopropyl group is also present in several natural products including terpenoids, steroids, and alkaloids, among which, many show biological activity and may serve as potential drug leads.^5^

**Figure 1:**
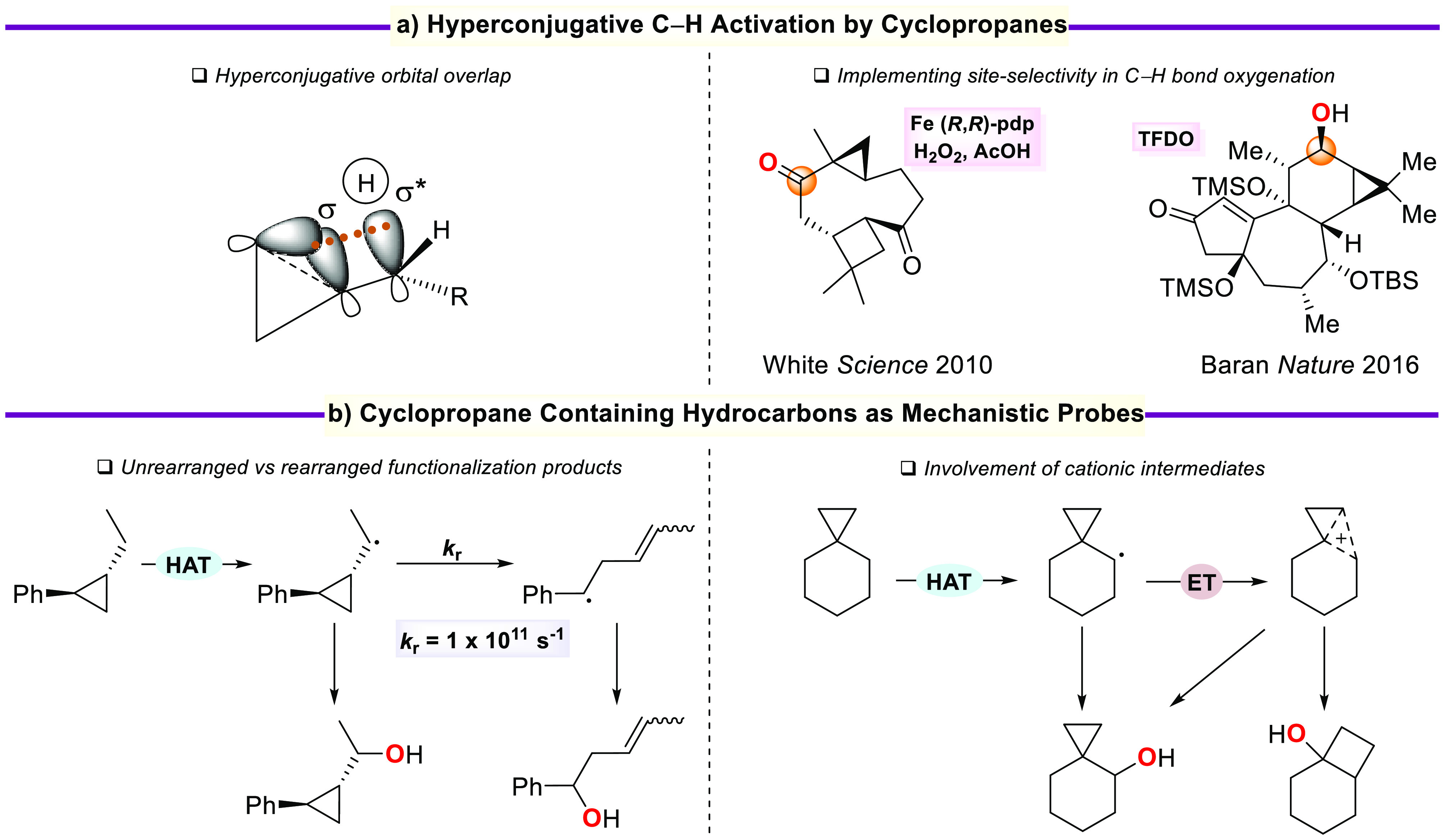
Use of cyclopropyl containing substrates (a) to induce selectivity in HAT-based C–H bond functionalization procedures and (b) as mechanistic probes.

A promising approach for structural diversification of cyclopropane containing molecules is represented by C(*sp*^3^)–H bond functionalization, a mainstream topic of modern synthetic chemistry.^6^ Overlap between a cyclopropane Walsh C–C bonding orbital and the σ* antibonding orbital of an α-C–H activates this bond toward functionalization (Figure 1a), providing a powerful handle to implement site-selectivity in these reactions.^6a^

Concerted insertion or two-step hydrogen atom transfer (HAT) strategies typically occur. In the latter case, however, because the intermediate cyclopropylcarbinyl radicals formed in the HAT step are known to undergo rapid rearrangement,^7^ the procedure is limited to the use of reagents that ensure very fast radical capture, preventing competitive unimolecular pathways and delivering the unrearranged functionalized product. Metal-oxo species,^8^ dioxiranes,^9^ and oxaziridines^10^ are examples of such reagents, able to promote stereoretentive C(*sp*^3^)–H oxygenations.

Along these lines, the C–H bond oxygenation of linear, bicyclic, and spirocyclic substrates bearing cyclopropane moieties has been studied employing a variety of oxygenation reagents.^11^ High selectivity for hydroxylation and ketonization at the activated α-methylenes over other sites has been generally observed. Similar selectivity patterns have been observed in dihalocarbene insertions into the C(*sp*^3^)–H bonds of hydrocarbons bearing cyclopropane moieties.^12^

In the framework of synthetically useful procedures, the full potential of this activation is witnessed by the results obtained by White in the site-selective C–H bond ketonization of a terpenoid derivative with H_2_O_2_ catalyzed by the Fe (*R*,*R*)-pdp complex,^11f^ and by Baran in the site-selective and stereoselective C–H bond hydroxylation promoted by 3-methyl-3-(trifluoromethyl)dioxirane (TFDO), employed in an intermediate step of the total synthesis of (+)-phorbol (Figure 1a).^13^

Because of the tendency of cyclopropylcarbinyl radicals to undergo rapid rearrangement,^7^ cyclopropane-containing substrates are coveted mechanistic probes to study the involvement of radical intermediates in a reaction,^14^ to assess the concerted, radical, and/or cationic nature of enzymatic and biomimetic reaction mechanisms,^8a,15^ as well as to calibrate the rates of competing radical reactions (Figure 1b). For example, *trans*-1-ethyl-2-phenylcyclopropane has been employed as a probe to calibrate the rate constant for recombination of the radical couple formed in the first step of its reaction with dimethyldioxirane (DMDO).^16^ Based on a ring-opening rate constant *k*_r_ = 1 × 10^11^ s^–1^, and a 40:1 unrearranged/rearranged product ratio, a rate constant *k* = 4 × 10^12^ s^–1^ could be estimated at room temperature corresponding to a lifetime of the radical couple of 200 fs.

With spiro[2.5]octane, the corresponding cyclopropylcarbinyl radical undergoes ring-opening with *k*_r_ = 5 × 10^7^ s^–1^.^15a^ In the framework of the oxygenation of this substrate promoted by metal-oxo species,^11f^ dioxiranes,^11e^ ozone,^11a^ and cytochrome P450 enzymes,^15a^ no evidence for the formation of products deriving from radical rearrangement has been observed, in line with the relatively low value of *k*_r_ that prevents competition with the radical capture or radical recombination steps.

**Scheme 1:**
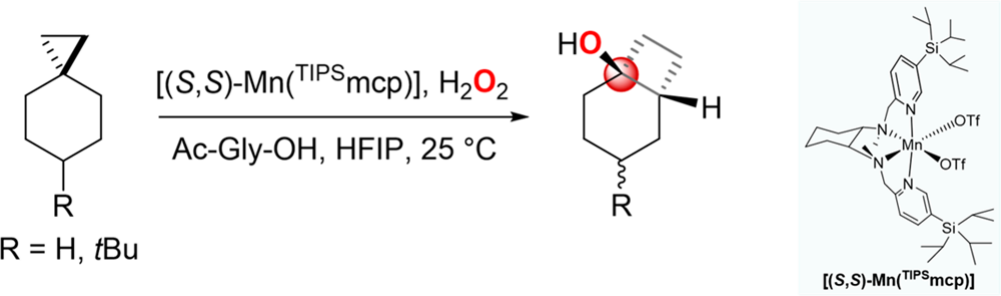
Results Obtained in the Oxidation of Spiro[2.5]octanes with H_2_O_2_ Catalyzed by [(*S,S*)-Mn(^TIPS^mcp)] (HFIP = 1,1,1,3,3,3-Hexafluoro-2-propanol)

With substrates such as spiro[2.5]octane and bicyclo[4.1.0]heptane (norcarane), the product distribution can also provide information on the possible involvement of cationic intermediates, revealing the occurrence of competitive ET steps.^15a^ In the specific case of spiro[2.5]octane, the formation of bicyclo[4.2.0]octan-1-ol can provide conclusive evidence for the involvement of a cationic intermediate. Evidence for the formation of rearranged alcohol products has been obtained in a recent study on the oxygenation of spiro[2.5]octane and 6-*tert*-butylspiro[2.5]octane promoted by manganese-oxo species, where leveraging on the use of fluorinated alcohol solvents and on catalyst electronics, predominant or exclusive formation of bicyclo[4.2.0]octan-1-ol and *cis*-4-(*tert*-butyl)-bicyclo[4.2.0]octan-1-ol, respectively, was observed (Scheme 1).^17^

**Scheme 2:**
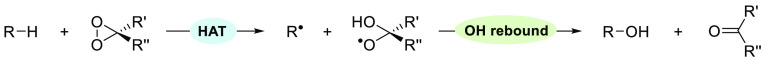
Mechanism of C(*sp*^3^)–H Bond Oxidation by Dioxiranes

Because similar mechanistic features are associated with oxygenations promoted by metal-oxo species and dioxiranes,^8,18^ and considering that the oxidizing ability of the intermediate α-hydroxy alkoxyl radical formed following HAT to the dioxirane (Scheme 2) can be modulated by careful choice of the precursor ketone as well as by solvent effects, we explored if these reagents in combination with fluorinated alcohol solvents could lead to the (unprecedented) involvement of cationic intermediates in dioxirane reactions.

**Figure 2:**
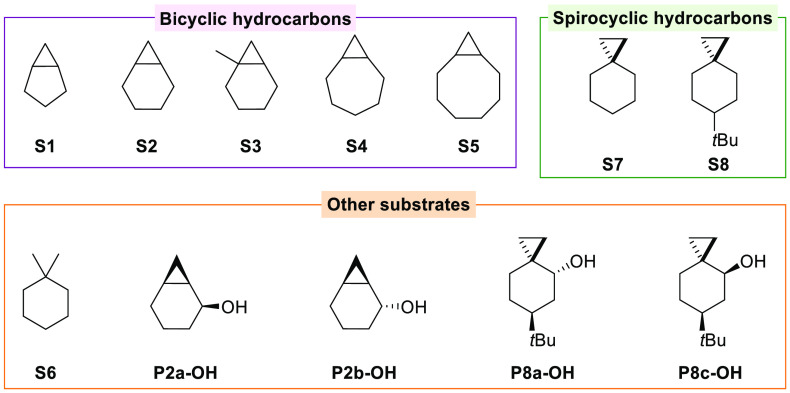
Structures of the substrates investigated in this work.

We report on the results of a detailed product and computational study of the reactions of 3-ethyl-3-(trifluoromethyl)dioxirane (ETFDO) with bicyclic (**S1–S5**) and spirocyclic (**S7** and **S8**) hydrocarbons bearing cyclopropyl groups, the structures for which are displayed in Figure 2. Product studies have been also extended to 1,1-dimethylcyclohexane (**S6**) and to the diastereomeric alcohol couples **P2a-OH**, **P2b-OH**, **P8a-OH**, and **P8c-OH** (Figure 2).

## Results

### Reactions with ETFDO

**Scheme 3:**
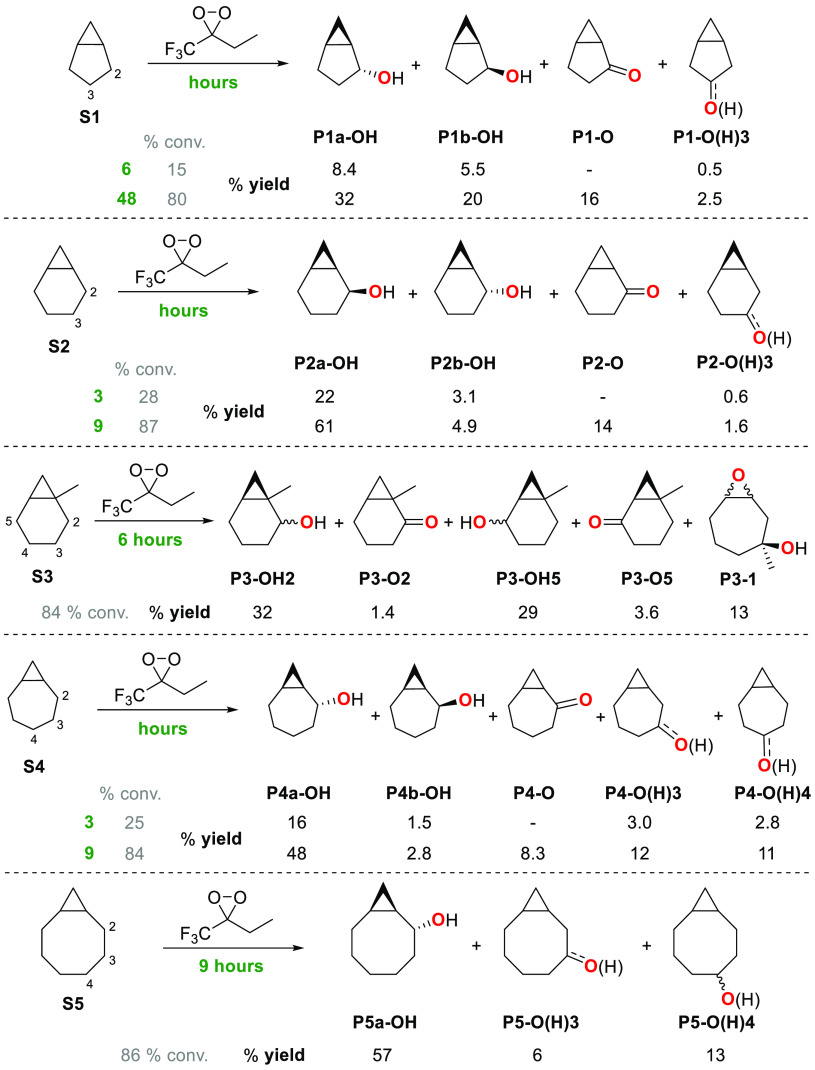
Oxygenation of Bicyclo[*n*.1.0]alkanes (*n* = 3–6) (**S1–S5**) Promoted by ETFDO

**Scheme 4:**
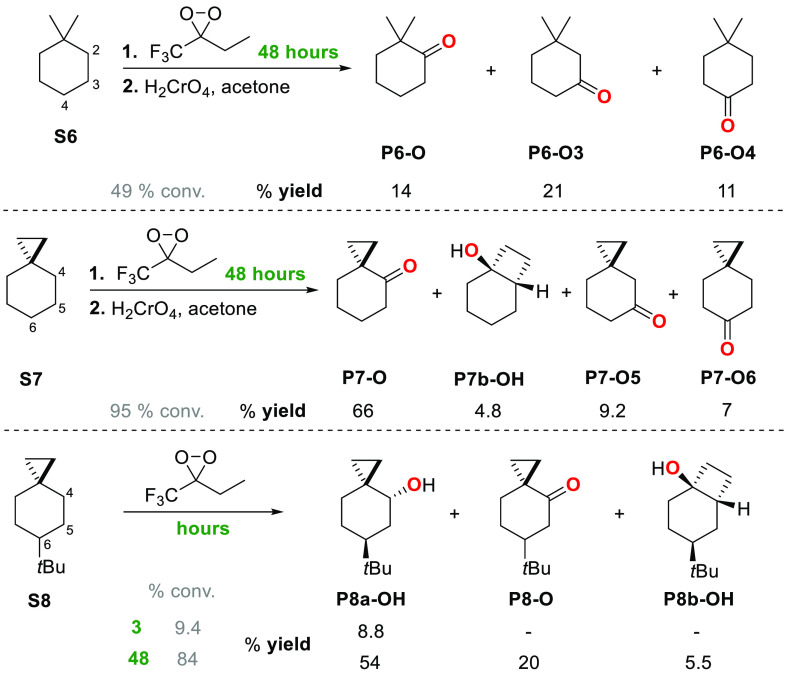
Oxygenation of 1,1-Dimethylcyclohexane (**S6**) and of Spiro[2.5]octanes (**S7** and **S8**) Promoted by ETFDO

The reactions of substrates **S1–S8** with *in situ* generated ETFDO were carried out at 0 °C in a 1,1,1,3,3,3-hexafluoro-2-propanol (HFIP)/H_2_O 3:1 solvent mixture containing the substrate (1 equiv), oxone (1 equiv), NaHCO_3_ (4 equiv), 1,1,1-trifluoro-2-butanone (0.2 equiv), and Bu_4_NHSO_4_ 0.05 equiv, according to a previously reported procedure.^19^ Product yields for the oxygenation of bicyclic hydrocarbons (**S1–S5**), 1,1-dimethylcyclohexane (**S6**), and spirocyclic hydrocarbons (**S7** and **S8**) by ETFDO are shown in Scheme 3 and Scheme 4. The schemes show the results obtained at ≥80% conversion, where the total yields of the oxygenation products approach 87%. This is accompanied by ≥90% mass balances. With **S6**, a 49% conversion was observed after a 48 h reaction time with a 46% total yield of oxygenation products (Scheme 4). Schemes 3 and 4 also present the product yields obtained at low conversion with substrates **S1**, **S2**, **S4**, and **S8**. The ketone products arising from overoxidation of the first formed alcohols at the C–H bonds that are α to the cyclopropyl group are not observed under low conversion conditions. Full experimental details are reported in the Supporting Information (SI) (Tables S1–S8).

The yield of the minor products deriving from C–H bond oxygenation at remote positions (C-3 for **S1** and **S2**; C-3 and C-4 for **S4** and **S5**) was calculated as the sum of the alcohol and ketone products. In the oxygenation of **S3**, product yields of alcohols at C-2 and C-5 are given in both cases as the sum of the *cis*- and *trans*- isomers (full details on the product distributions are displayed in the SI, Table S3). For the oxidation of **S6** and **S7**, product yields were obtained after chromic acid oxidation of the reaction mixture (see SI, Tables S6 and S7).

**Scheme 5:**
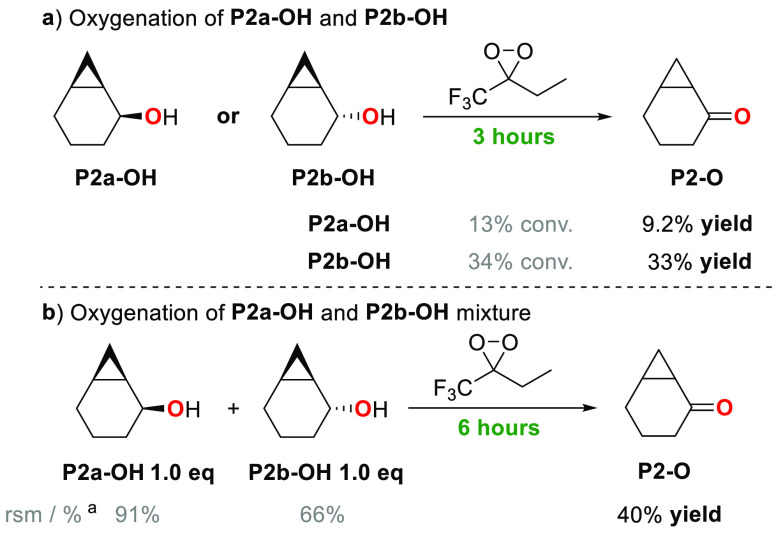
Oxygenation of *cis*-Bicyclo[4.1.0]heptan-2-ol (**P2a-OH**) and *trans*-Bicyclo[4.1.0]heptan-2-ol (**P2b-OH**) Conversion and product yields were determined by GC and averaged over two independent experiments. (a) Reaction conditions: **P2a-OH** or **P2b-OH** 1 equiv, oxone 1 equiv, NaHCO_3_ 4 equiv, 1,1,1-trifluoro-2-butanone 0.2 equiv, HFIP/H_2_O (3:1), Bu_4_NHSO_4_ 0.05 equiv, *T* = 0 °C, 3 h. (b) **P2a-OH** 1 equiv, **P2b-OH** 1 equiv, oxone 1 equiv, NaHCO_3_ 4 equiv, 1,1,1-trifluoro-2-butanone 0.2 equiv, HFIP/H_2_O (3:1), Bu_4_NHSO_4_ 0.05 equiv, *T* = 0 °C, 6 h. rsm: recovered starting material.

**Scheme 6:**
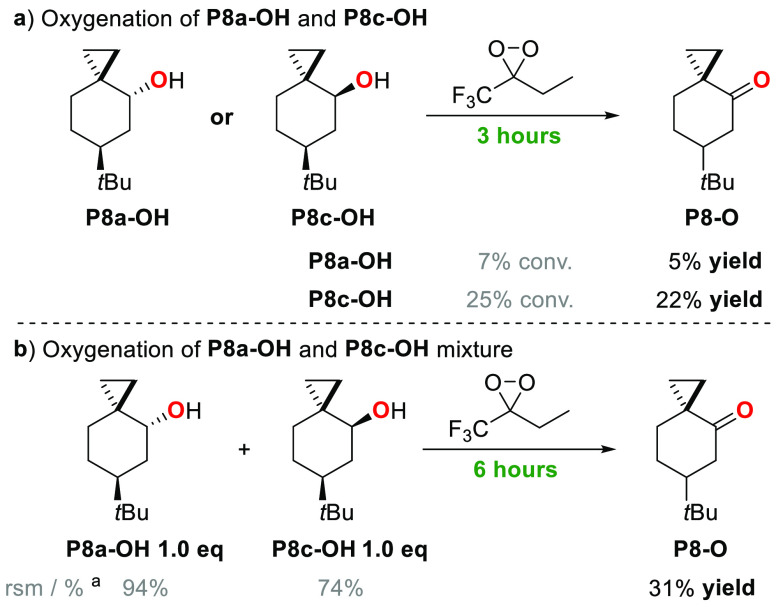
Oxygenation of *trans*-6-*tert*-Butylspiro[2.5]octan-2-ol (**P8a-OH**) and *cis*-6-*tert*-Butylspiro[2.5]octan-2-ol (**P8c-OH**) Conversion and product yields were determined by GC and averaged over two independent experiments. (a) Reaction conditions: **P8a-OH** or **P8c-OH** 1 equiv, oxone 1 equiv, NaHCO_3_ 4 equiv, 1,1,1-trifluoro-2-butanone 0.2 equiv, HFIP/H_2_O (3:1), Bu_4_NHSO_4_ 0.05 equiv, T = 0 °C, 3 h. (b) **P8a-OH** 1 equiv, **P8c-OH** 1 equiv, oxone 1 equiv, NaHCO_3_ 4 equiv, 1,1,1-trifluoro-2-butanone 0.2 equiv, HFIP/H_2_O (3.0:1.0), Bu_4_NHSO_4_ 0.05 equiv, T = 0 °C, 6 h. rsm: recovered starting material.

The reaction with ETFDO was also extended to some of the oxygenation products of **S2** and **S8**. The main reaction products **P2a-OH** and **P8a-OH** and the corresponding ketones **P2-O** and **P8-O** were isolated by the scale-up oxidation of **S2** and **S8**, respectively. **P2b-OH** and **P8c-OH** (the diastereoisomer of **P8a-OH**, not observed in the oxidation of **S8**) were prepared by diastereoselective reduction of parent ketones **P2-O** and **P8-O**, respectively (see SI). Conversions and product yields observed in the oxygenation of the isomeric *cis-* and *trans-* alcohol products **P2a-OH** and **P2b-OH** by ETFDO are displayed in Scheme 5a. The results of the competitive oxygenation of a 1:1 mixture of **P2a-OH** and **P2b-OH** by ETFDO are described in Scheme 5b. Scheme 6 shows the conversions and product yields that are observed in the corresponding experiments with **P8a-OH** and **P8c-OH**.

### Computational Studies

Density functional theory (DFT) computations were performed with Gaussian 16.^20^ The ωB97X-D functional was used to optimize molecular geometries,^21^ with the 6-311++G(d,p) basis set and the SMD solvation model accounting for H_2_O.^22^ Frequency calculations were conducted at the same level of theory used for the geometry optimizations to obtain thermal Gibbs free energies and characterize the stationary points on the potential energy surface. The correct unrestricted wave functions were obtained by performing a stability test with the Gaussian keyword *stable = opt*. Gibbs free energies were corrected using Goodvibes, which corrects the vibrational frequencies via the approximation for the quasi-harmonic correction, as proposed by Grimme.^23^ Intrinsic reaction coordinate (IRC) calculations were performed to verify that a transition state (TS) connects the reactant and product on the potential energy surface. CYLview was employed to visualize molecular structures.^24^

**Figure 3:**
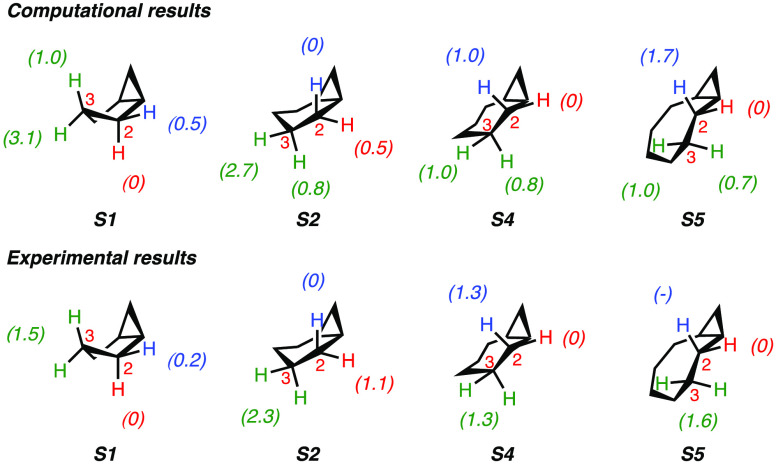
Difference in activation free energies (ΔΔ*G*^‡^, in kcal mol^–1^) for HAT from the C_2_–H and C_3_–H bonds in **S1**, **S2**, **S4**, and **S5** to ETFDO: computational and experimental studies.

The computed site-selectivities for C(*sp*^3^)–H bond oxygenation of bicyclo[n.1.0]alkanes **S1**, **S2**, **S4** and **S5** with ETFDO are shown in Figure 3. The relative activation free energies (ΔΔ*G*^‡^) for the C_2_–H and C_3_–H bonds are given in kcal mol^–1^. For comparison, the experimental ΔΔ*G*^‡^ values, which are derived from the experiments illustrated in Scheme 3 (for which the normalized site-selectivities are displayed in Figure 8), are also shown.

**Figure 4:**
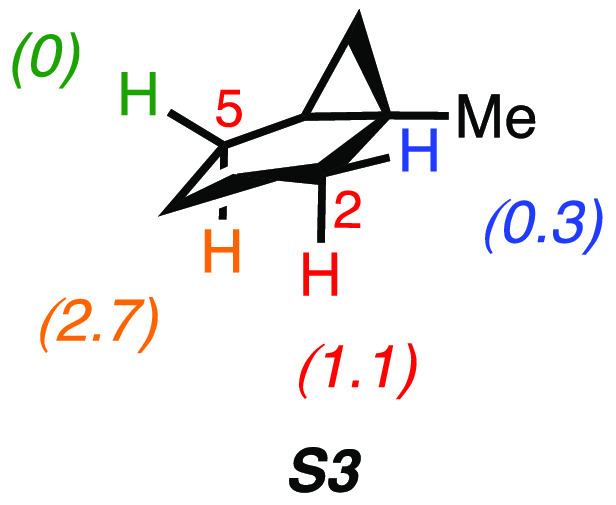
Computed difference in activation free energies (ΔΔ*G*^‡^, in kcal mol^–1^) for HAT from the C_2_–H and C_5_–H bonds in **S3** to ETFDO.

The pertinent transition structures obtained for these selectivity studies together with the analysis of the hyperconjugation effect on the C_2_–H bonds provided by the fused cyclopropane moiety are shown in Figures S7–S10 of the SI for the reactions of substrates **S1**, **S2**, **S4**, and **S5**, respectively. The computed site-selectivity for the C(*sp*^3^)–H bond oxygenation of 1-methylbicyclo[4.1.0]heptane (**S3**) is displayed in Figure 4.

**Figure 5:**
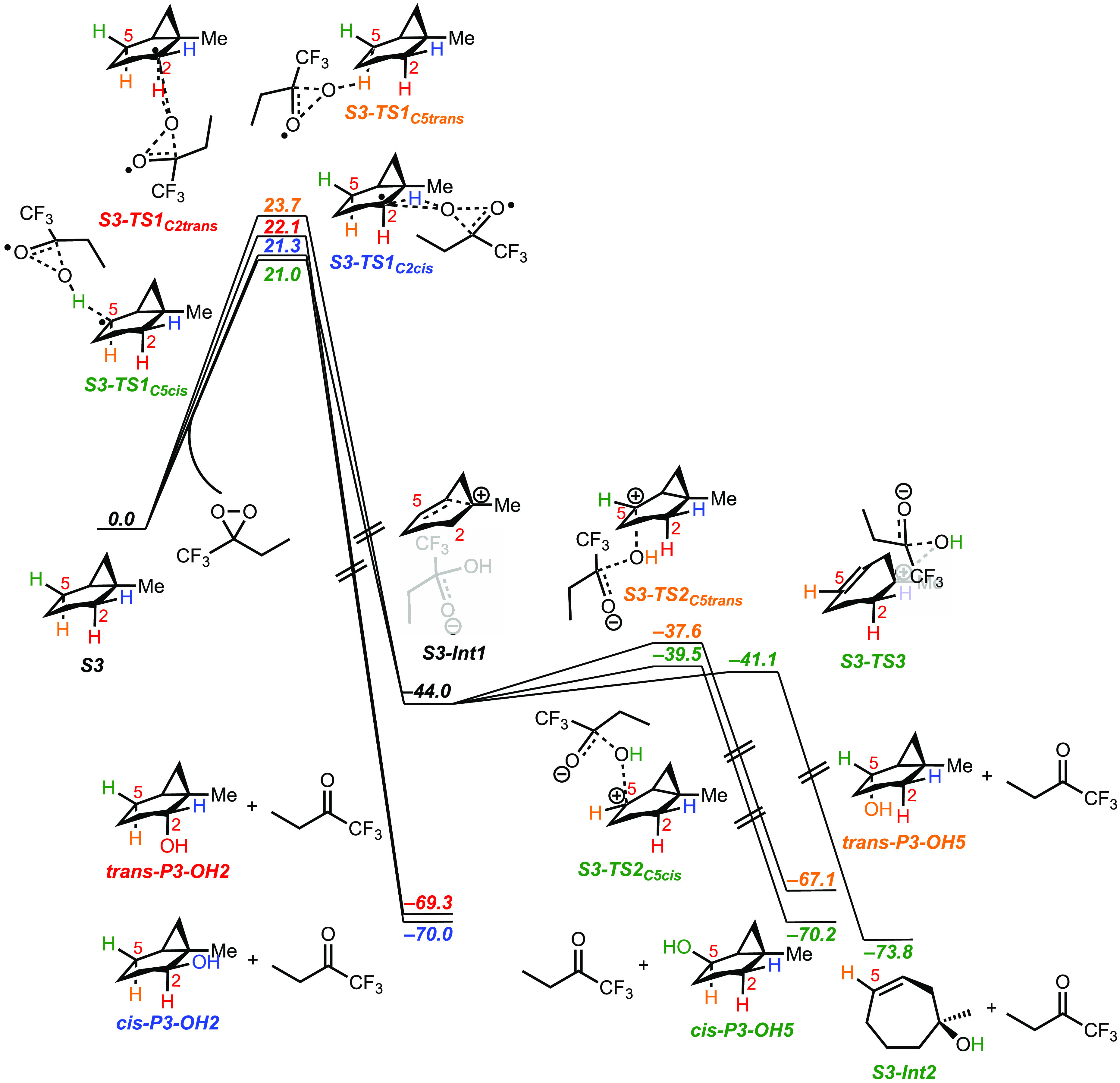
Energetics (in kcal mol^–1^) of C–H bond oxidation of **S3** promoted by ETFDO.

The transition structures for HAT from the C_2_–H and C_5_–H bonds of **S3** to ETFDO are displayed in the SI as Figure S11. The energetics of the hydroxylation mechanisms for each of the C–H bonds at C-2 and C-5 are displayed in Figure 5.

**Figure 6:**
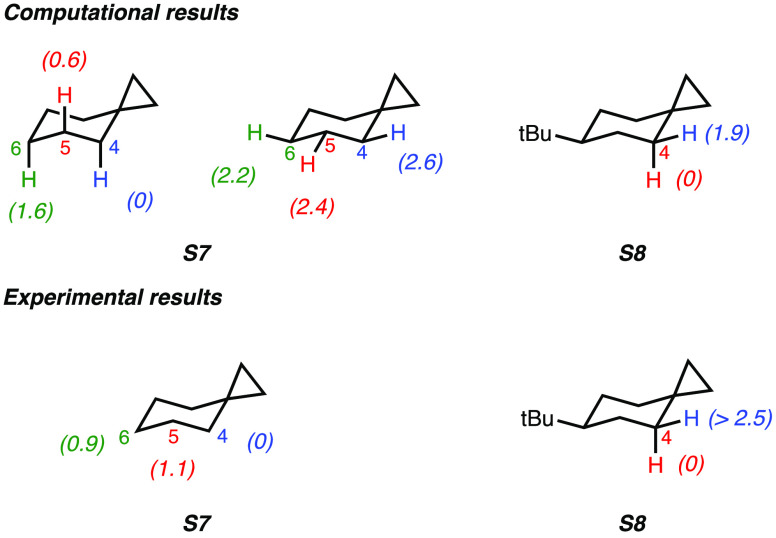
Difference in activation free energies (ΔΔ*G*^‡^, in kcal mol^–1^) for HAT from the C–H bonds of **S7** and **S8** to ETFDO: computational and experimental studies.

The computed site-selectivities for C(*sp*^3^)–H bond oxygenation of spiro[2.5]octanes **S7** and **S8** by ETFDO are displayed in Figure 6 along with the experimental ΔΔ*G*^‡^ values that are derived from the product distributions displayed in Scheme 4.

**Figure 7:**
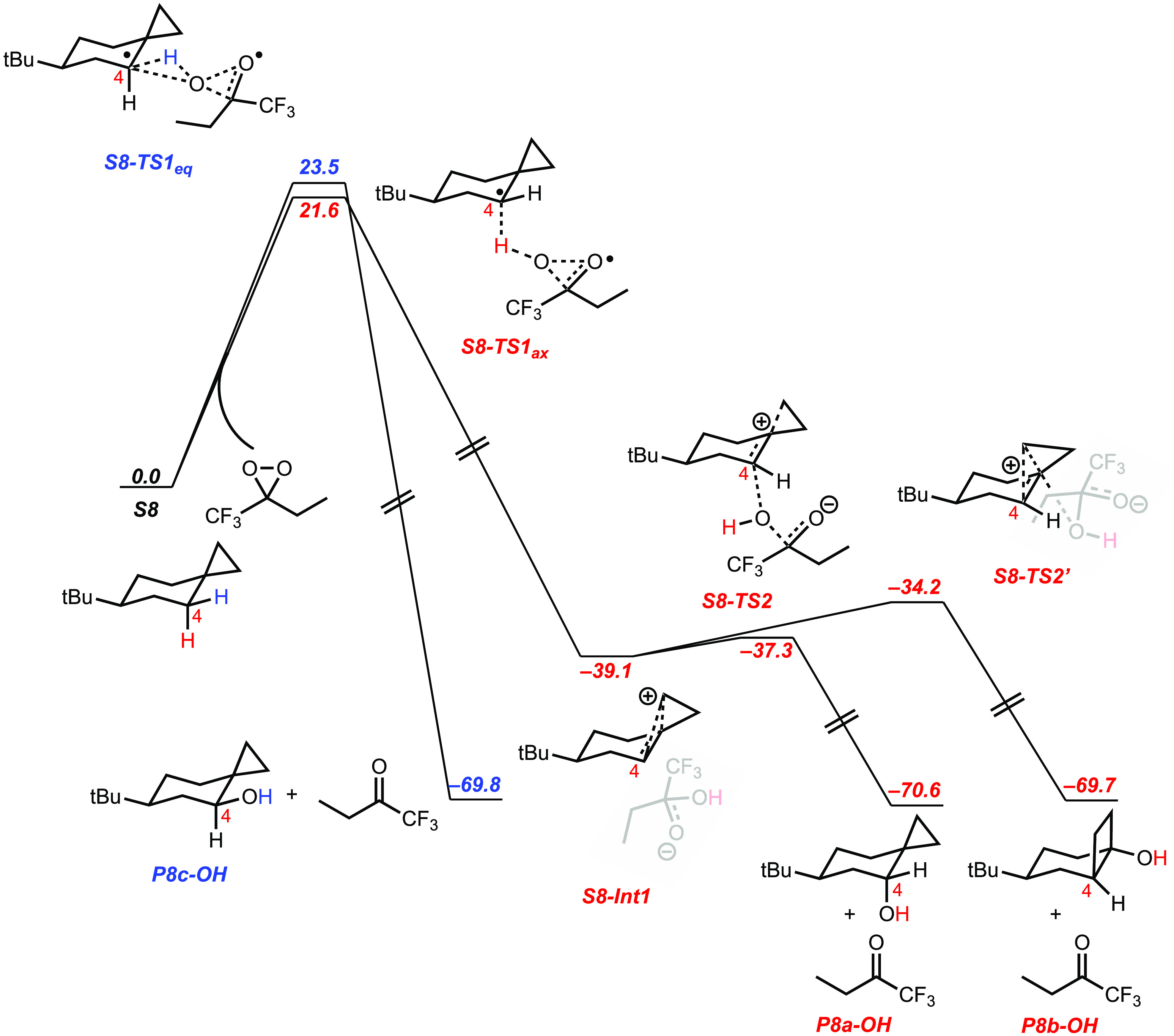
Energetics (in kcal mol^–1^) of C–H bond oxidation of **S8** by ETFDO.

The transition structures for HAT from various positions of **S7** and **S8** to ETFDO and the analysis of the hyperconjugation effect on the C_4_–H bonds provided by the spiro-cyclopropane moiety are displayed in the SI as Figures S12 and S13, respectively. The energetics of the hydroxylation mechanisms for the axial and equatorial C_4_–H bonds of **S8** are displayed in Figure 7.

The corresponding energy profiles of the hydroxylation mechanisms for the C_4_–H, C_5_–H, and C_6_–H bonds of **S7** are displayed in the SI as Figure S14.

## Discussion

### Oxygenation of Bicyclic Substrates (S1–S5, P2a-OH, and P2b-OH)

The products of the reaction of ETFDO with **S1–S5** are displayed in Scheme 3. With **S1**, **S2**, and **S4**, reactions carried out at low substrate conversion (3–6 h reaction time, 15–28% conversion) showed, in all cases, the predominant formation of the diastereomeric alcohol products deriving from C_2_–H bond hydroxylation, accompanied by smaller amounts of products deriving from oxygenation at the other methylenic sites. With all three substrates, no evidence for the formation of the ketone product deriving from overoxidation of the alcohols at C-2, and of products deriving from oxidation of the cyclopropane C–H bonds, was observed. The former observation can be accounted for on the basis of the strong hydrogen bond donor (HBD) ability of HFIP that, by engaging in hydrogen bonding with the hydroxyl group of the alcohol products, inverts the polarity of the adjacent C–H bond, deactivating this site toward HAT to the electrophilic ETFDO.^25^ The latter observation reflects the very high BDE of the cyclopropane C–H bonds,^26^ that are typically resistant to HAT-based functionalization. By increasing the reaction time (48 h for **S1**, 9 h for **S2** and **S4**), significantly higher conversions were obtained (80–87%), forming substantial amounts of the C-2 ketone. Products are oxygenated at the C-2 position of **S1**, **S2**, and **S4** with selectivities of 96%, 98%, and 72% respectively. The reaction of **S5** was carried out for a 9 h reaction time (86% conversion, 76% overall product yield), with the predominant formation of *trans*-bicyclo[6.1.0]nonan-2-ol (**P5a-OH**). These selectivities result from hyperconjugative stabilization, determined by the overlap of a cyclopropane Walsh C–C bonding orbital with the σ* orbital of the adjacent C_2_–H (Figure 1a).^6a^

**Figure 8:**
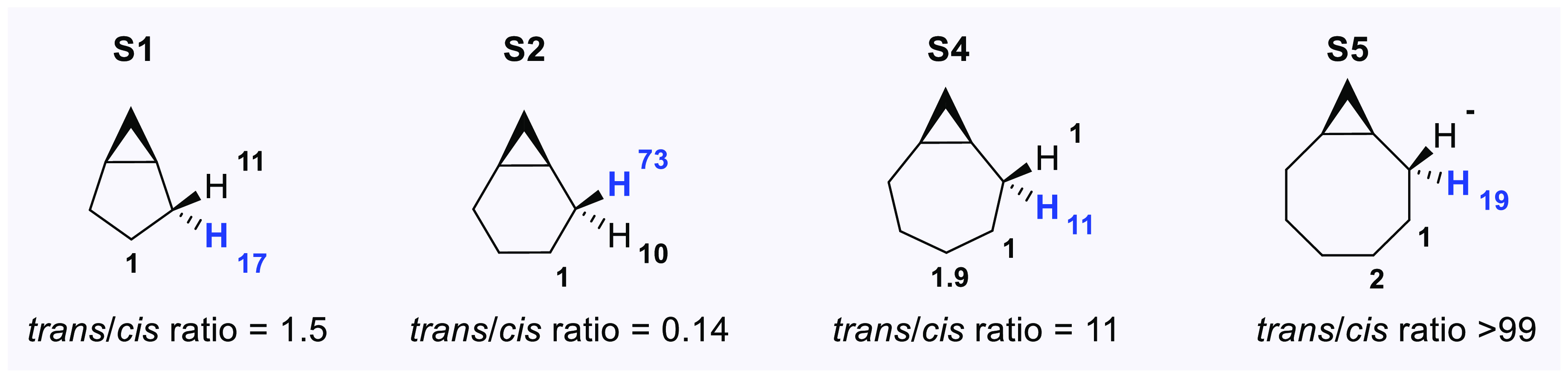
Normalized site-selectivities and diastereoselectivities observed in the hydroxylation of bicyclo[n.1.0]alkanes **S1**, **S2**, **S4**, and **S5** by ETFDO.

The analysis of the product distributions obtained for **S1**, **S2**, **S4**, and **S5**, under conditions where overoxidation is not observed, provides information about the hydroxylation diastereoselectivity. The normalized hydroxylation site-selectivities are displayed in Figure 8. The *trans*/*cis* ratios for C_2_–H hydroxylation are highlighted.

Preferential *trans* C_2_–H hydroxylation was observed for **S1**, **S4**, and **S5**, with the *trans*/*cis* ratio that increases with increasing ring size, reaching an upper limit with **S5** for which the product deriving from *cis* C_2_–H hydroxylation was not detected. Preferential *cis* C_2_–H hydroxylation was instead observed with **S2** (*trans*/*cis* = 0.14). Interestingly, similar diastereoselectivity patterns were observed in dihalocarbene insertions into the C_2_–H bonds of **S1** and **S2** (*trans*/*cis* = 2.8–4 and 0.23–0.25, respectively),^12^ because the same effects operate in dioxirane hydroxylation and carbene insertion reactions.

It is worth noting that cyclopropylcarbinyl stabilization leading to selectivity with **S2** also accounts for the diastereoselectivity observed in the oxidation employed in an intermediate step of the total synthesis of (+)-phorbol.^13^ Within the bicyclo[4.1.0]heptane structural motif (Figure 1a), selective hydroxylation at the α-C–H bond that is *cis* to the cyclopropane moiety was observed.

The diastereoselectivities were also explored by computational studies on the oxygenation of **S1**, **S2**, **S4**, and **S5** promoted by ETFDO. The activation free energy differences (ΔΔ*G*^‡^) for HAT from the C_2_–H bonds of these substrates to ETFDO are shown in Figure 3. The corresponding transition structures are presented in the SI (Figures S7–S10). Computational results show a strong preference for the oxygenation of C_2_–H over C_3_–H bonds, supporting the effect of hyperconjugation in C–H bond activation. Moreover, the studies of the oxidation selectivity align with experimental results. Figures S7–S10 highlight the hyperconjugative interaction by the cyclopropyl group when activating the *cis* and/or *trans* C_2_–H bond of **S1**, **S2**, **S4**, and **S5** toward HAT to ETFDO.

In the reaction of **S1**, σ* orbitals of both *cis* and *trans* C_2_–H bonds can interact with the Walsh orbitals activating these bonds toward HAT. As a result, the energy difference between *cis* and *trans* C_2_–H bond oxidation is only 0.5 kcal mol^–1^. The effect of hyperconjugation on *trans* C_2_–H bond activation is highlighted in Figure S7. Experiments did not differentiate the selectivity between *cis* and *trans* C_3_–H bonds. However, computations predict a preference for oxygenation of the *cis* over the *trans* C_3_–H bond (ΔΔ*G*^‡^ = 1.0 and 3.1 kcal mol^–1^, respectively).

With **S2**, the experimental and computational observation of a stronger activation of the *cis* C_2_–H bond over the *trans* one is also corroborated by the results obtained, under the same experimental conditions, in the oxidation of *cis*- and *trans*-bicyclo[4.1.0]heptan-2-ol (**P2a-OH** and **P2b-OH**, respectively) by ETFDO (Scheme 5a). With both substrates, exclusive formation of the corresponding ketone product (**P2-O**) in 9.2% and 33% yield, respectively, was observed, indicating that the latter alcohol is 3.6 times more reactive than the former one. **P2b-OH** displays a *cis* C_2_–H bond that benefits from hyperconjugative activation, whereas with **P2a-OH** the *trans* C_2_–H bond cannot benefit from a similar activation. Additional support is provided by the results obtained in the competitive oxidation of a 1:1 *trans*–*cis* mixture of bicyclo[4.1.0]heptan-2-ols (**P2a-OH** and **P2b-OH**) by ETFDO (Scheme 5b). 91% of **P2a-OH** and 66% of **P2b-OH**, together with an overall 40% yield of **P2-O**, were obtained, indicating that the latter alcohol is 3.8 times more reactive than the former one, showing excellent agreement between the two experiments.

With **S4** and **S5**, the *trans* C_2_–H bond (ΔΔ*G*^‡^ = 0 kcal mol^–1^) is the most activated toward HAT to ETFDO.

**Scheme 7:**
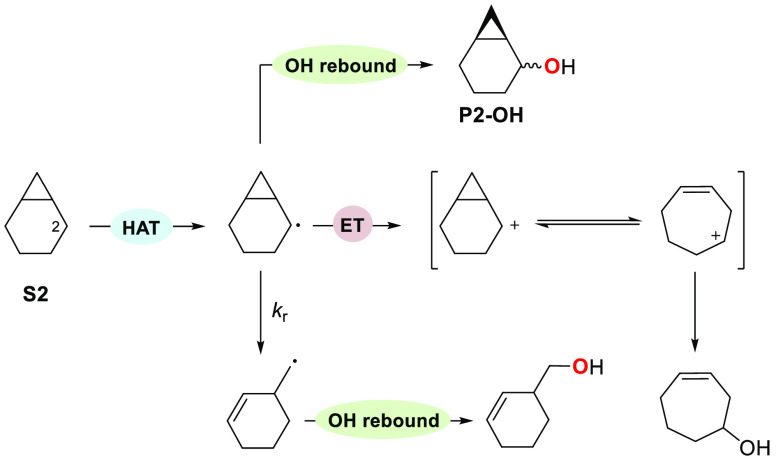
Groves Mechanism for the Oxygenation of **S2** Promoted by Cytochrome P450 Enzymes^15a^

Among the bicyclo[*n*.1.0]alkane series, the oxygenation of 1-methylbicyclo[4.1.0]heptane (**S3**) by ETFDO is particularly noteworthy. With this substrate, in addition to the alcohol and ketone products deriving from oxygenation at the most activated C–H bonds at C-2 (**P3-OH2** + **P3-O2**) and C-5 (**P3-OH5** + **P3-O5**) in 33.4% and 32.6% combined yield, respectively, *cis-* and *trans-*3-methyl-8-oxabicyclo[5.1.0]octan-3-ol (**P3-1**) were also observed among the reaction products in 13% combined yield (Scheme 3). Full details about the product distribution of this reaction can be found in the SI. The formation of products **P3-1** can be rationalized on the basis of the mechanism proposed by Groves and co-workers in the oxygenation of bicyclo[4.1.0]heptane (**S2**) promoted by cytochrome P450 enzymes.^15a^ The carbon radical formed following HAT from C-2 can undergo, in addition to the canonical OH rebound and radical rearrangement pathways, one-electron oxidation to give a cationic intermediate that, after rearrangement, is converted into the hydroxylated product by OH-transfer or nucleophilic capture by water (Scheme 7).

**Scheme 8:**
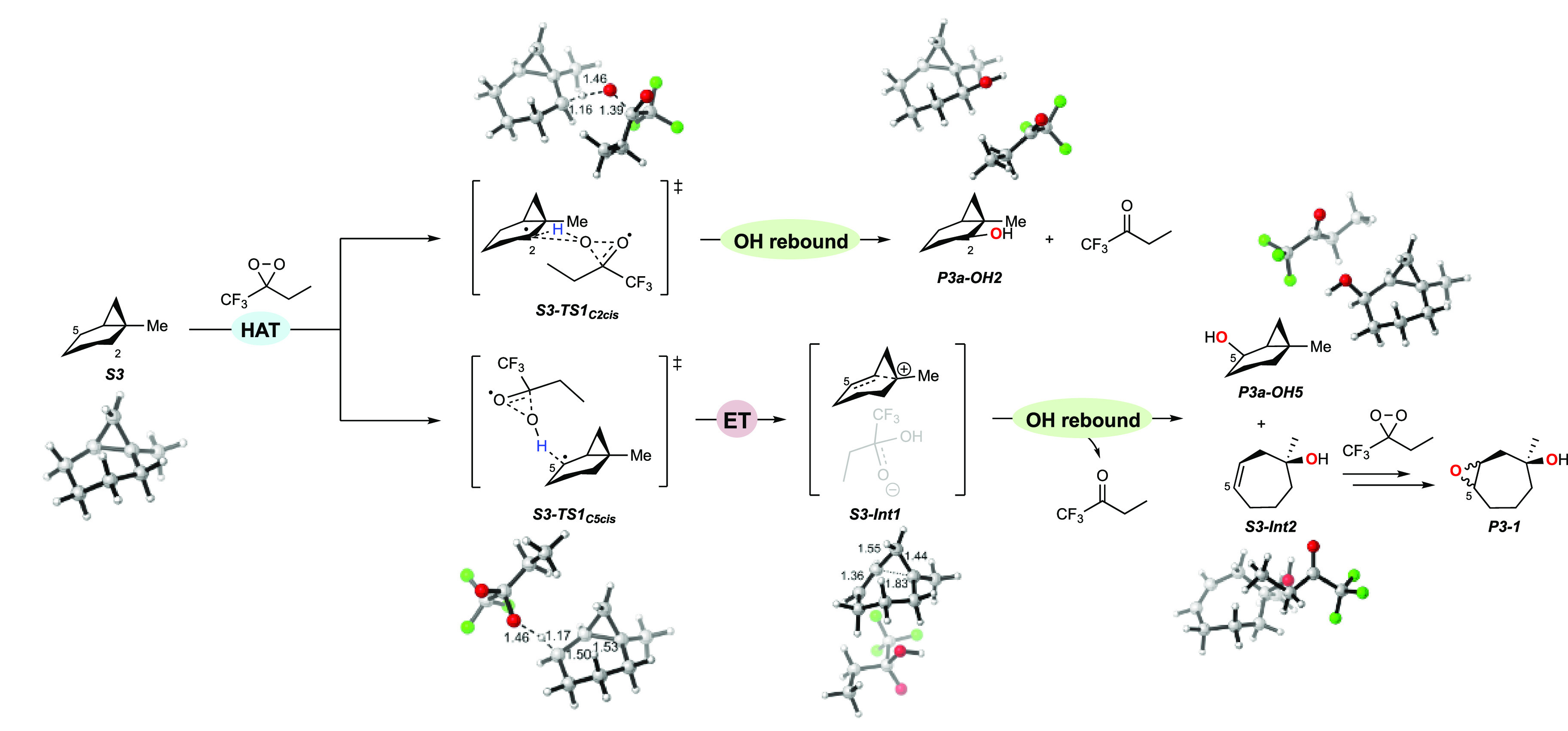
Proposed Mechanistic Pathways for the Oxygenation of **S3** Promoted by ETFDO For the sake of simplicity, only the pathways initiated by HAT from the *cis* C_2_–H and C_5_–H bonds are displayed.

An analogous mechanism is proposed for the oxidation of **S3**, where the formation of 1-methylcyclohept-3-en-1-ol is initiated by HAT from the C_5_–H bond. The intermediate alcohol product is then rapidly converted into **P3-1** as a diastereomeric mixture via epoxidation by ETFDO.^27^ This mechanistic hypothesis is supported well by the computational results. The oxidation site-selectivity (Figure 4) follows the order: *cis* C_5_–H (ΔΔ*G*^‡^ = 0 kcal mol^–1^), *cis* C_2_–H (ΔΔ*G*^‡^ = 0.3 kcal mol^–1^), *trans* C_2_–H (ΔΔ*G*^‡^ = 1.1 kcal mol^–1^), and *trans* C_5_–H (ΔΔ*G*^‡^ = 2.7 kcal mol^–1^), confirming the stronger activation of the *cis* α-C–H bonds over the corresponding *trans* ones. The free energy profiles (Figure 5) show concerted oxidation through asynchronous HAT from the *cis* and *trans* C_2_–H bonds via **S3-TS1**_**C2cis**_ and **S3-TS1**_**C2trans**_ (for which Δ*G*^‡^ = 21.3 and 22.1 kcal mol^–1^, respectively), coupled to OH-rebound to give products **P3-OH2**. A homoallylic tertiary carbocation intermediate (**S3-Int1**, – 44.0 kcal mol^–1^) is formed through asynchronous HAT from *cis* and *trans* C_5_–H bonds (**S3-TS1**_**C5cis**_ and **S3-TS1**_**C5trans**_: Δ*G*^‡^ = 21.0 and 23.7 kcal mol^–1^, respectively) coupled to electron transfer (ET). **S3-Int1** then undergoes hydroxylation at C-5 through **S3-TS2**_**C5cis**_ (−39.5 kcal mol^–1^) and **S3-TS2**_**C5trans**_ (−37.6 kcal mol^–1^), resulting in the formation of **P3-OH5**. Figure 5 shows that **S3-Int1** undergoes competitive hydroxylation at C-1 through **S3-TS3** (−41.1 kcal mol^–1^) to form 1-methylcyclohept-3-en-1-ol, **S3-Int2** (−73.8 kcal mol^–1^). **S3-Int2** is then converted into 3-methyl-8-oxabicyclo[5.1.0]octan-3-ols **P3-1** by oxygen atom transfer from ETFDO. The proposed mechanistic pathways for oxidation of **S3** by EFTDO are summarized in Scheme 8, which shows 3D figures of the intermediate and transition state structures.

Interestingly, rearranged products (**P3-1**) are formed via initial HAT at the C_5_–H bond, and analogous isomeric products are not produced by HAT at the C_2_–H bond. This difference is caused by the distinct stabilization between the tertiary and secondary homoallylic cations. Hyperconjugation from the C-1 methyl group supports the emerging cationic intermediate at C-1 after HAT at the C_5_–H bond.^28,29^ Supportive evidence in favor of an ET pathway was also gained by investigating solvent effects on the oxygenation of **S3** by ETFDO. By analyzing the products deriving from initial HAT at C-5, a decrease in the ratio between rearranged (**P3-1**) and unrearranged (**P3-OH5** and **P3-O5**) products with decreasing solvent HBD ability was observed, i.e., going from HFIP to 2,2,2-trifluoroethanol (TFE) and MeCN (**P3-1**/(**P3-OH5** + **P3-O5**) = 0.40, 0.16, and <0.01, respectively) (see SI, Table S9). This behavior can be associated with the strong HBD ability of fluorinated alcohols that, compared to non-HBD or weaker HBD solvents, can promote ET reactions via an increase in the oxidizing power of ET reagents and the ability to stabilize cationic intermediates.^30^

### Oxygenation of Spirocyclic Substrates (S7, S8, P8a-OH, and P8c-OH)

The results obtained in the oxidation of spiro[2.5]octane (**S7**) and 6-*tert*-butylspiro[2.5]octane (**S8**) promoted by ETFDO were compared with those obtained for the corresponding reaction of 1,1-dimethylcyclohexane (**S6**) taken as a reference substrate, and are displayed in Scheme 4. With **S6**, the reaction carried out for 48 h, followed by treatment with chromic acid, afforded the ketone products deriving from oxidation at C-2 (**P6-O**), C-3 (**P6-O3**), and C-4 (**P6-O4**), in 14%, 21%, and 11% yield, respectively. Under the same conditions, the reaction of **S7** led to the ketone products deriving from oxidation at C-4 (**P7-O**), C-5 (**P7-O5**), and C-6 (**P7-O6**), in 66%, 9.2%, and 7% yield, respectively, accompanied by the rearranged product bicyclo[4.2.0]octan-1-ol (**P7b-OH**) in 4.8% yield. With **S8**, the reaction mixture was not subjected to follow-up treatment with chromic acid, and the reaction carried out for 3 h showed the exclusive formation of the axial alcohol at C-4 (**P8a-OH**) in an 8.8% yield. By increasing the reaction time to 48 h, **P8a-OH** was formed in 54% yield, accompanied by the corresponding ketone (**P8-O**) and the rearranged alcohol *cis*-4-(*tert*-butyl)-bicyclo[4.2.0]octan-1-ol (**P8b-OH**) in 20% and 5.5% yield, respectively. With this substrate, oxygenation products at C-5 and C-6 as well as the equatorial alcohol at C-4, **P8c-OH**, were never observed. The formation of the rearranged alcohols **P7b-OH** and **P8b-OH** in the oxygenation of **S7** and **S8** by ETFDO (Scheme 4) provides conclusive evidence for the involvement of a cationic intermediate, uncovering the contribution of ET pathways to the overall reactivity.^15a,17^ This hypothesis is further supported by computational studies.

The energetics of the oxidation of **S8** by ETFDO are shown in Figure 7. The axial C_4_–H bond undergoes asynchronous HAT to ETFDO through **S8-TS1**_**ax**_ (21.6 kcal mol^–1^), which is coupled to ET to directly form the ion-pair **S8-Int1** (−39.1 kcal mol^–1^). **S8-Int1** undergoes either OH rebound via (**S8-TS2**, −37.3 kcal mol^–1^) or hydroxylation at C–3 via **S8-TS2′** (−34.2 kcal mol^–1^). This observation accounts for the formation of **P8b-OH** through charged species **S8-Int1** (Scheme 4).^31^ The activation energy of **S8-TS2′** is slightly higher in comparison with **S8-TS2** (ΔΔ*G*^‡^ = 3.1 kcal mol^–1^). This energy difference qualitatively matches the experiment, explaining the low yield of **P8b-OH**. Hydroxylation of the equatorial C_2_–H bond (**S8-TS1**_**eq**_, 23.5 kcal mol^–1^) occurs concertedly without generating charged intermediates.

**Figure 9:**
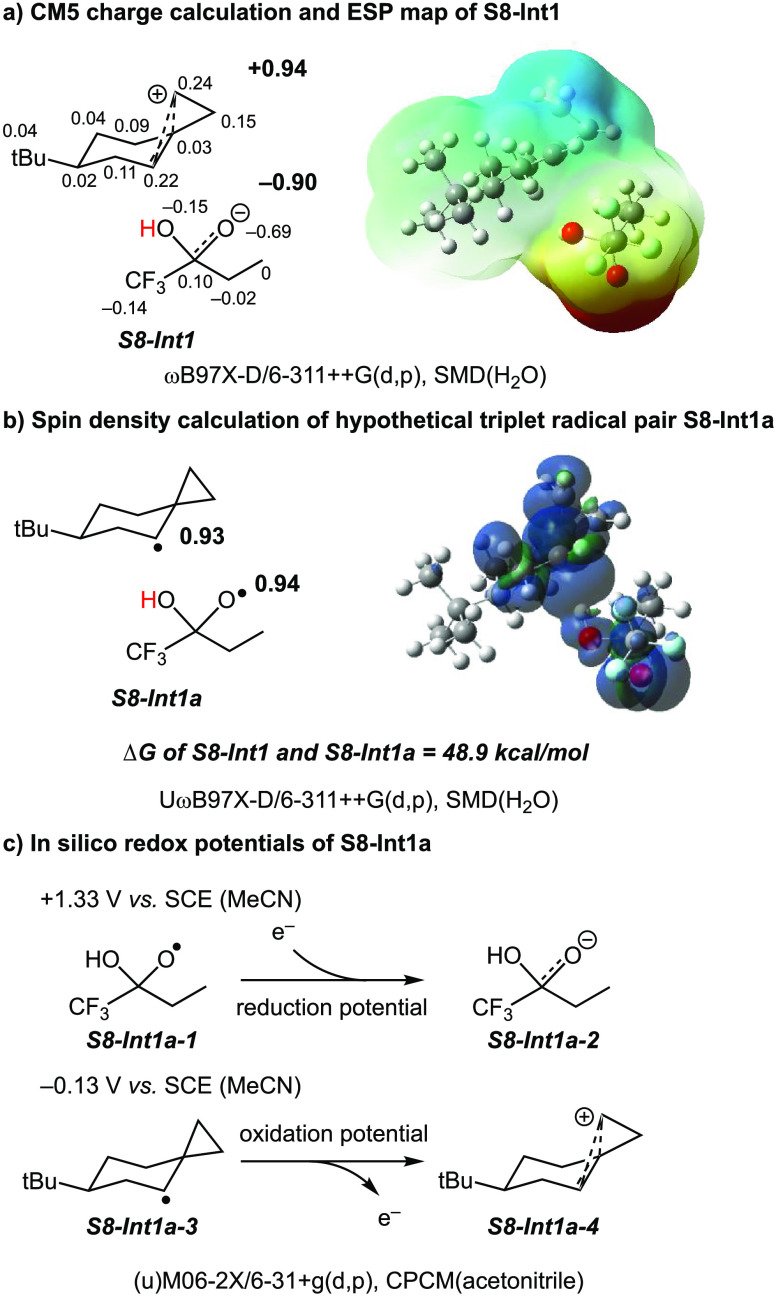
(a) Charge distribution of **S8-Int1** by CM5 and the electrostatic potential on a constant electron density surface. The regions of positive and negative potential are indicated in blue and red. (b) Spin density of the hypothetical triplet radical pair **S8-Int1a**. (c) In silico redox potentials of 1,1,1-trifluoro-2-hydroxybutoxy and 6-(*tert*-butyl)spiro[2.5]octan-4-yl radicals.

DFT calculations play a pivotal role by providing a qualitative approximation of the reaction outcomes. In a previous study,^18b^ molecular dynamics revealed a 90% barrierless oxygen-rebound mechanism and 10% radical pair formation, while DFT predicted only a barrierless oxygen-rebound mechanism. This highlights the value of DFT and IRC in capturing the essence of the reaction mechanism, albeit with a degree of approximation. In order to confirm that **S8-Int1** is the ion-pair intermediate, the CM5 calculation is employed to check the distribution of charges (Figure 9). The charge is evenly distributed in the 6-*tert*-butylspiro[2.5]octanylium cation (+0.94) and trifluoro-2-hydroxybutan-2-olate anion (−0.90). Moreover, a hypothetical triplet radical pair **S8-Int1a** is noticeably unstable compared to ion-pair **S8-Int1** by 48.9 kcal mol^–1^. An open-shell singlet radical pair is not obtained in the computations with ωB97X-D/6-311++G(d,p)/SMD(H_2_O). Open-shell initial guesses led to the closed-shell result. Consequently, a hypothetical triplet radical pair **S8-Int1a** was employed to compare its energies with the **S8-Int1** ion-pair. It is also worth mentioning that formation of a delocalized cation following ET within the hypothetical radical pair **S8-Int1a** strongly contributes to the reaction exergonicity. Isodesmic reaction calculations show a 10.7 kcal mol^–1^ thermodynamic advantage for delocalized **S8-Int1** over the corresponding localized secondary carbocation (see Table S13 and Scheme S2 in the SI).

We also determined in silico redox potentials for the formation of 1,1,1-trifluoro-2-hydroxybutan-2-olate and 6-(*tert*-butyl)spiro[2.5]octan-4-ylium from the hypothetical radical pair **S8-Int1a** (Figure 9c). We found that the reduction potential of the 1,1,1-trifluoro-2-hydroxybutoxy radical is +1.33 V vs SCE (MeCN), and the oxidation potential from 6-(*tert*-butyl)spiro[2.5]octan-4-yl radical to 6-(*tert*-butyl)spiro[2.5]octan-4-ylium cation is −0.13 V vs SCE (MeCN). The redox potentials suggest the formation of the charged species via an exergonic redox process.

Supportive experimental evidence in favor of an ET pathway was gained from the study of the solvent effects on the oxidation reaction. Oxygenation of this substrate by ETFDO was studied in HFIP, TFE, and MeCN. As the solvent HBD ability was reduced, the ratio between rearranged (**P8b-OH**) and unrearranged (**P8a-OH** + **P8-O**) products was diminished, leading to the following **P8b-OH**/(**P8a-OH** + **P8-O**) ratios: 0.065, 0.028, <0.01, for HFIP, TFE, and MeCN, respectively (see SI, Table S10), pointing again toward the ability of fluorinated alcohols to promote ET reactions via an increase in the oxidizing power of ET reagents and to stabilize cationic intermediates.^30,32^

**Scheme 9:**
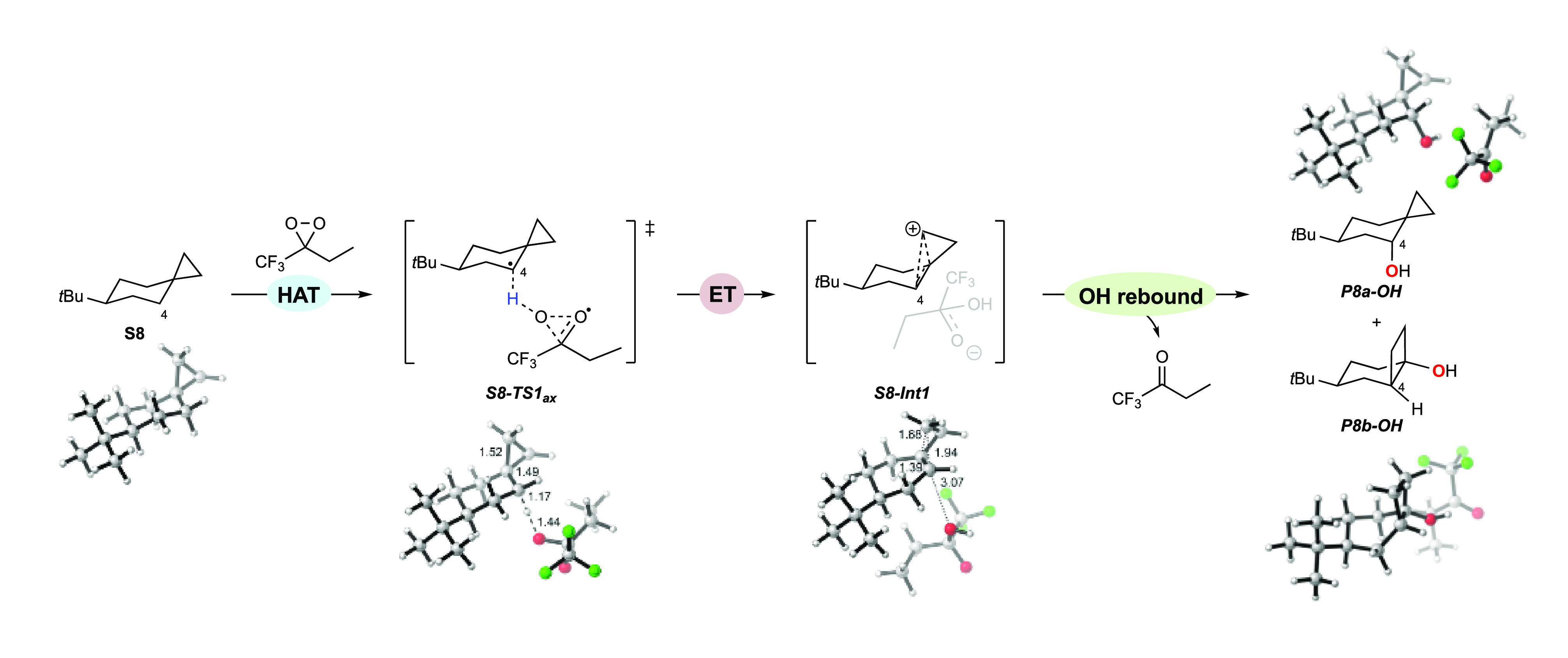
Proposed Mechanism for the Oxygenation of **S8** Promoted by ETFDO.

Based on these mechanistic studies and on previous findings,^17^ the oxidation mechanism of **S8** by ETFDO is proposed in Scheme 9. An analogous mechanism is found for the oxygenation pathways initiated by HAT from the C_4_–H bond of **S7** (Figure S14).

Grabovskiy et al. presented a concerted molecule-induced homolytic/rebound process of cage hydrocarbons using dioxiranes.^33^ Notably, our findings suggest that the generation of the cationic intermediate is associated with a specific stabilizing hyperconjugative interaction between the incipient carbon radical and the cyclopropane C–C bonding orbitals. This causes ET to the incipient 1,1,1-trifluoro-2-hydroxy-2-butoxyl radical.^28^

The ΔΔ*G*^‡^ values for HAT from the C–H bonds of **S7** and **S8** to ETFDO are displayed in Figure 6. HAT from the C_4_–H bond of **S7** presents the lowest energy barrier (ΔΔ*G*^‡^ = 0 kcal mol^–1^), in comparison with the energy barriers for C_5_–H and C_6_–H bonds (ΔΔ*G*^‡^ = 0.6 and 1.6 kcal mol^–1^, respectively). Furthermore, we find that the activation barriers of axial C–H bonds are lower than those of equatorial ones. The transition state structures are shown in Figure S12 in the SI. In **S7**-**TS1**_**C4ax**_, hyperconjugation leads to a slightly extended C_1_–C_2_ distance (1.52 Å) and a reduced C_1_–C_4_ distance (1.49 Å), differing from the other transition states that lack Walsh orbital interactions. Moreover, efficient hyperconjugation between the axial C_4_–H bond and the Walsh orbital in the transition state **S7-TS1**_**C4ax**_ is evidenced.

With **S8**, oxygenation of the axial C_4_–H bond is favored over the equatorial one by 1.9 kcal mol^–1^, in good agreement with the experimental studies. Based on the analysis of the transition state structures, a hyperconjugative interaction by cyclopropane Walsh orbitals lowers the barrier of the axial C_4_–H bond. Compared to **S8-TS1**_**eq**_, **S8-TS1**_**ax**_ exhibits a slightly longer C_1_–C_2_ distance (1.52 Å) and a shorter C_1_–C_4_ distance (1.49 Å) due to hyperconjugation.

The observation of a stronger hyperconjugative activation of the axial C_4_–H bond over the equatorial one is also corroborated by the results obtained, under the same experimental conditions, in the oxidation of *trans*- and *cis*-6-*tert*-butylspiro[2.5]octan-4-ol (**P8a-OH** and **P8c-OH**, respectively) by ETFDO (Scheme 6a). With both substrates, exclusive formation of the ketone product (**P8-O**) in 5% and 22% yield, respectively, was observed, indicating that the latter alcohol is 4.4 times more reactive than the former one. **P8c-OH** displays an axial C_4_–H bond that benefits from hyperconjugative activation, whereas with **P8a-OH** the equatorial C_4_–H bond C-4 cannot benefit from a similar activation. Additional support comes again from the results obtained in the competitive oxidation of a 1:1 *trans*–*cis* mixture of 6-*tert*-butylspiro[2.5]octan-4-ols (**P8a-OH** and **P8c-OH**) promoted by ETFDO (Scheme 6b): 94% recovery of **P8a-OH** and 74% recovery of **P8c-OH**, together with an overall 31% yield of **P8-O** were obtained, indicating that the latter alcohol is 4.3 times more reactive than the former one, showing an excellent agreement between the two experiments.

**Figure 10:**
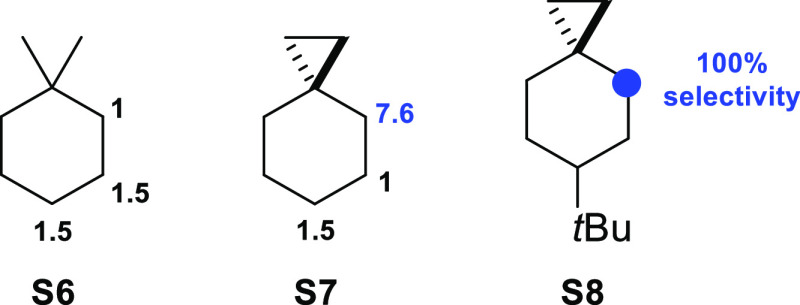
Normalized site-selectivities observed in the oxygenation of 1,1-dimethylcylohexane (**S6**), spiro[2.5]octane (**S7**) and 6-*tert*-butylspiro[2.5]octane (**S8**) promoted by ETFDO.

For the site-selectivities observed in the reactions of ETFDO with substrates **S6–S8**, the normalized product distributions are displayed in Figure 10. With **S6**, comparable selectivities were observed for the three methylenic sites (C-2:C-3:C-4 = 1.0:1.5:1.5). The slightly lower selectivity for oxygenation at C-2 over C-3 and C-4 can be reasonably explained on the basis of steric effects, where the presence of the two methyl groups limits the accessibility of the adjacent C_2_–H bonds to ETFDO.

With **S7**, taking into account that the rearranged alcohol product **P7b-OH** derives from initial HAT from the C_4_–H bond, the normalized product distributions (C-4:C-5:C-6 = 7.6:1.0:1.5) point toward a significant activation of the C_4_–H bonds compared to the other methylenic sites. These results are in good agreement with those obtained previously in the oxidation of **S7** promoted by the H_2_O_2_/(*S*,*S*)-Fe(pdp) and H_2_O_2_/(*S*,*S*)-Mn(^TIPS^pdp) systems and by TFDO.^11e,11f,17^ As mentioned above, this behavior reflects activation of the axial C_4_–H bonds via overlap with the Walsh C–C cyclopropane bonding orbitals. The site-selectivity observed in the oxygenation of **S8**, for which exclusive formation of products deriving from initial HAT at this site, reflects the synergistic cooperation of two effects: hyperconjugative C_4_–H bond activation together with C_5_–H and C_6_–H bond deactivation by torsional and steric effects determined by the presence of the bulky *tert*-butyl group at C-6.^17,19^

## Conclusions

The results of product and computational studies on the C(*sp*^3^)–H bond oxygenation of bicyclic and spirocyclic hydrocarbons bearing cyclopropyl moieties promoted by ETFDO have led to a deeper understanding of the factors that govern selectivity in these processes. Activation of the C–H bonds that are α to the cyclopropyl group occurs when there is strong overlap between the cyclopropane Walsh C–C bonding orbitals and the C–H σ* orbitals. Diastereoselective hydroxylation is typically observed, reflecting preferential activation of one α-C–H bond, with the exclusive detection of a single diastereoisomer in the reactions of bicyclo[6.1.0]nonane (**S5**) and 6-*tert*-butylspiro[2.5]octane (**S8**). The experimental site-selectivities and diastereoselectivities are paralleled by the calculated activation free energies for the corresponding reaction pathways. The detection of rearranged oxygenation products in the oxidation of 1-methylbicyclo[4.1.0]heptane (**S3**), spiro[2.5]octane (**S7**), and 6-*tert*-butylspiro[2.5]octane (**S8**) provides unambiguous evidence for the involvement of cationic intermediates in these reactions, representing the first examples on the operation of ET pathways in dioxirane-mediated C(*sp*^3^)–H bond oxygenations.^34,35^ With these substrates, calculations predict the direct formation of an intermediate ion pair via HAT from a substrate C–H bond to ETFDO coupled to ET, highlighting the role of specific stabilizing interactions able to assist cation formation and divert the reaction from the canonical HAT/rebound pathway.

## Supporting Information Available

Details on the preparation of the substrates, their oxidation reactions by ETFDO, on the isolation and characterization of reaction products, and on the computational studies. (PDF)
