# Supplementary material for: Radical and Cationic Pathways in C(sp3)–H Bond Oxygenation by Dioxiranes of Bicyclic and Spirocyclic Hydrocarbons Bearing Cyclopropane Moieties
Source: J Am Chem Soc. 2023 Oct 24;145(44):24021–34. doi: 10.1021/jacs.3c07163 (PMC10636757; doi:10.1021/jacs.3c07163)
Supplement: Supplementary file 1 — ja3c07163_si_001.pdf [file ja3c07163_si_001.pdf]

Supporting Information

Radical and Cationic Pathways in C(sp^3)–H Bond Oxygenation by Dioxiranes of Bicyclic and Spirocyclic Hydrocarbons Bearing Cyclopropane Moieties

Marco Galeotti,[1,2§] Woojin Lee,[3§] Sergio Sisti,[1] Martina Casciotti,[1] Michela Salamone,[1] K. N. Houk,[3] and Massimo Bietti [1]**

[1] Dipartimento di Scienze e Tecnologie Chimiche, Università "Tor Vergata", Via della Ricerca Scientifica, 1 I-00133 Rome, Italy.

[2] QBIS Research Group, Institut de Química Computacional i Catàlisi (IQCC) and Departament de Química, Universitat de Girona, Campus Montilivi, Girona E-17071, Catalonia, Spain.

[3] Department of Chemistry and Biochemistry, University of California, Los Angeles, California 90095, United States.

[§]M. G. and W. L. contributed equally to this work.

CONTENTS

S1. EXPERIMENTAL SECTION **S3**

S1.1. Instrumentation S3

S1.2. Materials S3

S2. SYNTHESIS OF THE SUBSTRATES **S4**

S3. OXIDATIONS PROMOTED BY ETFDO **S7**

S3.1. General procedure S7

S3.2. Results S8

S4. ISOLATION AND CHARACTERIZATION OF THE OXIDATION PRODUCTS **S13**

S5. COMPUTATIONAL SECTION **S24**

S5.1. Calculated Energetics S24

S5.2. Calculated Cartesian coordinates S26

S5.3. Supplementary figures of computational results S106

S6. REFERENCES **S115**

S1. Experimental Section

S1.1. Instrumentation

Dioxirane-promoted oxidation were carried out under magnetic stirring at constant temperature (T = 0 °C) by means of a Julabo F34 Refrigerated Circulator connected to the outer casing of a pyrex glass vessel containing the reaction mixture.

Gas-chromatographic analyses were carried out using an Agilent 7820A gas-chromatograph equipped with a stationary phase HP-5 30 m × 0.320 mm × 0.25 μm capillary column.

GC-MS analyses were performed using a Shimadzu GCMS-QP2010 SE gas chromatograph mass spectrometer equipped with a Supelco spb-5 30 m x 0.25 mm x 0.25 μm capillary column.

^{1}H-NMR and ^{13}C-NMR spectra were recorded on Bruker Avance 400 and Bruker Avance 700 MHz spectrometers. Spectra were referenced to tetramethylsilane (TMS).

S1.2. Materials

Reagents and solvents used are of commercially available reagent quality unless stated otherwise and are purchased from SDS, Aldrich, Scharlab and Fluorochem. Sigma-Aldrich HPLC-grade acetonitrile was employed for oxidation catalysis. All reagents used are of the highest commercial quality available unless stated otherwise.

S2. Synthesis of the substrates

The synthesis of the substrates **S1-S5**, **S7** and **S8** was carried out according to a reported procedure (**Scheme S1**).[1] **S6** was a commercial sample of the highest quality available and was used as received.

Alkenes used for the synthesis of **S1-S5** and **S7** were of the highest commercial quality available and were used as received. The commercially unavailable 1-(*tert*-butyl)-4-methylenecyclohexane used for the synthesis of **S8** was prepared by Wittig olefination of the parent ketone according to a reported procedure.[1]

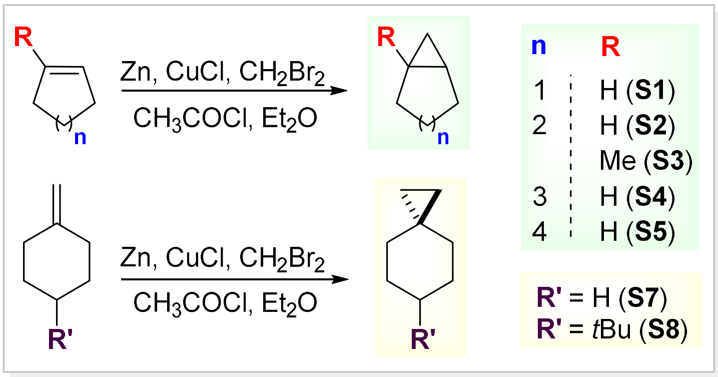

Scheme S1. Cyclopropanation of alkenes *via* Simmons-Smith reaction.

Zinc (4 eq), copper (I) chloride (0.4 eq), dibromomethane (1 eq) and 15 ml of diethyl ether were introduced into a 100 ml three neck round-bottom flask, equipped with a condenser system and a pressure-equalizing dropping funnel. After addition of acetyl chloride (0.08 eq), the flask is placed in a pre-heated oil bath (45-50 °C) under vigorous reflux and stirring. When the solution turned dark (about 10 minutes), ~0.04 mol of the alkene (1 eq) diluted in 5 ml of diethyl ether were added dropwise for 15 minutes. The remaining dibromomethane (2 eq) diluted in 5 ml of diethyl ether was added dropwise for 30 minutes under manageable reflux. When substrate was fully converted (generally 2 hours after the addition of all the reagents), the flask was cooled in an ice bath and 25 ml of a saturated ammonium chloride aqueous solution were added dropwise (the reaction was strongly exothermic) through the dropping funnel. The residual solid zinc was removed by vacuum filtration and the aqueous layer of the filtrate was washed twice with n-pentane (2 × 25 ml). The combined organic layers were washed twice with a 2.5 M sodium hydroxide aqueous solution (2 × 50 ml), one time with brine (50 ml) and were dried over anhydrous sodium sulfate. Due to product volatility (in particular for hydrocarbons **S2-S4, S7**), *n*-pentane and diethyl ether were carefully removed by Hempel fractional distillation and a controlled vacuum distillation (vacuum applied by water pump and controlled by means of a pressure gauge) of the remaining oil gave the pure cyclopropyl derivative (≥ 98%). For the purification of the bicyclo[3.1.0]hexane (**S1**) (boiling point ~ 80 °C) the solvent was completely removed by distillation with a Vigreux column and the fractionation of the residue yielded the pure **S1**.[2]

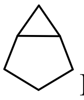

Bicyclo[3.1.0]hexane (**S1**). Prepared following the general procedure on 0.059 mol of cyclopentene. Fractional distillation of the crude afforded **S1** as a colorless liquid (1.0 g, 0.012 mol, 20% yield). Spectroscopic data are consistent with those reported in the literature.[2] ^{1}H NMR (400 MHz, $CDCl_3$) δ, ppm: 1.75 – 1.61 (m, 4H), 1.54 – 1.47 (m, 1H), 1.24 – 1.17 (m, 2H), 1.15 – 1.04 (m, 1H), 0.26 (td, *J* = 7.9, 4.7 Hz, 1H), 0.09 (q, *J* = 4.0 Hz, 1H).

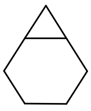

Bicyclo[4.1.0]heptane (**S2**)**.** Prepared following the general procedure on 0.041 mol of cyclohexene. Vacuum distillation (60 °C, 160 mmHg) of the crude afforded **S2** as a colorless liquid (1.3 g, 0.014 mol, 34% yield). Spectroscopic data are consistent with those reported in the literature.[3] ^{1}H-NMR (400 MHz, $CDCl_3$) δ, ppm: 1.95 – 1.81 (m, 2H), 1.69 – 1.57 (m, 2H), 1.34 – 1.10 (m, 4H), 0.86 (dddt, *J* = 9.0, 6.7, 4.2, 1.1 Hz, 2H), 0.54 (td, *J* = 8.9, 4.3 Hz, 1H), 0.02 (td, *J* = 5.3, 4.4 Hz, 1H).

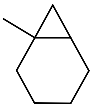

1-Methylbicyclo[4.1.0]heptane (**S3**). Prepared following the general procedure on 0.042 mol of 1-methylcyclohexene. Vacuum distillation (56 °C, 80 mmHg) of the crude afforded **S3** as a colorless liquid (2.0 g, 0.018 mol, 43% yield). Spectroscopic data are consistent with those reported in the literature.[4] ^{1}H NMR (400 MHz, $CDCl_3$) δ, ppm: 1.97 – 1.86 (m, 1H), 1.74 (dt, *J* = 13.4, 5.3 Hz, 1H), 1.55 – 1.47 (m, 2H), 1.39 – 1.30 (m, 1H), 1.28 – 1.21 (m, 1H), 1.20 – 1.07 (m, 2H), 1.04 (s, 3H), 0.66 (m, 1H), 0.29 (dd, *J* = 9.0, 4.1 Hz, 1H), 0.15 (t, *J* = 4.7 Hz, 1H).

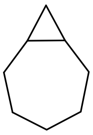

Bicyclo[5.1.0]octane (**S4**). Prepared following the general procedure on 0.021 mol of cycloheptene. Vacuum distillation (90 °C, 160 mmHg) of the crude afforded **S4** as a colorless liquid (0.95 g, 0.0086 mol, 41% yield). Spectroscopic data are consistent with those reported in the literature.[5] ^{1}H NMR (400 MHz, $CDCl_3$) δ, ppm = 2.21 – 2.06 (m, 2H), 1.71 (m, 2H), 1.36 (m, 2H), 1.15 (dtt, *J* = 13.3, 12.1, 3.1 Hz, 1H), 0.96 – 0.85 (m, 2H), 0.84 – 0.75 (m, 2H), 0.66 (ddd, *J* = 8.5, 7.4, 4.3 Hz, 1H), 0.06 (d, *J* = 4.7 Hz, 1H).

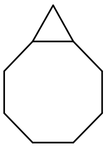

Bicyclo[6.1.0]nonane (**S5**). Prepared following the general procedure on 0.039 mol of cyclooctene. Vacuum distillation (71 °C, <1 mmHg) of the crude afforded **S5** as a colorless liquid (2.1 g, 0.017 mol, 44% yield). Spectroscopic data are consistent with those previously reported in the literature.[3] ^{1}H NMR (400 MHz, $CDCl_3$) δ, ppm = 2.03 – 1.92 (m, 2H), 1.68 (m, 2H), 1.60 – 1.49 (m, 3H), 1.46 – 1.28 (m, 4H), 0.91 (m, 2H), 0.67 – 0.48 (m, 3H), -0.29 (td, *J* = 4.9, 3.7 Hz, 1H).

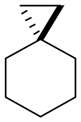

Spiro[2.5]octane (**S7**). Prepared following the general procedure on 0.042 mol of methylenecyclohexane. Vacuum distillation (73 °C, 80 mmHg) of the crude afforded **S7** as a colorless liquid (2.0 g, 0.018 mol, 43% yield). Spectroscopic data are consistent with those reported in the literature.[1] ^{1}H-NMR (400 MHz, $CDCl_3$) δ, ppm: 1.5 (m, 6H), 1.25 (m, 4H), 0.19 (m, 4H).

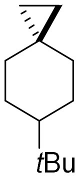

6-*tert*-Butylspiro[2.5]octane (**S8**). Prepared following the general procedure on 0.030 mol of 1-(*tert*-butyl)-4-methylenecyclohexane. Vacuum distillation (80 °C, <1 mmHg) of the crude afforded **S8** as a colorless liquid (2.0 g, 0.012 mol, 40% yield). Spectroscopic data are consistent with those reported in the literature.[1] ^{1}H-NMR (400 MHz, $CDCl_3$) δ, ppm: 1.80 – 1.68 (m, 4H), 1.22 – 1.08 (m, 2H), 1.07 – 0.95 (m, 1H), 0.89 (m, 11H), 0.32 – 0.23 (m, 2H), 0.23 – 0.12 (m, 2H).

S3. Oxidation promoted by ETFDO

S3.1. General procedure

The oxidation of hydrocarbons bearing cyclopropyl moieties was performed employing *in situ* generated dioxiranes, according to the following procedure. This protocol was optimized in our laboratory,[6] building on previous work by Hilinski and coworkers.[7]

All the experiments were carried out under magnetic stirring at T = 0 °C. 62 mg (0.1 mmol, 1 eq) of potassium peroxymonosulfate triple salt ($2KHSO_5 \cdot KHSO_4 \cdot K_2SO_4$, oxone) and 33 mg (0.4 mmol, 4 eq) of $NaHCO_3$ were placed in a pyrex glass tube equipped with an outer containing for circular refrigeration, then 1.0 mL of bidistilled water was added. When the salts were solubilized, 1.7 mg (0.005 mmol, 5 mol%) of Bu_4NHSO_4 and 3 mL of 1,1,1,3,3,3-hexafluoro-2- propanol (HFIP) were added (alternatively 3 ml of TFE or 1.5 ml of MeCN), followed by 0.1 mmol (1.0 eq) of substrate. After 5 minutes, 2.7 μL (0.02 mmol, 0.2 eq) of 1,1,1-trifluoro-2-butanone were added to the reaction mixture. In order to maximize the product yields, additional portions (from 1 to 3) of oxone (1 eq), $NaHCO_3$ (4 eq) and the ketone (0.2 eq) were then added to the reaction vessel after different times (usually 3 or 6 hours). The reaction was left at T = 0 °C under magnetic stirring for the total reaction time (range from 3 to 48 hours). The mixture was then diluted with a saturated NaCl solution and, after addition of an internal standard (4-*tert*-butylcyclohexanone), extracted with 3x5 mL of CH_2Cl_2, dried over anhydrous Na_2SO_4 and filtered. GC analysis of the solution afforded the conversions of the substrate and product yields relative to the internal standard integration.

S3.2. Results

Product yields for the oxygenation of hydrocarbons **S1-S8** promoted by ETFDO were reported in **Table S1-S8**. The oxygenated products in α to the cyclopropyl group (main products) were identified and quantified by comparison with authentic samples of isolated compounds. Identification of the remote oxygenated products was carried out after chromic acid oxidation of the reaction mixture by means of GC-MS analysis.[6] The spectrometric data of the corresponding ketones matched with those reported in literature (see below). For the oxidation of **S6** and **S7**, quantification of the product yields were obtained after chromic acid oxidation of the reaction mixture.

Table S1. ETFDO-mediated oxidation of bicyclo[3.1.0]hexane (**S1**).[a]

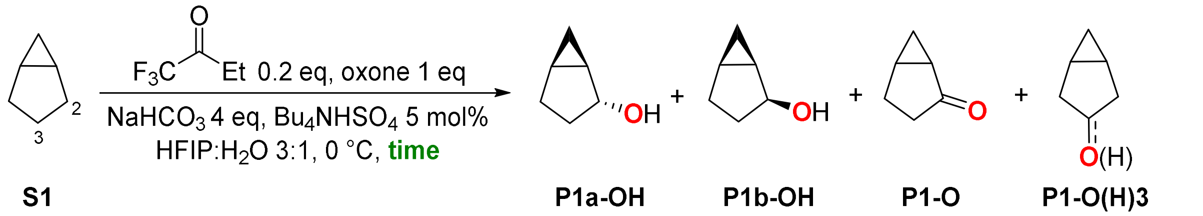

Entry	Time /h	Conv. / %	Product yields (%) P1a-OH	P1b-OH	P1-O	P1-O(H)3	Total yield (%)
1	6[b]	15	8.4	5.5	-	0.5	14.4
2	24[c]	59	27	13	11	1.7	52.7
**3**	**48[d]**	**80**	**32**	**20**	**16**	**2.5**	**70.5**

[a]Conversion and product yields were determined by GC and averaged over two independent experiments. [b]Addition of 1 eq oxone, 4 eq $NaHCO_3$ and 0.2 eq ketone after 3 hours. [c]Addition of 1 eq oxone, 4 eq $NaHCO_3$ and 0.2 eq ketone after 3, 6 and 9 hours. [d]Addition of 1 eq oxone, 4 eq $NaHCO_3$ and 0.2 eq ketone after 6, 9, 24, 30 and 36 hours.

Table S2. ETFDO-mediated oxidation of bicyclo[4.1.0]heptane (**S2**).[a]

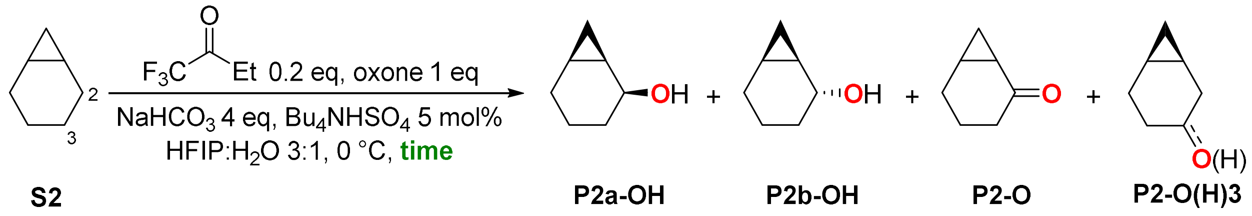

Entry	Time / h	Conv. / %	Product yields (%) P2a-OH	P2b-OH	P2-O	P2-O(H)3	Total yield (%)
1	3	28	22	3.1	-	0.6	25.7
2	6[b]	75	58	6.1	5.1	1.3	70.5
**3**	**9[c]**	**87**	**61**	**4.9**	**14**	**1.6**	**81.5**

[a]Conversion and product yields were determined by GC oxidation and averaged over two independent experiments. [b]Addition of 1 eq oxone, 4 eq $NaHCO_3$ and 0.2 eq ketone after 3 hours. [c]Addition of 1 eq oxone, 4 eq $NaHCO_3$ and 0.2 eq ketone after 3 and 6 hours.

Table S3. ETFDO-mediated oxidation of 1-methylbicyclo[4.1.0]heptane (**S3**).[a]

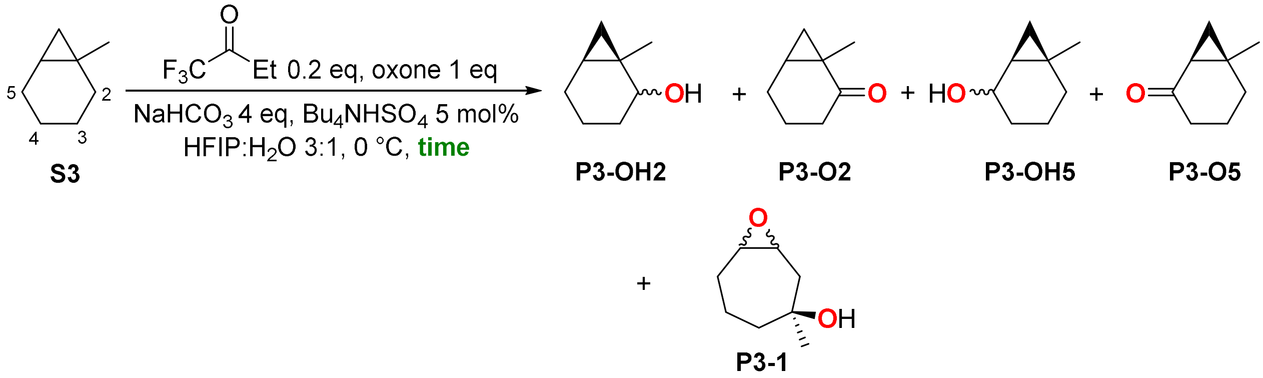

<table>
<tr><th rowspan="2">Entry</th><th rowspan="2">Time / h</th><th rowspan="2">Conv. / %</th><th colspan="5">Product yields (%)</th><th rowspan="2">Ratio P3-1/P3-O(H)5</th><th rowspan="2">Tot. yield / %</th></tr>
<tr><th>P3a-OH2 (P3b-OH2)</th><th>P3-O2</th><th>P3a-OH5 (P3b-OH5)</th><th>P3-O5</th><th>P3-1_{cis} (P3-1_{trans})</th></tr>
<tr><td>1</td><td>3</td><td>28</td><td>10 (1.1)</td><td>-</td><td>9.1 (1.6)</td><td>-</td><td>3.5 (0.6)</td><td>0.38</td><td>25.9</td></tr>
<tr><td>2</td><td>6[b]</td><td>84</td><td>30 (2.4)</td><td>1.4</td><td>25 (4.0)</td><td>3.6</td><td>11 (2.0)</td><td>0.40</td><td>79.4</td></tr>
</table>

[a]Conversion and product yields were determined by GC oxidation and averaged over two independent experiments. **P3a-OH2** and **P3a-OH5** were characterized by *cis* configuration, whereas **P3b-OH2** and **P3b-OH5** by *trans* configuration. [b]Addition of 1 eq oxone, 4 eq $NaHCO_3$ and 0.2 eq ketone after 3 hours. [c]1,1-dibromo-3,3,3-trifluoroacetone is employed as dioxirane precursor.

Table S4. ETFDO-mediated oxidation of bicyclo[5.1.0]octane (**S4**).[a]

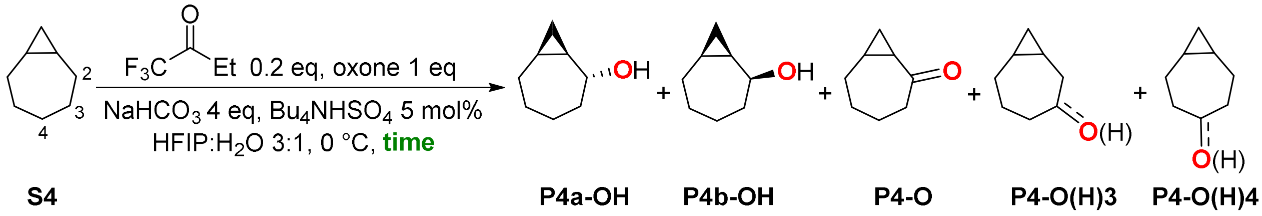

<table>
<tr><th rowspan="2">Entry</th><th rowspan="2">Time / h</th><th rowspan="2">Conv. / %</th><th colspan="5">Product yields (%)</th><th rowspan="2">Total yield (%)</th></tr>
<tr><th>P4a-OH</th><th>P4b-OH</th><th>P4-O</th><th>P4-O(H)3</th><th>P4-O(H)4</th></tr>
<tr><td>1</td><td>3</td><td>25</td><td>16</td><td>1.5</td><td>-</td><td>3.0</td><td>2.8</td><td>23.3</td></tr>
<tr><td>2</td><td>6[b]</td><td>62</td><td>35</td><td>2.5</td><td>3.7</td><td>7.0</td><td>6.0</td><td>54.2</td></tr>
<tr><td>3</td><td>9[d]</td><td>84</td><td>48</td><td>2.8</td><td>8.3</td><td>12</td><td>11</td><td>82.1</td></tr>
</table>

[a]Conversion and product yields were determined by GC oxidation and averaged over two independent experiments. [b]Addition of 1 eq oxone, 4 eq $NaHCO_3$ and 0.2 eq ketone after 3 hours. [d]Addition of 1 eq oxone, 4 eq $NaHCO_3$ and 0.2 eq ketone after 3 and 6 hours.

Table S5. ETFDO-mediated oxidation of bicyclo[6.1.0]nonane (**S5**).[a]

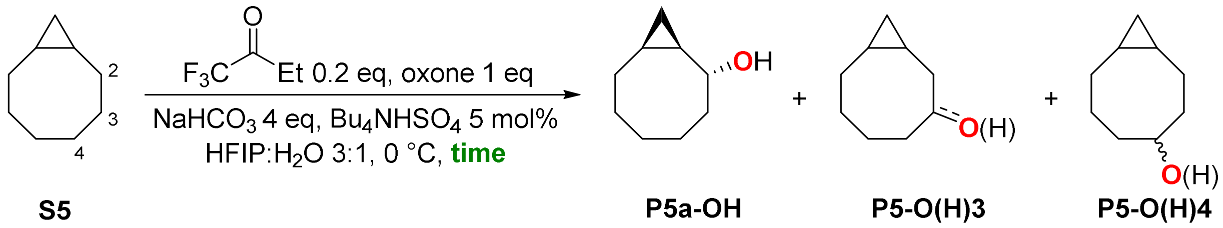

Entry	Time / h	Conv. / %	Product yields (%)			Total yield (%)
			P5a-OH	P5-O(H)3	P5-O(H)4	
**1**	3	26	18	2.2	4.0	24.2
**2**	6[b]	67	49	5.3	11	65.3
**3**	**9[c]**	**86**	**57**	**6**	**13**	**76**

[a]Conversion and product yields were determined by GC and are averaged over two independent experiments. [b]Addition of 1 eq oxone, 4 eq $NaHCO_3$ and 0.2 eq ketone after 3 hours. [c]Addition of 1 eq oxone, 4 eq $NaHCO_3$ and 0.2 eq ketone after 3 and 6 hours.

Table S6. ETFDO-mediated oxidation of 1,1-dimethylcyclohexane (**S6**).[a]

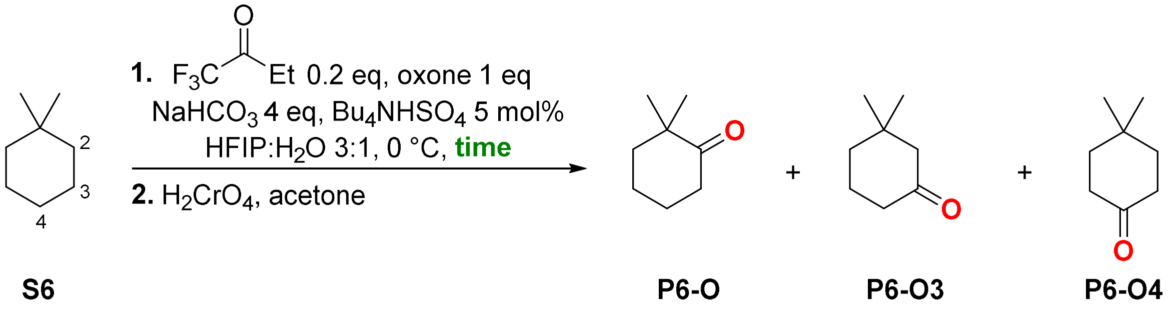

Entry	Time / h	Conv. / %	Product yields (%)			Total yield (%)
			P6-O	P6-O3	P6-O4	
1	3	13	3.0	4.7	2.3	9.7
2	24[b]	29	7.9	12	6.0	25.9
**3**	**48[c]**	**49**	**14**	**21**	**11**	**46**

[a]Conversion and product yields were determined by GC and averaged over two independent experiments. [b]Addition of 1 eq oxone, 4 eq $NaHCO_3$ and 0.2 eq ketone after 8 hours. [c]Addition of 1 eq oxone, 4 eq $NaHCO_3$ and 0.2 eq ketone after 6, 16 and 22 hours.

Table S7. ETFDO-mediated oxidation of spiro[2.5]octane (**S7**).[a]

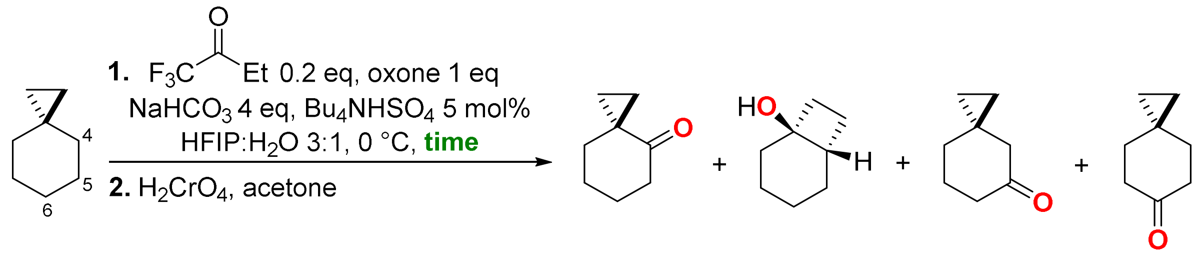

S7 **P7-O** **P7b-OH** **P7-O5** **P7-O6**

Entry	Time / h	Conv. / %	Product yields (%)				Total yield (%)
			P7-O	P7b-OH	P7-O5	P7-O6	
1	3	31	18	1.3	2.5	1.9	23.7
2	24[b]	66	42	3.1	6	4.6	55.7
**3**	**48[c]**	**95**	**66**	**4.8**	**9.2**	**7.0**	**87**

[a]Conversion and product yields were determined by GC after chromic acid oxidation and averaged over two independent experiments. [b]Addition of 1 eq oxone, 4 eq $NaHCO_3$ and 0.2 eq ketone after 8 hours. [c]Addition of 1 eq oxone, 4 eq $NaHCO_3$ and 0.2 eq ketone after 6, 16 and 22 hours.

Table S8. ETFDO-mediated oxidation of 6-*tert*-butylspiro[2.5]octane (**S8**).[a]

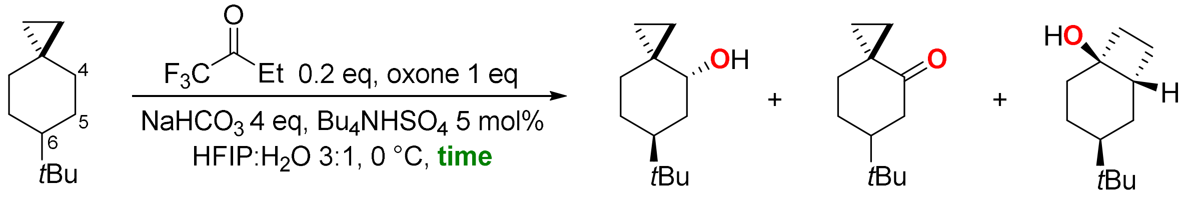

S8 **P8a-OH** **P8-O** **P8b-OH**

Entry	Time (h)	Conv (%)	Product yields (%)			Total yield (%)
			P8a-OH	P8-O	P8b-OH	
**1**	3	9.4	8.8	-	-	8.8
**2**	24[b]	37	30	0.8	2.5	33
**3**	24[c]	73	51	11	4	66
**4**	**48[d]**	**84**	**54**	**20**	**5.5**	**79.5**

[a]Conversion and product yields were determined by GC and averaged over two independent experiments. [b]Addition of 1 eq oxone, 4 eq $NaHCO_3$ and 0.2 eq ketone after 8 hours. [c]Addition of 1 eq oxone, 4 eq $NaHCO_3$ and 0.2 eq ketone after 3, 6 and 9 hours. [d]Addition of 1 eq oxone, 4 eq $NaHCO_3$ and 0.2 eq ketone after 6, 16 and 22 hours.

Additional experiments on the solvent effect on the oxygenation of **S3** and **S8** promoted by ETFDO were reported in **Table S9** and **Table S10**.

Table S9. Solvent effect on ETFDO-mediated oxidation of 1-methylbicyclo[4.1.0]heptane (**S3**).[a]

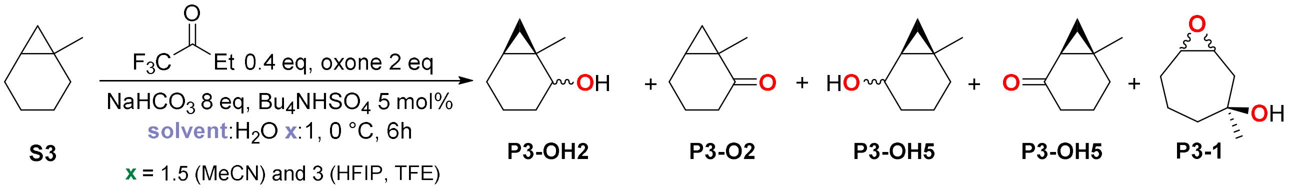

Entry	Solv	Conv. / %	Product yields (%)					Ratio P3-1/ P3-O(H)5	Tot. yield / %
			P3a-OH2 (P3b-OH2)	P3-O2	P3a-OH5 (P3b-OH5)	P3-O5	P3-1_{cis} (P3-1_{trans})		
**1**	HFIP	84	30 (2.4)	1.4	25 (4.0)	3.6	11 (2.0)	0.40	79.4
**2**	TFE	55	20 (1.4)	1.5	16 (2.5)	4.2	3.1 (0.6)	0.16	49.3
**3**	MeCN	20	6.0	2.8	3.2	6.6	-	<0.01	18.6
**4**[b]		56	5.7	16	1.2	25	-	<0.01	47.9

[a]Addition of 1 eq oxone, 4 eq $NaHCO_3$ and 0.2 eq ketone at t = 0 and 3 hours (same reaction conditions of entry 2, **Table S3**). Conversion and product yields were determined by GC oxidation and averaged over two independent experiments. [b]Addition of 1 eq oxone, 4 eq $NaHCO_3$ and 0.2 eq ketone at t = 0, 3, 6 and 9 hours (24 h overall reaction time).

Table S10. Solvent effect on ETFDO-mediated oxidation of 6-*tert*-butylspiro[2.5]octane (**S8**).[a]

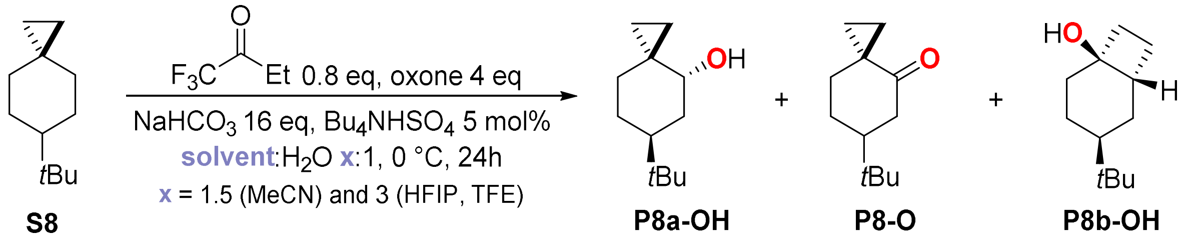

Entry	Solv	Conv./ %	Product yields (%)			Ratio P8b-OH / P8a-OH+P8-O	Total yield (%)
			P8a-OH	P8-O	P8b-OH		
**1**	HFIP	73	51	11	4	0.065	66
**2**	TFE	36	23	9.1	0.9	0.028	33
**3**	MeCN	11	4.8	5.2	-	<0.01	10

[a]Addition of 1 eq oxone, 4 eq $NaHCO_3$ and 0.2 eq ketone at t = 0, 3, 6 and 9 hours (same reaction conditions of entry 3, **Table S8**). Conversion and product yields were determined by GC and averaged over two independent experiments.

S4. Isolation and characterization of the oxidation products

The oxidation products of **S6** were identified by comparison with authentic samples of commercially available compounds. All the other reaction products were isolated by scale-up oxidation of **S1**-**S5**, **S7** and **S8** promoted by ETFDO.

General procedure. The experiments were carried out under magnetic stirring at T = 0 °C in a jacketed 50 ml pyrex glass vessel equipped with an outer refrigerating system. 553 mg (0.9 mmol, 1 eq) of potassium peroxymonosulfate triple salt ($2KHSO_5{\cdot}KHSO_4{\cdot}K_2SO_4$, oxone), 303 mg (3.6 mmol, 4 eq) of $NaHCO_3$ and 9 ml of bidistilled water were charged in the reaction vessel. When the salts were solubilized, 15.3 mg (0.045 mmol, 5 mol%) of Bu_4NHSO_4 and 27 ml of 1,1,1,3,3,3-hexafluoro-2-propanol (HFIP) were then added, followed by the substrate (0.9 mmol, 1 eq). After 5 minutes, 24 μL (0.18 mmol, 0.2 eq) of 1,1,1-trifluoro-2-butanone were added to the reaction mixture. Additional portions of oxone (1 eq), $NaHCO_3$ (4 eq) and the ketone (0.2 eq) were then added to the reaction vessel after different times (usually 3 or 6 hours). The reaction was left at T = 0 °C under magnetic stirring for the total reaction time (between 6 and48 hours. The mixture was then diluted with a saturated NaCl solution, extracted with 3×45 mL portions of CH_2Cl_2, dried over anhydrous Na_2SO_4 and filtered. The solvent was removed at reduced pressure and the resulting residue was purified by flash chromatography on silica gel. The characterization of the purified products was performed by ^{1}H-NMR, ^{13}C-NMR and HRMS or GC/MS.

In the case of the isolation of 1-methylbicyclo[4.1.0]heptane (**S3**) ketone products (**P3-O2** and **P3-O5**), the scale-up ETFDO-mediated oxidation reaction was carried out in MeCN/H_2O 13.5:9 ml (1.5:1).

Cis-bicyclo[3.1.0]hexan-2-ol (**P1b-OH**), *trans*-bicyclo[4.1.0]heptan-2-ol (**P2b-OH**), *cis*- and *trans*-1-methylbicyclo[4.1.0]heptan-2-ol (**P3a-OH2** and **P3b-OH2**), *cis*- and *trans*-6-methylbicyclo[4.1.0]heptan-2-ol (**P3a-OH5** and **P3b-OH5**), *cis*- and *trans*-bicyclo[5.1.0]octan-2-ol (**P4a-OH** and **P4b-OH**) and *cis*-6-*tert*-butylspiro[2.5]octan-4-ol (**P8c-OH**) were isolated after reduction of the parent ketones with $NaBH_4$ according to a reported procedure.[8] The identification of bicyclo[3.1.0]hexan-3-one (**P1-O3**), bicyclo[4.1.0]heptan-3-one (**P2-O3**), 1-methylbicyclo[4.1.0]heptan-2-one (**P3-O2**), 6-methylbicyclo[4.1.0]heptan-2-one (**P3-O5**), bicyclo[5.1.0]octan-3-one (**P4-O3**), bicyclo[5.1.0]octan-4-one (**P4-O4**), bicyclo[6.1.0]nonan-3-one (**P5-O3**), bicyclo[6.1.0]nonan-4-one (**P5-O4**) was performed by means of GC-MS analysis by comparison with reported spectrometric data.

NMR spectra of **P3-1**$_{cis}$ are reported in **Figure S1-S6**.

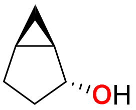

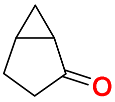

Trans-bicyclo[3.1.0]hexan-2-ol (**P1a-OH**) and bicyclo[3.1.0]hexan-2-one (**P1-O**). Prepared following the general procedure of scale-up ETFDO-mediated oxidation on 0.9 mmol bicyclo[4.1.0]heptane (**S1**) in 48 hours (five additions after 6, 9, 24, 30 and 36 hours). The purification by flash chromatography over silica gel (eluent pentane/Et_2O 3:1) gave 10 mg (0.10 mmol, 11% yield) and 16 mg (0.17 mmol, 19% yield) of colorless liquids identified as *trans*-bicyclo[3.1.0]hexan-2-ol (**P1a-OH**) and bicyclo[3.1.0]hexan-2-one (**P1-O**), respectively. The poor yield values is due to the high volatility of the oxygenated products. Spectroscopic data are consistent with those reported in the literature.[9] **P1a-OH**: ^{1}H-NMR (400 MHz, $CDCl_3$) δ, ppm: 4.22

(d, J = 4.8 Hz, 1H), 1.92 (ddt, J = 16.6, 8.3, 4.2 Hz, 1H), 1.67 (dd, J = 12.5, 8.0 Hz, 1H), 1.58 – 1.49 (m, 2H), 1.47 – 1.30 (m, 3H), 0.43 (td, J = 8.1, 5.3 Hz, 1H), 0.09 – -0.02 (m, 1H). **P1-O**: ^{1}H-NMR (400 MHz, $CDCl_3$) δ, ppm:δ 2.20 – 1.97 (m, 5H), 1.81 – 1.69 (m, 1H), 1.27 – 1.15 (m, 1H), 0.93 (td, J = 4.6, 3.1 Hz, 1H). GC/MS m/z (relative abundance): 96 (M^+, 55), 81, 68 (100), 55, 39.
20 mg of an inseparable mixture of **P1a-OH** and **P1b-OH** were also collected in GC ratio 1:1.

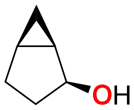
Cis-bicyclo[3.1.0]heptan-2-ol (**P1b-OH**). The crude obtained by the reduction with $NaBH_4$ of 0.15 mmol of bicyclo[3.1.0]hexan-2-one (**P1-O**) was characterized by a diastereomeric ratio **P1b-OH**:**P1a-OH** equal to 5.7:1. The purification by flash chromatography over silica gel (eluent pentane:Et_2O 3:1) gave 4.3 mg (0.044 mmol, 29% yield) of a colorless liquid, identified as *cis*-bicyclo[3.1.0]hexan-2-ol (**P1b-OH**). The poor yield value was due to the high volatility of the oxygenated product. Spectroscopic data are consistent with those reported in the literature.[10] **P1b-OH**: ^{1}H-NMR (400 MHz, $CDCl_3$) δ, ppm: 4.60 – 4.50 (m, 1H), 1.79 – 1.72 (m, 2H), 1.52 – 1.44 (m, 2H), 1.37 – 1.26 (m, 2H), 1.14 – 1.01 (m, 1H), 0.57 (dt, J = 5.0, 3.8 Hz, 1H), 0.37 (td, J = 7.8, 5.1 Hz, 1H).

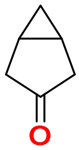
Bicyclo[3.1.0]hexan-3-one (**P1-O3**). Spectrometric data are consistent with those reported in the literature.[11] GC/MS m/z (relative abundance): 96 (M^+, 40), 68 (100), 67, 53, 41.

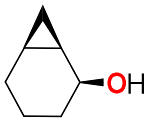
Cis-bicylo[4.1.0]heptan-2-ol (**P2a-OH**). Prepared following the general procedure of scale-up ETFDO-mediated oxidation on 0.9 mmol of bicyclo[4.1.0]heptane (**S2**) in 9 hours (two additions after 3 and 6 hours). The purification by flash chromatography over silica gel (eluent hexane:EtOAc 3:1) gave 21.0 mg (0.19 mmol, 21% yield) of a colorless liquid identified as *cis*-bicylo[4.1.0]heptan-2-ol (**P2a-OH**). The poor yield values were due to the high volatility of the oxygenated products. Spectroscopic data are consistent with those reported in the literature.[12] ^{1}H-NMR (400 MHz, $CDCl_3$) δ, ppm: 4.21 (dt, J = 8.9, 5.8 Hz, 1H), 1.92 – 1.80 (m, 1H), 1.68 – 1.60 (m, 1H), 1.42 (m, J = 14.0, 8.5, 4.3, 2.6 Hz, 3H), 1.31 – 1.24 (m, 1H), 1.23 – 1.13 (m, 2H), 0.99 (m, J = 12.9, 11.4, 8.8, 2.3 Hz, 1H), 0.59 (td, J = 8.8, 4.7 Hz, 1H), 0.32 (q, J = 5.2 Hz, 1H).
19.4 mg of an inseparable mixture of **P2a-OH**, **P2b-OH** and **P2-O** were also collected in GC ratio 9:1:4.2, respectively.

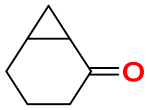
Bicyclo[4.1.0]heptan-2-one (**P2-O**). The crude obtained by the ETFDO-mediated oxidation of 0.9 mmol of bicyclo[4.1.0]heptane (**S2**) in 9 hours (two additions after 3 and 6 hours) was oxidized by chromic acid. The purification by flash chromatography over silica gel (eluent hexane:EtOAc 3:1) gave 44 mg (0.41 mmol, 46% yield) of a colorless liquid identified as

bicyclo[4.1.0]heptan-2-one (**P2-O**). The poor yield value was due to the low volatility of this oxygenated product. Spectroscopic data are consistent with those reported in the literature.[13] ^{1}H-NMR (400 MHz, $CDCl_3$) δ, ppm: 2.36 – 2.27 (m, 1H), 2.15 – 2.04 (m, 1H), 2.01 – 1.87 (m, 2H), 1.80 – 1.72 (m, 2H), 1.71 – 1.61 (m, 2H), 1.24 (q, *J* = 5.3 Hz, 1H), 1.11 (m, *J* = 9.7, 8.2, 5.3 Hz, 1H).

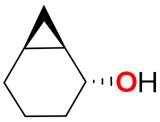

Trans-bicylo[4.1.0]heptan-2-ol (**P2b-OH**). The crude obtained by the reduction with $NaBH_4$ of 0.8 mmol of bicyclo[4.1.0]heptan-2-one (**P2-O**) was characterized by a diastereomeric ratio **P2b-OH**:**P2a-OH** equal to 2:1. The purification by flash chromatography over silica gel (eluent hexane:EtOAc 3:1) gave 41 mg (0.37 mmol, 46% yield) and 13.5 mg (0.12 mmol, 15% yield) of colorless liquids, identified as *trans*-bicylo[4.1.0]heptan-2-ol (**P2b-OH**) and *cis*-bicylo[4.1.0]heptan-2-ol (**P2a-OH**), respectively. Spectroscopic data are consistent with those reported in the literature.[14] **P2b-OH**: ^{1}H-NMR (400 MHz, $CDCl_3$) δ, ppm: 4.05 (dt, *J* = 4.3, 2.8 Hz, 1H), 1.94 – 1.82 (m, 1H), 1.76 (d, *J* = 5.6 Hz, 1H), 1.66 – 1.41 (m, 3H), 1.35 – 1.24 (m, 1H), 1.13 – 0.99 (m, 2H), 0.99 – 0.85 (m, 1H), 0.63 (td, *J* = 9.2, 4.8 Hz, 1H), 0.01 (m, 1H).

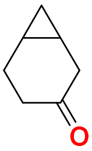

Bicyclo[4.1.0]heptan-3-one (**P2-O3**). Spectrometric data are consistent with those reported in the literature.[15] GC/MS m/z (relative abundance): 110 (M^+, 40), 95, 82 (100), 67, 54 , 39.

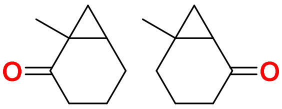

1-methylbicyclo[4.1.0]heptan-2-one (**P3-O2**) and 6-methylbicyclo[4.1.0]heptan-2-one (**P3-O5**). Prepared following the general procedure of scale-up ETFDO-mediated oxidation on 0.9 mmol of 1-methylbicyclo[4.1.0]heptane (**S3**) in MeCN/H_2O 1.5:1 for 48 hours (three additions after 6, 16 and 22 hours). The purification by flash chromatography over silica gel (eluent pentane:Et_2O 4:1) gave 31 mg (0.25 mmol, 28% yield) and 39.3 mg (0.32 mmol, 36% yield) of a colorless liquids identified as 1-methylbicyclo[4.1.0]heptan-2-one (**P3-O2**) and 6-methylbicyclo[4.1.0]heptan-2-one (**P3-O5**), respectively. Spectrometric data are consistent with those reported in literature.[16,17] **P3-O2**: GC/MS m/z (relative abundance): 124 (M^+, 15), 96, 81, 68, 67 (100), 55, 41. **P3-O5**: GC/MS m/z (relative abundance): 124 (M^+, 30), 96, 81, 68 (100), 67 (100), 56, 39.

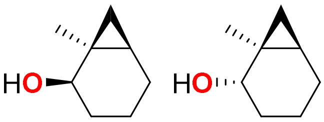

Cis- and *trans*-1-methylbicyclo[4.1.0]heptan-2-ol (**P3a-OH2** and **P3b-OH2**). The crude obtained by the reduction with $NaBH_4$ of 0.37 mmol of 1-methylbicyclo[4.1.0]heptan-2-one (**P3-O2**) was characterized by a diastereomeric ratio **P3a-OH2**/**P3b-OH2** equal to 2.1:1. The purification by flash chromatography over silica gel (eluent hexane:EtOAc 3:1) gave 19 mg (0.15 mmol, 41% yield) and 9.2 mg (0.073 mmol, 20% yield) of

colorless liquids, identified as *cis*-1-methylbicyclo[4.1.0]heptan-2-ol (**P3a-OH2**) and *trans*-1-methylbicyclo[4.1.0]heptan-2-ol (**P3b-OH2**), respectively. Spectroscopic data are consistent with those reported in the literature.[18] **P3a-OH2**: ^{1}H-NMR (400 MHz, $CDCl_3$) δ, ppm: 3.85 (dd, *J* = 9.5, 5.7 Hz, 1H), 1.94 – 1.81 (m, 1H), 1.68 (m, *J* = 12.7, 5.7, 3.9, 1.7 Hz, 1H), 1.36 (m, *J* = 21.8, 15.7, 5.6, 2.3 Hz, 3H), 1.16 (m, 4H), 0.98 – 0.82 (m, 2H), 0.41 – 0.30 (m, 2H). **P3b-OH2**: ^{1}H-NMR (400 MHz, $CDCl_3$) δ, ppm: 4.06 (t, *J* = 3.4 Hz, 1H), 2.08 – 1.92 (m, 1H), 1.63 – 1.37 (m, 5H), 1.20 (dq, *J* = 7.9, 3.4 Hz, 1H), 1.12 (s, 3H), 0.91 – 0.81 (m, 1H), 0.39 (dd, *J* = 8.9, 4.5 Hz, 1H), 0.16 (t, *J* = 4.9 Hz, 1H).

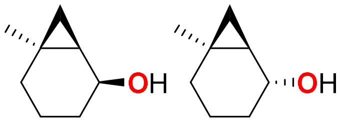

Cis- and *trans*-6-methylbicyclo[4.1.0]heptan-2-ol (**P3a-OH5** and **P3b-OH5**). The crude obtained by the reduction with $NaBH_4$ of 0.48 mmol of 6-methylbicyclo[4.1.0]heptan-2-one (**P3-O5**) was characterized by a diastereomeric ratio **P3a-OH5**/**P3b-OH5** equal to 1:3.2. The purification by flash chromatography over silica gel (eluent hexane:EtOAc 3:1) gave 8.9 mg (0.071 mmol, 15% yield) and 26 mg (0.21 mmol, 44% yield) of colorless liquids, identified as *cis*-6-methylbicyclo[4.1.0]heptan-2-ol (**P3a-OH5**) and *trans*-6-methylbicyclo[4.1.0]heptan-2-ol (**P3b-OH5**), respectively. Spectroscopic data are consistent with those reported in the literature.[18,19] **P3a-OH5**: ^{1}H-NMR (400 MHz, $CDCl_3$) δ, ppm: 4.22 (q, *J* = 6.3 Hz, 1H), 1.63 – 1.49 (m, 3H), 1.41 – 1.30 (m, 2H), 1.27 – 1.21 (m, 1H), 1.09 (m, 5H), 0.50 (t, *J* = 4.9 Hz, 1H), 0.31 (dd, *J* = 9.0, 4.5 Hz, 1H). **P3b-OH5**: ^{1}H-NMR (400 MHz, $CDCl_3$) δ, ppm: 3.94 (m, *J* = 7.3, 4.8, 1.2 Hz, 1H), 1.87 (s, 1H), 1.70 (dt, *J* = 12.7, 5.6 Hz, 1H), 1.63 – 1.45 (m, 3H), 1.20 (m, *J* = 12.1, 9.2, 6.9, 2.4 Hz, 1H), 1.08 (s, 4H), 0.75 (m, *J* = 9.7, 5.4, 1.2 Hz, 1H), 0.37 (m, *J* = 9.6, 4.7, 0.8 Hz, 1H), 0.14 (t, *J* = 5.0 Hz, 1H).

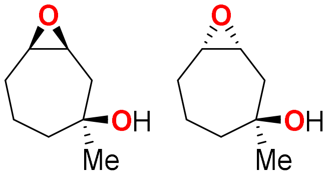

Cis- and *trans*-3-methyl-8-oxabicyclo[5.1.0]octan-3-ol (**P3-1**$_{cis}$ and **P3-1**$_{trans}$). Prepared following the general procedure of scale-up ETFDO-mediated oxidation on 0.9 mmol of 1-methylbicyclo[4.1.0]heptane (**S3**) in 6 hours (one addition after 3 hours). The purification by flash chromatography over silica gel (eluent hexane/EtOAc 3:1) gave 14 mg (11% yield) of a colorless liquid identified as *cis*-3-methyl-8-oxabicyclo[5.1.0]octan-3-ol (**P3-1**$_{cis}$). The identification of *trans*-3-methyl-8-oxabicyclo[5.1.0]octan-3-ol (**P3-1**$_{trans}$) was performed by means of GC-MS analysis of the reaction mixture, because of the low amount formed during in che oxidation of **S3**. **P3-1**$_{cis}$: ^{1}H-NMR (700 MHz, $CDCl_3$) δ, ppm: δ 3.85 (s, 1H), 3.29 – 3.25 (m, 1H), 3.20 – 3.16 (m, 1H), 2.38 (m, *J* = 15.3, 6.0, 2.2 Hz, 1H), 2.33 – 2.26 (m, 1H), 1.98 – 1.93 (m, 1H), 1.84 (m, *J* = 13.5, 12.5, 3.1, 1.5 Hz, 1H), 1.73 (m, *J* = 14.1, 6.0, 2.2, 1.7 Hz, 1H), 1.62 (m, *J* = 14.9, 12.5, 2.4 Hz, 1H), 1.51 (m, *J* = 14.7, 5.8, 2.7 Hz, 1H), 1.24 (m, 4H). ^{13}C{^{1}H}-NMR (700 MHz, $CDCl_3$) δ, ppm: 71.7, 57.5, 55.5, 43.1, 39.0, 31.4, 27.8, 17.8. GC/MS m/z (relative abundance): 142 (M^+, 100), 128, 112, 100, 84, 58, 41. **P3-1**$_{trans}$: GC/MS m/z (relative abundance): 142 (M^+, 15), 128, 114, 99, 85, 71, 58, 43 (100), 41, 30.

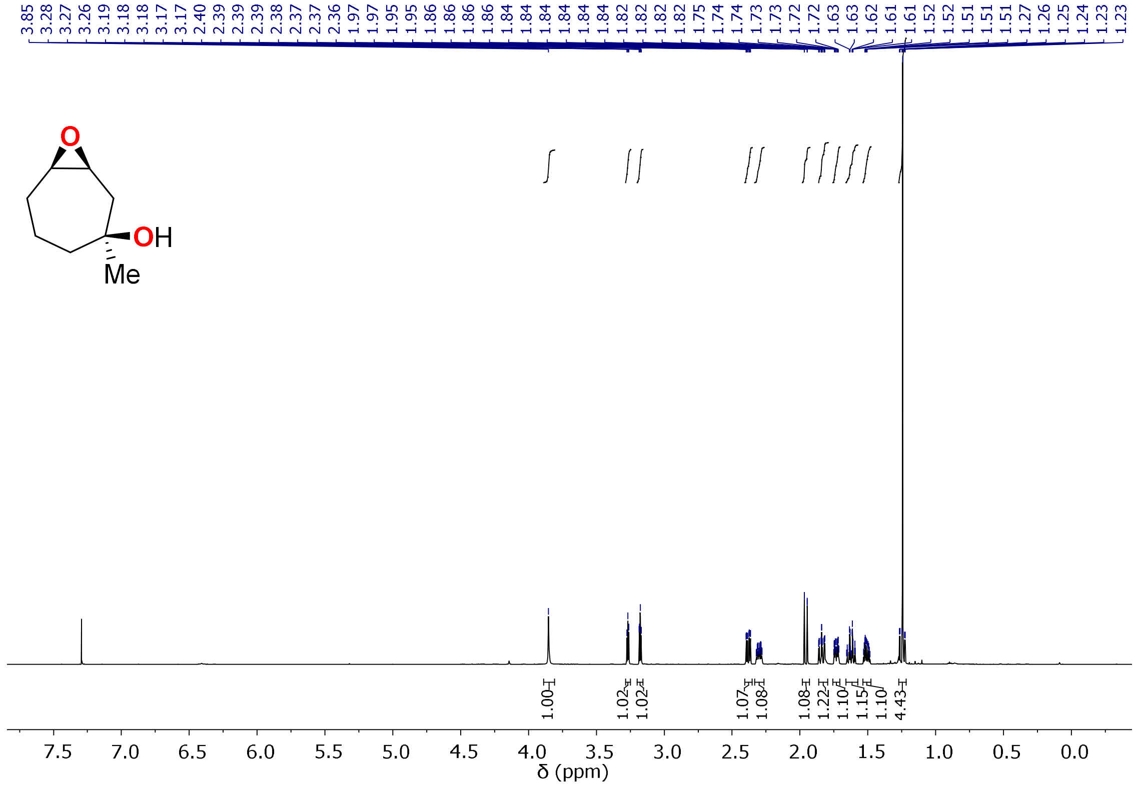

Figure S1. ^{1}H-NMR (700 MHz, $CDCl_3$) spectrum of *cis*-3-methyl-8-oxabicyclo[5.1.0]octan-3-ol (**P3-1**$_{cis}$).

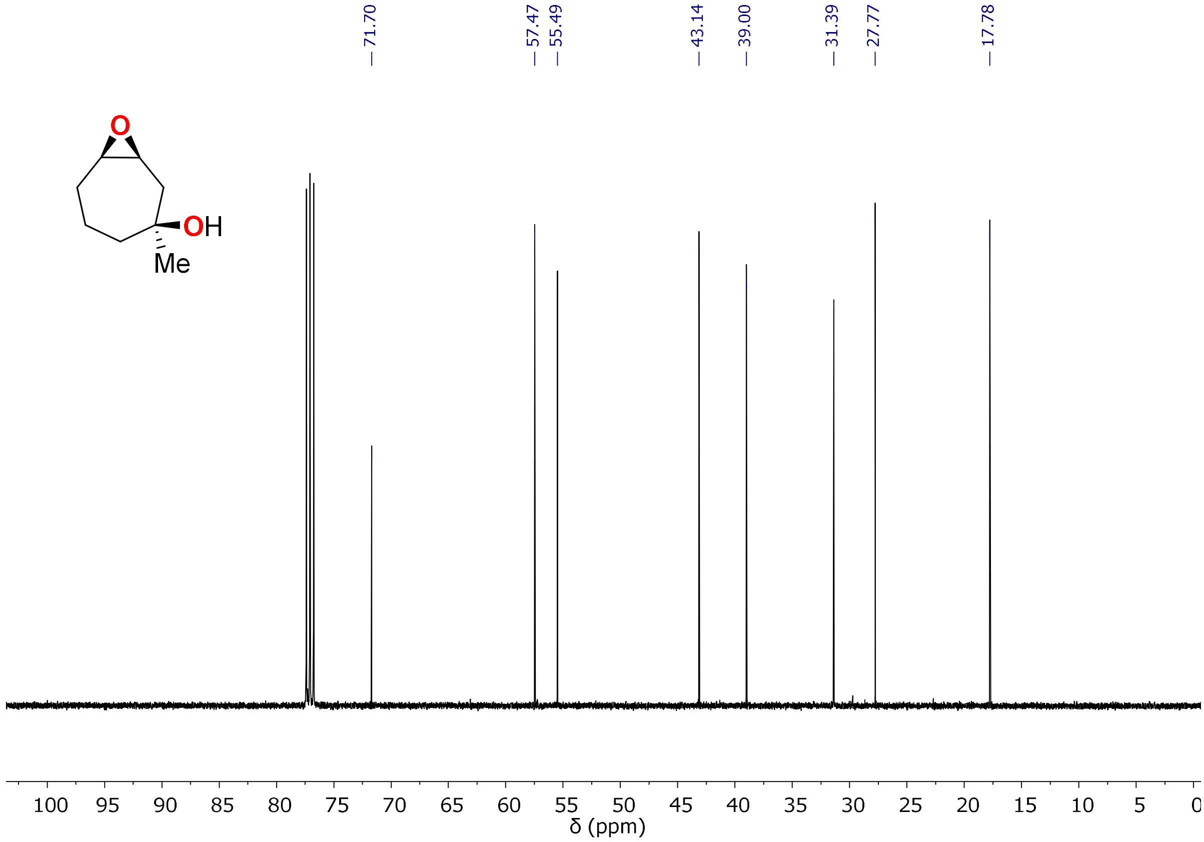

Figure S2. $^{13}C\{^{1}H\}$-NMR (700 MHz, $CDCl_3$) spectrum of *cis*-3-methyl-8-oxabicyclo[5.1.0]octan-3-ol (**P3-1***cis*).

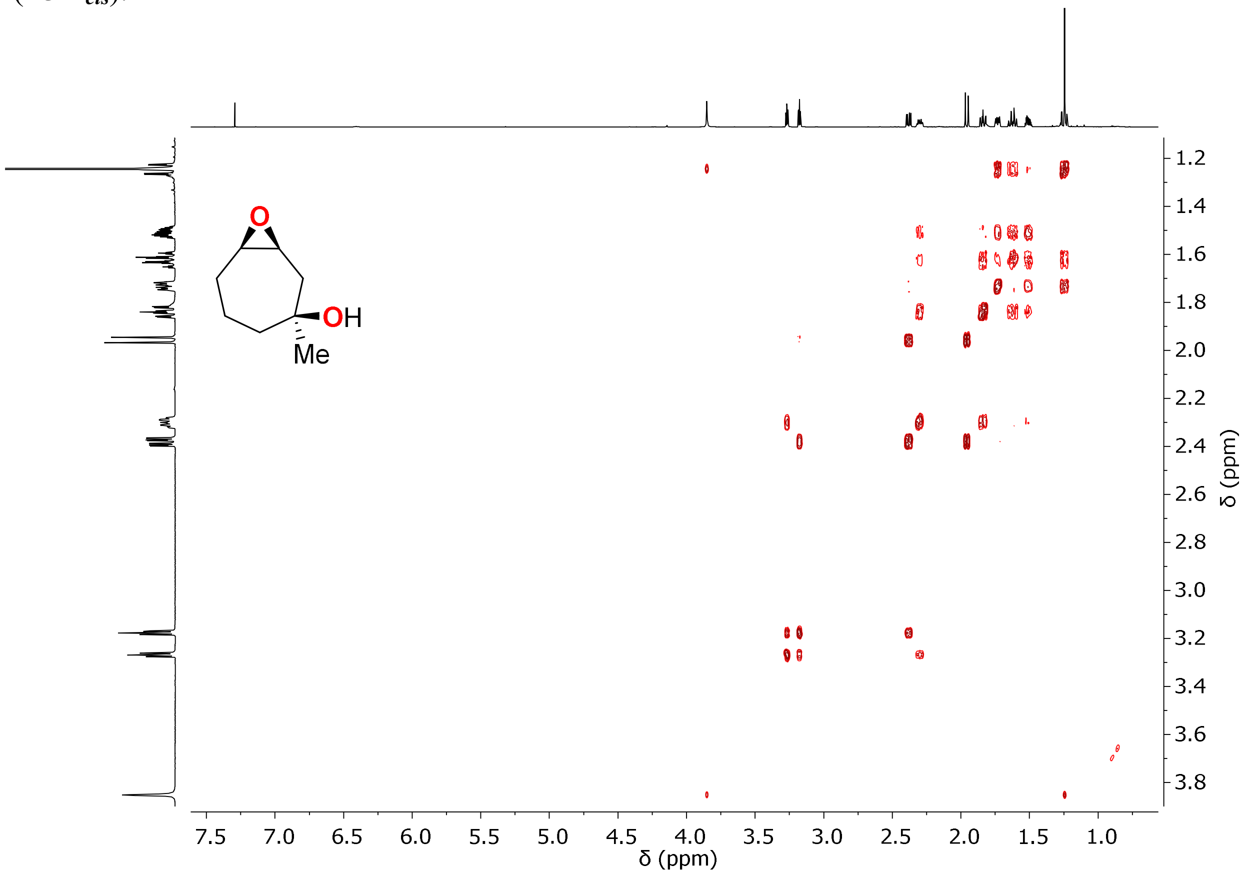

Figure S3. COSY-NMR (700 MHz, $CDCl_3$) spectrum of *cis*-3-methyl-8-oxabicyclo[5.1.0]octan-3-ol (**P3-1***cis*).

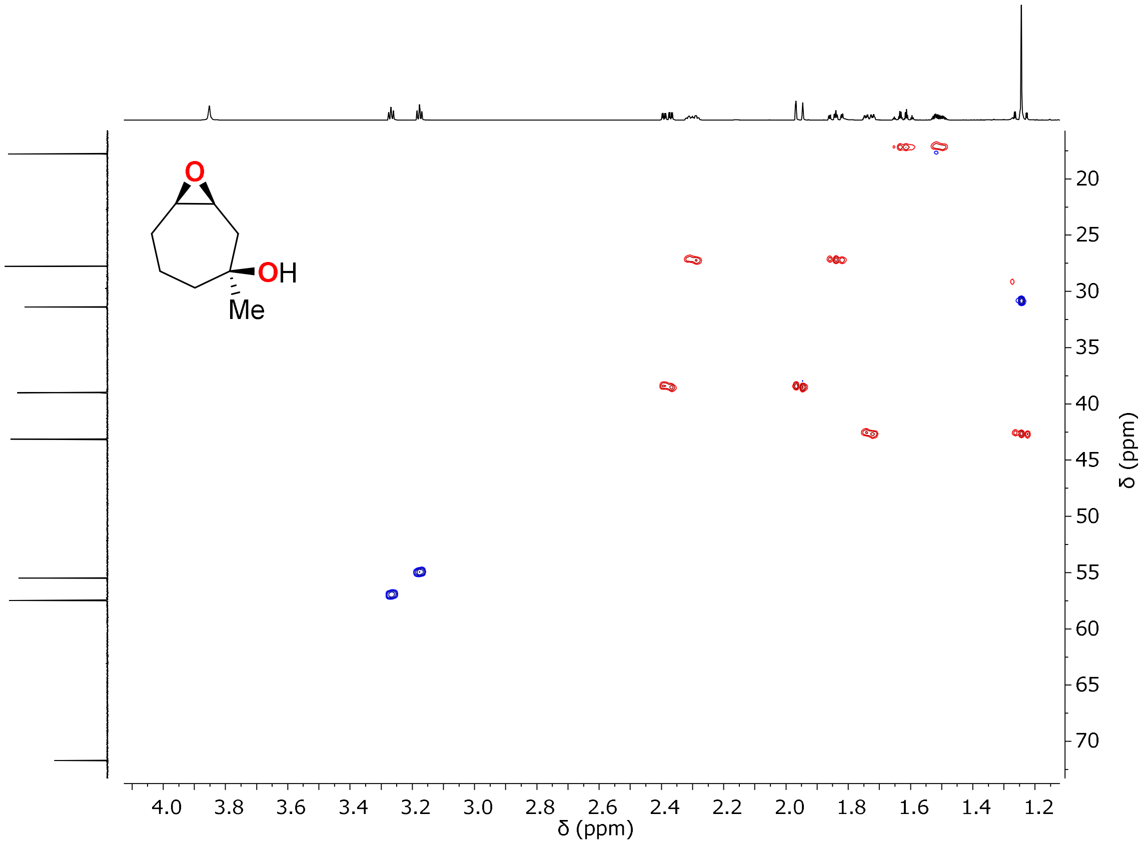

Figure S4. HSQC-NMR (700 MHz, $CDCl_3$) spectrum of *cis*-3-methyl-8-oxabicyclo[5.1.0]octan-3-ol (**P3-1*cis***).

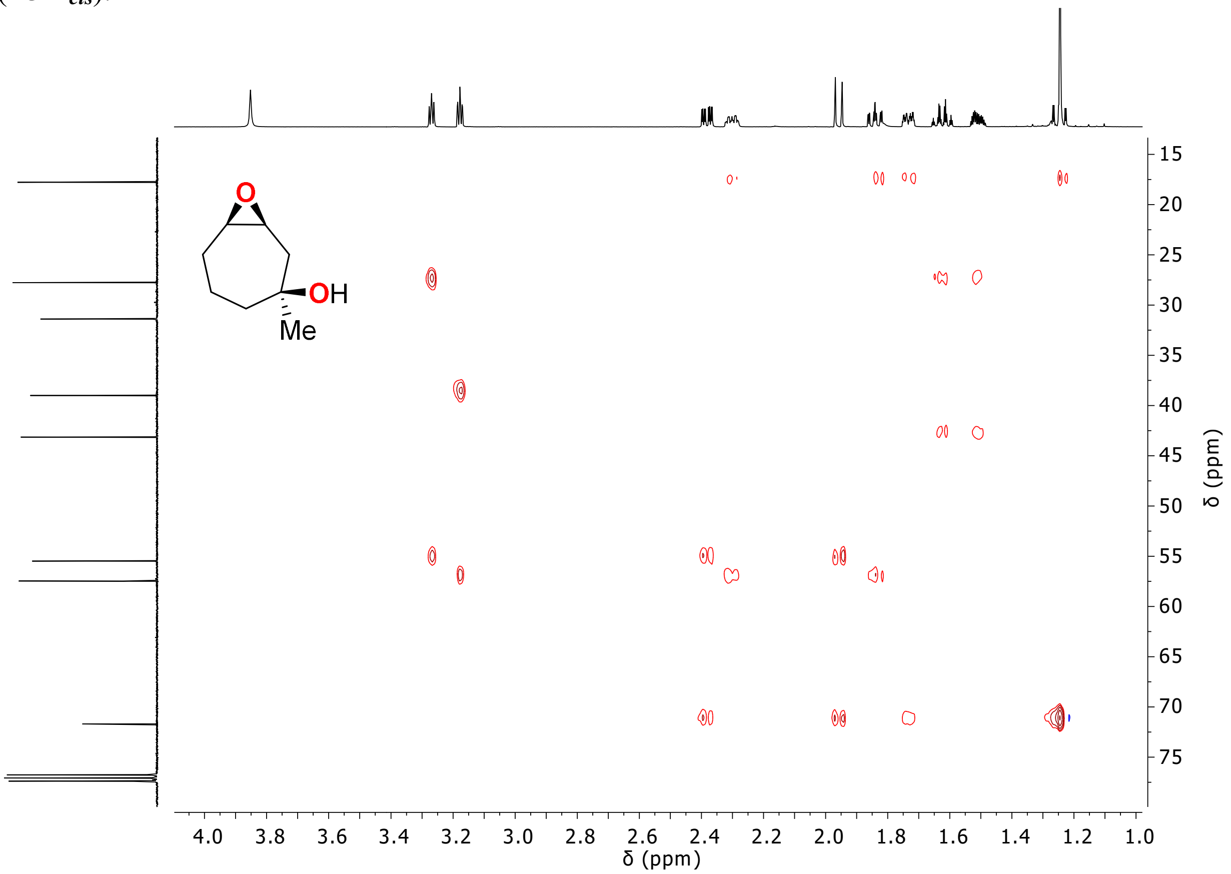

Figure S5. 1,1-Adequate-NMR (700 MHz, $CDCl_3$) spectrum of *cis*-3-methyl-8-oxabicyclo[5.1.0]octan-3-ol (**P3-1*cis***).

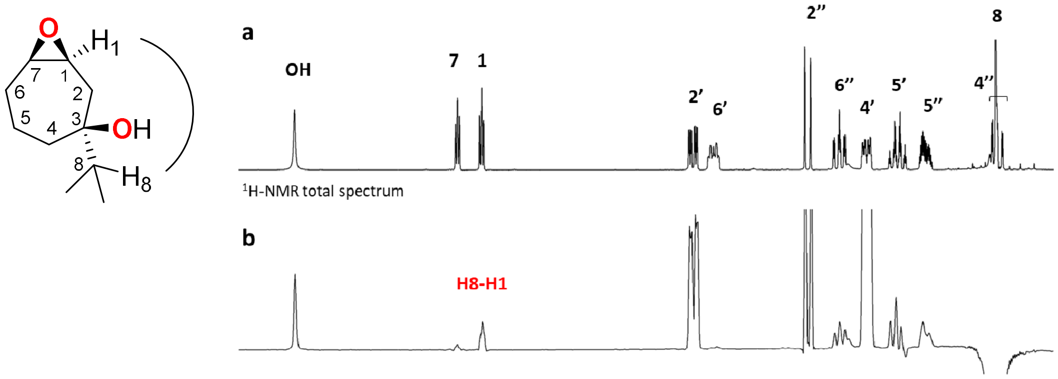

Figure S6. a) ^{1}H-NMR (700 MHz, $CDCl_3$) and **b)** selective NOE (700 MHz, $CDCl_3$, δ_{irr} = 1.24 ppm) spectra of *cis*-3-methyl-8-oxabicyclo[5.1.0]octan-3-ol (**P3-1*cis***).

The correlation between H_1 and H_8 in selective NOE (irradiation of H_8, δ_{irr} = 1.24 ppm) indicates that the configuration of the molecule is *cis*.

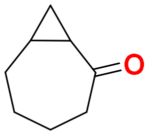

Bicyclo[5.1.0]octan-2-one (**P4-O**). The crude obtained by scale-up ETFDO-mediated oxidation of 1.8 mmol of bicyclo[5.1.0]heptane (**S4**) in 9 hours (two additions after 3 and 6 hours) was oxidized by chromic acid. The purification by flash chromatography over silica gel (eluent hexane:EtOAc 10:1) gave 90 mg (0.72 mmol, 40% yield) of a colorless liquid identified as bicyclo[5.1.0]octan-2-one (**P4-O**). Spectrometric data are consistent with those reported in the literature.[19] GC/MS m/z (relative abundance): 124 (M+, 40), 95, 80, 67, 55 (100), 39.

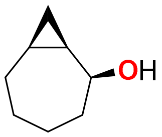 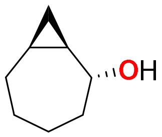

Cis-bicyclo[5.1.0]octan-2-ol (**P4a-OH**) and *trans*-bicyclo[5.1.0]octan-2-ol (**P4b-OH**). The crude obtained by the reduction with $NaBH_4$ of 0.52 mmol of bicyclo[5.1.0]octan-2-one (**P4-O**) wass characterized by a diastereomeric ratio **P4a-OH**:**P4b-OH** equal to 1.5:1. The purification by flash chromatography over silica gel (eluent hexane:EtOAc 3:1) gave 16 mg (0.13 mmol, 25% yield) and 11 mg (0.087 mmol, 17% yield) of colorless liquids, identified as *cis*-bicyclo[5.1.0]octan-2-ol (**P4a-OH**) and *trans*-bicyclo[5.1.0]octan-2-ol (**P4b-OH**), respectively. Spectroscopic data are consistent with those reported in the literature.[20] **P4a-OH**: ^{1}H-NMR (400 MHz, $CDCl_3$) δ, ppm: 4.20 (dt, *J* = 9.8, 3.3 Hz, 1H), 1.86 – 1.69 (m, 3H), 1.63 – 1.39 (m, 4H), 1.33 – 1.20 (m, 1H), 1.06 (m, *J* = 44.4, 15.4, 9.5, 5.9, 3.6 Hz, 3H), 0.55 (q, *J* = 5.6 Hz, 1H), 0.41 (td, *J* = 8.7, 4.9 Hz, 1H). **P4b-OH**: ^{1}H-NMR (400 MHz, $CDCl_3$) δ, ppm: 3.44 – 3.25 (m, 1H), 2.20 (dt, *J* = 12.9, 5.4 Hz, 1H), 1.90 – 1.74 (m, 3H), 1.67 (dd, *J* = 9.7, 5.0 Hz, 2H), 1.36 – 1.19 (m, 2H), 0.96 – 0.83 (m, 3H), 0.81 – 0.70 (m, 1H), 0.29 (q, *J* = 4.6, 4.1 Hz, 1H).

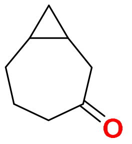 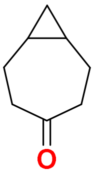

$10\text{-}2_k$ **$10\text{-}3_k$** Bicyclo[5.1.0]octan-3-one (**P4-O3**) and bicyclo[5.1.0]octan-4-one (**P4-O4**). Spectrometric data are consistent with those reported in the literature.[21] **P4-O3**: GC-MS m/z (relative abundance): 124 (M^+, 30), 96, 81, 67 (100), 55, 39. **P4-O4**: GC-MS m/z (relative abundance): 124 (M^+, 50), 109, 96, 83 (100), 67, 55, 39.

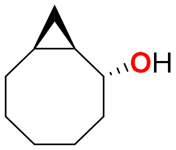

Trans-bicyclo[6.1.0]nonan-2-ol (**P5a-OH**). Prepared following the general procedure of scale-up ETFDO-mediated oxidation on 0.9 mmol of bicyclo[6.1.0]nonane (**S5**) in 9 hours (two additions after 3 and 6 hours). The purification by flash chromatography over silica gel (eluent hexane:EtOAc 3:1) gave 30 mg (0.21 mmol, 23% yield) of a white solid identified as *trans*-bicyclo[6.1.0]nonan-2-ol (**P5a-OH**). Spectroscopic data are consistent with those reported in the

literature.[22] ^{1}H-NMR (400 MHz, $CDCl_3$) δ, ppm: 3.26 (m, J = 10.5, 9.0, 4.0 Hz, 1H), 2.10 – 2.03 (m, 1H), 1.87 (m, J = 11.5, 8.1, 3.1 Hz, 1H), 1.74 – 1.62 (m, 3H), 1.58 – 1.46 (m, 4H), 1.43 – 1.31 (m, 2H), 0.83 (m, J = 18.9, 8.4, 4.1, 2.3 Hz, 2H), 0.74 (dd, J = 6.4, 2.8 Hz, 1H), 0.10 (q, J = 4.4 Hz, 1H).

58 mg of an inseparable mixture of **P5a-OH**, **P5a-O(H)3** and **P5a-O(H)4** were also collected in GC ratio 4.6:1:2.6, respectively.

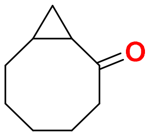

Bicyclo[6.1.0]nonan-2-one (**P5-O**). Prepared following the chromic acid oxidation of 0.17 mmol of *trans*-bicyclo[6.1.0]nonan-2-ol (**P5a-OH**). 15.5 mg (0.11 mmol, 65% yield) of a colorless liquid identified as bicyclo[6.1.0]nonan-2-one (**P5-O**) were collected without any purification. Spectrometric data are consistent with those reported in the literature.[22] GC/MS m/z (relative abundance): 138 (M^+, 6), 110, 95, 81, 67, 55 (100), 41.

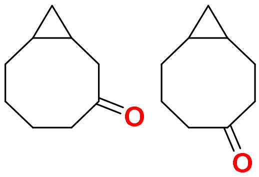

Bicyclo[6.1.0]nonan-3-one (**P5-O3**) and bicyclo[6.1.0]nonan-4-one (**P5-O4**). Spectrometric data are consistent with those reported in the literature.[23] **P5-O3**: GC/MS m/z (relative abundance): 138 (M^+, 22), 123, 109, 94, 79, 67, 55, 41 (100). **P5-O4**: GC/MS m/z (relative abundance): 138 (M^+, 35), 123, 97 (100), 79, 67, 55, 41.

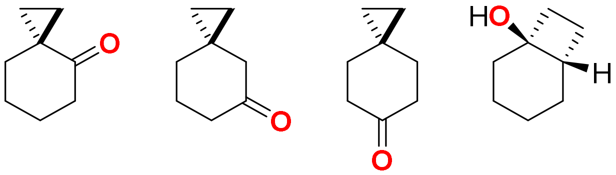

Spiro[2.5]octan-4-one (**P7-O**), spiro[2.5]octan-5-one (**P7-O5**), spiro[2.5]octan-6-one (**P7-O6**) and bicyclo[4.2.0]octan-1-ol (**P7b-OH**). The crude obtained by scale-up ETFDO-mediated oxidation of 0.9 mmol of spiro[2.5]octane (**S7**) in 48 hours (three additions after 6, 16 and 22 hours) was oxidized by chromic acid. The purification by flash chromatography over silica gel (eluent hexane/EtOAc 10:1) gave 35.4 mg (0.29 mmol, 32% yield), 8.6 mg (0.069 mmol, 7.7% yield) and 3.7 mg (0.030 mmol, 3.3% yield) of colorless liquids identified as spiro[2.5]octan-4-one (**P7-O**), spiro[2.5]octan-5-one (**P7-O5**) and spiro[2.5]octan-6-one (**P7-O6**), respectively. By changing the polarity of the eluent (hexane/EtOAc 3:1), 3.4 mg (0.027 mmol, 3% yield) of a white solid identified as bicyclo[4.2.0]octan-1-ol (**P7b-OH**) were also collected. The poor yield values are due to the high volatility of the oxygenated products. Spectroscopic data are consistent with those reported in the literature.[1,24,25] **P7-O**: ^{1}H-NMR (400 MHz, $CDCl_3$) δ, ppm: 2.42 (t, J = 6.7 Hz, 2H), 1.84 (m, 2H), 1.83 – 1.75 (m, 2H), 1.65 (dd, J = 5.6, 5.9 Hz, 2H), 1.10 (dd, J = 3.5, 5.9 Hz, 2H), 0.47 (dd, J = 3.5, 5.9 Hz, 2H). **P7-O5**: ^{1}H-NMR (400 MHz, $CDCl_3$) δ, ppm: 2.36 (t, J = 6.7 Hz, 2H), 2.14 (s, 2H), 1.93 (m, 2H), 1.54 (m, 2H), 0.34 (m, 4H). **P7b-OH**: ^{1}H-NMR (400 MHz, $CDCl_3$) δ, ppm: δ 2.27 (dt, J = 15.3, 7.9 Hz, 1H), 1.89 – 1.70 (m, 3H), 1.57 (qq, J = 13.9, 8.2, 7.7 Hz, 6H), 1.35 (m, J = 26.7, 11.2, 7.1 Hz, 4H). ^{13}C-NMR (400

MHz, $CDCl_3$) δ, ppm: 72.1, 43.7, 36.2, 35.4, 24.2, 21.6, 21.2, 15.8. GC-MS m/z (relative abundance): 126 (M^+, 5), 112, 98 (100), 97, 70, 58, 41, 30.

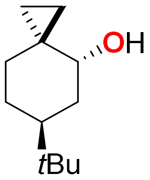

Trans-6-(*tert*-butyl)spiro[2.5]octan-4-ol (**P8a-OH**). Prepared following the general procedure of scale-up ETFDO-mediated oxidation on 0.9 mmol of 6-*tert*-butylspiro[2.5]octane (**S8**) in 48 hours (three additions after 6, 16 and 22 hours). The purification by flash chromatography over silica gel (eluent hexane:EtOAc 3:1) gave 37 mg (0.020 mmol, 22% yield) of a white solid identified as *trans*-6-(*tert*-butyl)spiro[2.5]octan-4-ol (**P8a-OH**). Spectroscopic data are consistent with those reported in literature.[1,26] ^{1}H-NMR (400 MHz, $CDCl_3$) δ, ppm: 3.09 (s, 1H), 2.16 – 2.06 (m, 1H), 1.91 (m, *J* = 13.3, 3.0 Hz, 1H), 1.75 (m, *J* = 12.2, 6.4, 3.4 Hz, 1H), 1.59 – 1.46 (m, 2H), 1.35 (td, *J* = 13.1, 2.8 Hz, 1H), 1.15 (m, *J* = 12.5, 3.7 Hz, 1H), 0.89 (s, 9H), 0.80 – 0.72 (m, 1H), 0.47 – 0.41 (m, 1H), 0.36 – 0.27 (m, 3H).

58 mg of an inseparable mixture of **P8a-OH** and **P8b-OH** in GC ratio 4:1 were also collected.

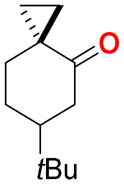

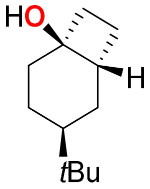

6-(*tert*-butyl)spiro[2.5]octan-4-one (**P8-O**) and *cis*-4-(*tert*-butyl)bicyclo[4.2.0]octan-1-ol (**P8b-OH**). The crude obtained by the ETFDO-mediated oxidation of 0.9 mmol of 6-(*tert*-butyl)spiro[2.5]octane (**S8**) in 48 hours (three additions after 6, 16 and 22 hours) was oxidized by chromic acid. The purification by flash chromatography over silica gel (eluent hexane:EtOAc 10:1) gave 107.8 mg (0.60 mmol, 67% yield) of a colorless liquid and 8.1 mg (0.045 mmol, 5.0% yield) of a white solid identified as 6-(*tert*-butyl)spiro[2.5]octan-4-one (**P8-O**) and 4-(*tert*-butyl)bicyclo[4.2.0]octan-1-ol (**P8b-OH**), respectively. Spectroscopic data are consistent with those reported in literature.[1,26] **P8-O**: ^{1}H-NMR (400 MHz, $CDCl_3$) δ, ppm: 2.54 (m, *J* = 16.4, 4.5, 2.2 Hz, 1H), 2.14 (dd, *J* = 16.4, 12.7 Hz, 1H), 2.04 – 1.89 (m, 2H), 1.72 – 1.63 (m, 1H), 1.56 (m, *J* = 13.8, 7.0, 3.7 Hz, 1H), 1.51 – 1.42 (m, 2H), 0.92 (m, 10H), 0.69 (ddd, *J* = 9.5, 6.5, 3.1 Hz, 1H), 0.58 (m, *J* = 9.0, 6.6, 3.7 Hz, 1H). **P8b-OH**: ^{1}H-NMR (400 MHz, $CDCl_3$) δ, ppm: 2.38 (q, *J* = 9.3 Hz, 1H), 1.92 – 1.67 (m, 4H), 1.62 – 1.54 (m, 3H), 1.50 (m, *J* = 13.8, 3.8, 1.8 Hz, 1H), 1.37 – 1.28 (m, 2H), 1.17 (m, *J* = 12.3, 3.9, 2.1 Hz, 1H), 1.04 (m, *J* = 13.4, 12.6, 11.6, 3.0 Hz, 1H), 0.87 (s, 9H).

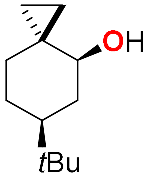

Cis-6-(tert-butyl)spiro[2.5]octan-4-ol (**P8c-OH**). The crude obtained by the reduction with $NaBH_4$ of 0.76 mmol of 6-(*tert*-butyl)spiro[2.5]octan-4-one (**P8-O**) was characterized by a diastereomeric ratio **P8c-OH**/**P8a-OH** equal to 66:1. The purification by flash chromatography over silica gel (eluent hexane:EtOAc 3:1) gave 110.8 mg (0.61 mmol, 80% yield) of a white solid, identified as *cis*-6-(tert-butyl)spiro[2.5]octan-4-ol (**P8c-OH**). Spectroscopic data are consistent with

those reported in literature.[1,26] ^{1}H-NMR (400 MHz, $CDCl_3$) δ, ppm: 3.76 (dd, *J* = 10.8, 3.9 Hz, 1H), 2.01 (m, *J* = 11.5, 4.5, 2.5 Hz, 1H), 1.75 (m, *J* = 13.1, 3.5, 1.8 Hz, 1H), 1.66 (dp, *J* = 11.4, 2.6 Hz, 1H), 1.22 (tt, *J* = 12.0, 2.8 Hz, 1H), 1.15 – 0.99 (m, 4H), 0.90 (s, 9H), 0.69 (m, *J* = 9.2, 5.2, 4.0 Hz, 1H), 0.62 – 0.55 (m, 1H), 0.22 – 0.16 (m, 1H), 0.11 – 0.08 (m, 1H).

S5. Computational Section

Recent studies showed (u)ωB97X-D/6-311++G(d,p) is apt for evaluating reactivity and selectivity in aliphatic C–H bond oxygenation and hydrogen abstraction by alkoxy radicals.[6,27] Before extensive computational studies, preliminary studies with (u)ωB97X-D/6-311++G(d,p) resulted in qualitatively good results matching the experimental studies. Thus, (u)ωB97X-D/6-311++G(d,p) is employed in this work.

S5.1 Calculated Energetics

Table S11. Energies of the optimized structures ((u)ωB97X-D/6-311++G(d,p)/SMD = water/temperature = 273.15)

Structure	E	ZPE	H	T.S	T.qh-S	G(T)	qh-G(T)
DIOXIRANE	-605.30521	0.09417	-605.202564	0.039375	0.038492	-605.241939	-605.241056
P7-4-OH	-918.691376	0.300141	-918.374422	0.059605	0.056446	-918.434028	-918.430868
P7-5-OH	-918.691175	0.300541	-918.374102	0.058662	0.055656	-918.432763	-918.429757
P7-6-OH	-918.69091	0.300406	-918.373839	0.059578	0.056239	-918.433417	-918.430078
P7b-OH	-918.692893	0.301308	-918.375213	0.058385	0.055434	-918.433598	-918.430647
P8a-OH	-1075.945031	0.413034	-1075.510833	0.069647	0.065037	-1075.58048	-1075.57587
P8b-OH	-1075.944753	0.413719	-1075.510171	0.06854	0.064313	-1075.57871	-1075.574483
P8c-OH	-1075.944116	0.412903	-1075.510019	0.068828	0.064688	-1075.578848	-1075.574707
S1-TS1C2cis	-839.904177	0.237246	-839.653095	0.052354	0.050358	-839.705449	-839.703453
S1-TS1C2trans	-839.905036	0.237207	-839.654049	0.052022	0.050088	-839.706072	-839.704137
S1-TS1C3cis	-839.903186	0.236809	-839.652645	0.051789	0.049991	-839.704435	-839.702637
S1-TS1C3trans	-839.899765	0.236834	-839.64915	0.052241	0.050157	-839.70139	-839.699307
S1	-234.620116	0.146562	-234.468325	0.030524	0.030531	-234.498849	-234.498856
S2-TS1C2cis	-879.221854	0.266813	-878.940383	0.054218	0.05196	-878.9946	-878.992343
S2-TS1C2trans	-879.221252	0.266919	-878.939701	0.053836	0.051789	-878.993538	-878.99149
S2-TS1C3cis	-879.21725	0.266549	-878.936043	0.054347	0.052053	-878.990391	-878.988096
S2-TS1C3trans	-879.220766	0.266598	-878.939631	0.053338	0.051466	-878.992968	-878.991096
S2	-273.934721	0.176065	-273.752597	0.032624	0.032621	-273.78522	-273.785218
S3-Int1	-918.644498	0.297835	-918.329576	0.059642	0.056574	-918.389218	-918.38615
S3-Int2	-918.692923	0.299188	-918.376583	0.060453	0.057082	-918.437035	-918.433665
S3-TS1C2cis	-918.537575	0.294267	-918.227164	0.057718	0.054942	-918.284882	-918.282106
S3-TS1C2trans	-918.536748	0.294337	-918.226443	0.056736	0.054385	-918.283179	-918.280828
S3-TS1C5cis	-918.53849	0.294502	-918.227985	0.05749	0.05466	-918.285475	-918.282645
S3-TS1C5trans	-918.533298	0.293935	-918.223306	0.057831	0.054932	-918.281138	-918.278239
S3-TS2C5cis	-918.639133	0.298245	-918.325058	0.056329	0.053981	-918.381387	-918.379039

S3-TS2C5trans	-918.636734	0.298637	-918.322379	0.055862	0.053614	-918.378241	-918.375993
S3-TS3	-918.639284	0.296465	-918.326333	0.057348	0.055201	-918.383681	-918.381534
S3	-313.250595	0.203552	-313.039742	0.035268	0.035258	-313.07501	-313.075
S4-TS1C2cis	-918.532667	0.295179	-918.221917	0.055918	0.05365	-918.277834	-918.275567
S4-TS1C2trans	-918.534008	0.295368	-918.222967	0.056823	0.054214	-918.27979	-918.27718
S4-TS1C3cis	-918.531993	0.295181	-918.220992	0.057632	0.054593	-918.278624	-918.275585
S4-TS1C3trans	-918.532386	0.295032	-918.221608	0.057018	0.054211	-918.278626	-918.275819
S4	-313.248374	0.204799	-313.036629	0.034631	0.034641	-313.071259	-313.07127
S5-TS1C2cis	-957.842455	0.323614	-957.502277	0.057771	0.055532	-957.560048	-957.557808
S5-TS1C2trans	-957.846098	0.324649	-957.504864	0.058205	0.055707	-957.563068	-957.560571
S5-TS1C3cis	-957.844055	0.324259	-957.503216	0.058424	0.05584	-957.56164	-957.559057
S5-TS1C3trans	-957.844202	0.323915	-957.503615	0.058438	0.055896	-957.562053	-957.559511
S5	-352.559346	0.233789	-352.317684	0.036728	0.036734	-352.354412	-352.354418
S7-Int1C4	-918.639459	0.298231	-918.324318	0.059764	0.056472	-918.384082	-918.380791
S7-TS1C4ax	-918.541061	0.294707	-918.230575	0.056827	0.054257	-918.287401	-918.284831
S7-TS1C4eq	-918.536678	0.294406	-918.226479	0.056582	0.054157	-918.283061	-918.280636
S7-TS1C5ax	-918.538514	0.293819	-918.228674	0.05872	0.055122	-918.287394	-918.283795
S7-TS1C5eq	-918.536489	0.294377	-918.226205	0.057687	0.054717	-918.283892	-918.280922
S7-TS1C6ax	-918.538524	0.294523	-918.228349	0.056185	0.05388	-918.284535	-918.282229
S7-TS1C6eq	-918.537355	0.294516	-918.227115	0.056592	0.054122	-918.283706	-918.281237
S7-TS2'C4	-918.63433	0.29933	-918.319408	0.055717	0.053539	-918.375125	-918.372947
S7-TS2C4	-918.638015	0.298239	-918.323738	0.056911	0.054475	-918.380649	-918.378213
S7	-313.254189	0.204124	-313.04303	0.034838	0.03482	-313.077868	-313.077851
S8-Int1	-1075.893277	0.41126	-1075.460557	0.069103	0.065112	-1075.529661	-1075.525669
S8-Int1a	-1075.813617	0.410199	-1075.382358	0.069636	0.065452	-1075.451995	-1075.447811
S8-TS1ax	-1075.793417	0.407244	-1075.365732	0.067145	0.063224	-1075.432877	-1075.428956
S8-TS1eq	-1075.789562	0.406871	-1075.362213	0.068111	0.063702	-1075.430323	-1075.425915
S8-TS2'	-1075.887844	0.41204	-1075.455756	0.065331	0.06219	-1075.521087	-1075.517946
S8-TS2	-1075.891552	0.411106	-1075.46006	0.066294	0.062813	-1075.526354	-1075.522873
S8	-470.505758	0.316413	-470.17768	0.045879	0.04469	-470.223559	-470.222369
cis-P3-OH2	-918.689111	0.300821	-918.371609	0.059332	0.056073	-918.430941	-918.427682
cis-P3-OH5	-918.688458	0.300118	-918.371554	0.059548	0.056303	-918.431102	-918.427857
trans-P3-OH2	-918.686445	0.29966	-918.369818	0.060079	0.056681	-918.429897	-918.426498
trans-P3-OH5	-918.683489	0.300177	-918.366377	0.059813	0.05662	-918.42619	-918.422996

Table S12. Energies of the optimized structures for redox potential calculations ((u)M062X/6-31+G(d,p)/CPCM=acetonitrile)

Structure	E	ZPE	H	T.S	G(T)
S8-Int1a-1	-605.753447	0.106743	-605.636518	0.045161	-605.681679
S8-Int1a-2	-605.963762	0.104964	-605.848463	0.044648	-605.893111
S8-Int1a-3	-469.656506	0.303762	-469.338942	0.052248	-469.391190
S8-Int1a-4	-469.502802	0.307143	-469.182003	0.051423	-469.233426

Table S13. Energies of the optimized structures for isodesmic reaction calculations (ωB97X-D/6-311++G(d,p)/SMD = water/temperature = 273.15)

Structure	E	ZPE	H	T.S	T.qh-S	G(T)	qh-G(T)
S8-Int1a-5	-469.701008	0.306084	-469.383344	0.045079	0.044340	-469.428423	-469.427684
S8a	-393.121071	0.282945	-392.827941	0.042132	0.041385	-392.870073	-392.869326
S8a-Int1	-392.294375	0.268111	-392.015701	0.042481	0.041968	-392.058182	-392.057669

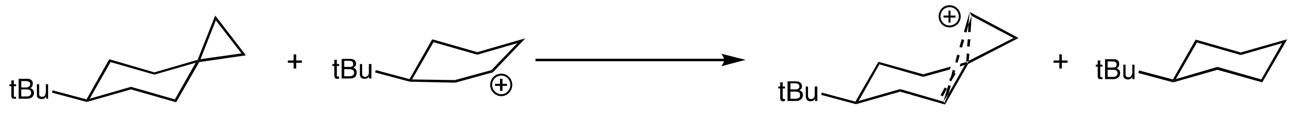

ΔG = –10.7 kcal/mol
(**S8-Int1a-5** is favored by 10.7 kcal/mol)

Scheme S2. Isodesmic reaction to evaluate the stability between **S8-Int1a-5** and **S8a-Int1**.

S5.2 Calculated Cartesian Coordinates

DIOXIRANE

Charge: 0

C 1.111390 -0.189144 -0.000006

C -0.374647 0.198183 -0.000072

C -1.351790 -0.938146 -0.000046

H -1.131515 -1.551240 -0.878560

H -1.131452 -1.551276 0.878428

C -2.807007 -0.492346 0.000018

H -3.450286 -1.373722 0.000011

H -3.042748 0.098971 0.886707

H	-3.042809	0.099031	-0.886616
F	1.402950	-0.922231	-1.082045
F	1.402907	-0.921970	1.082224
F	1.910526	0.875464	-0.000120
O	-0.632255	1.345304	0.737709
O	-0.632283	1.345394	-0.737692

There are no imaginary frequencies

P7-4-OH

Charge: 0

C	-2.776485	-2.061500	-0.452534
C	-1.797277	-1.667808	0.656774
C	-1.184021	-0.285451	0.432043
C	-2.265137	0.750187	0.188264
C	-3.245576	0.374390	-0.907496
C	-3.862577	-0.999408	-0.636369
H	-0.606486	0.000953	1.314556
H	-2.330281	-1.641586	1.614276
H	-0.994377	-2.404787	0.751644
H	-2.236691	-2.195343	-1.399015
H	-4.022091	1.141416	-0.983849
H	-2.726723	0.355207	-1.875545
H	-4.472046	-0.945115	0.274462
H	-4.532299	-1.279674	-1.454752
H	-0.641185	-0.573087	-1.448488
C	-2.798651	1.464236	1.400879
C	-1.920021	2.193547	0.410294
H	-2.371865	1.218214	2.367484
H	-3.856682	1.702513	1.415575
H	-0.907092	2.432027	0.716594
H	-2.386943	2.928240	-0.236936
H	-3.226370	-3.029963	-0.216387

C	2.634513	-0.876741	0.271363
C	2.374414	0.447903	-0.502703
C	2.019870	1.640209	0.321869
H	1.180352	1.368610	0.967411
H	2.868955	1.805898	0.998663
C	1.714045	2.873705	-0.508766
H	0.865564	2.693493	-1.173294
H	1.461010	3.707606	0.148329
H	2.572095	3.166281	-1.117676
F	2.432206	-1.947420	-0.495479
F	1.884052	-1.004161	1.369736
F	3.923621	-0.899388	0.665138
O	2.574621	0.436350	-1.690792
O	-0.218913	-0.300324	-0.629123

There are no imaginary frequencies

P7-5-OH

Charge: 0

C	-1.978064	2.099635	0.615842
C	-0.982800	0.952332	0.793949
C	-1.707056	-0.361065	1.093353
C	-2.770683	-0.658954	0.053023
C	-3.762378	0.475449	-0.123016
C	-3.037863	1.784803	-0.442439
H	-0.984490	-1.179248	1.151735
H	-0.626413	0.380491	-1.052590
H	-0.300865	1.188860	1.614693
H	-2.467461	2.274756	1.581393
H	-4.478684	0.228959	-0.912804
H	-4.333737	0.594989	0.807161
H	-2.562605	1.702103	-1.427768
H	-3.753381	2.609192	-0.508204

H	-2.181099	-0.269000	2.077898
C	-2.376158	-1.448576	-1.175685
C	-3.247009	-2.070634	-0.107347
H	-1.354505	-1.803915	-1.255759
H	-2.843476	-1.181971	-2.117644
H	-2.800834	-2.831474	0.524436
H	-4.295368	-2.225277	-0.339474
H	-1.431115	3.011714	0.359441
C	2.141139	-1.180738	0.229503
C	2.472612	0.166463	-0.473384
C	2.814800	1.313486	0.417284
H	1.988764	1.448102	1.121297
H	3.668581	0.984311	1.024670
C	3.124889	2.592126	-0.340200
H	2.263478	2.914695	-0.929614
H	3.372956	3.387457	0.364736
H	3.972190	2.458380	-1.016153
F	1.290988	-1.918413	-0.484284
F	1.632930	-1.026670	1.455775
F	3.280264	-1.890230	0.356965
O	2.529816	0.153186	-1.677294
O	-0.134582	0.826654	-0.356312

There are no imaginary frequencies

P7-6-OH

Charge: 0

C	0.906497	-0.955970	-0.747930
C	1.481573	0.370520	-1.247057
C	2.367196	1.050948	-0.199668
C	3.452202	0.106417	0.279690
C	2.900445	-1.204394	0.806143
C	2.016154	-1.881335	-0.245255

H	2.809547	1.959330	-0.619074
H	2.081562	0.157642	-2.139030
H	0.668914	1.033102	-1.555546
H	0.271044	-0.230292	0.976391
H	3.717804	-1.871853	1.094481
H	2.316558	-1.010448	1.715786
H	2.632552	-2.166784	-1.105423
H	1.569894	-2.796788	0.153888
H	1.760930	1.368927	0.659567
C	4.743225	0.067322	-0.494828
C	4.697096	0.675039	0.891762
H	4.851512	0.741822	-1.337715
H	5.239181	-0.890812	-0.609266
H	4.776278	1.754300	0.971540
H	5.164241	0.121929	1.699975
H	0.370089	-1.445926	-1.563688
C	-3.200013	-0.593104	-0.014653
C	-2.441967	0.679587	0.463786
C	-2.061587	1.660394	-0.594515
H	-1.559401	1.114144	-1.396838
H	-3.004255	2.019782	-1.029094
C	-1.213394	2.806876	-0.073485
H	-0.286972	2.434421	0.370351
H	-0.952873	3.475565	-0.895758
H	-1.747858	3.385978	0.682410
F	-3.091573	-1.589301	0.862963
F	-2.778526	-1.038190	-1.202638
F	-4.509428	-0.301689	-0.138812
O	-2.296371	0.820161	1.651376
O	-0.097074	-0.755814	0.259809

There are no imaginary frequencies

P7b-OH

Charge: 0

C	-4.029946	0.933349	0.587419
C	-3.667717	0.726266	-0.895790
C	-2.628811	-0.364124	-1.104453
C	-1.326104	-0.195195	-0.275073
C	-1.487574	0.786803	0.877314
C	-2.853301	0.671765	1.554104
H	-2.379555	-0.463283	-2.165134
H	-4.572392	0.510114	-1.472926
H	-3.256814	1.656852	-1.304719
H	-4.394491	1.956700	0.715120
H	-0.687794	0.620982	1.605965
H	-1.358937	1.799293	0.475917
H	-2.945444	-0.327961	1.990647
H	-2.897537	1.370852	2.392795
H	-0.014477	-0.414427	-1.705639
C	-2.832512	-1.759482	-0.457384
C	-1.390406	-1.694341	0.102496
H	-3.042171	-2.577125	-1.148376
H	-3.593384	-1.761466	0.325134
H	-0.686219	-2.279221	-0.493927
H	-1.248438	-1.936872	1.158169
C	2.449307	-0.923430	0.329440
C	2.519680	0.459974	-0.378825
C	2.301524	1.657565	0.483719
H	1.330925	1.539800	0.973381
H	3.046666	1.598628	1.288121
C	2.392984	2.970002	-0.273904
H	1.634038	3.021323	-1.058030
H	2.231136	3.803005	0.412329
H	3.374136	3.094526	-0.737128
F	2.121071	-1.896996	-0.519393
F	1.585159	-0.949500	1.347710
F	3.665978	-1.216358	0.829531

O	2.831506	0.464544	-1.542658
O	-0.170189	0.218168	-0.997875
H	-4.862411	0.277356	0.857551

There are no imaginary frequencies

P8a-OH

Charge: 0

C	-2.396660	-0.166817	-0.350055
C	-1.253310	-0.190392	0.676515
C	-0.161369	0.828025	0.377160
C	-0.722946	2.214886	0.167719
C	-1.866958	2.265290	-0.822262
C	-2.957005	1.261055	-0.443392
H	0.541057	0.854522	1.219468
H	-1.644247	0.034825	1.675077
H	-0.787222	-1.176857	0.728910
H	-1.960870	-0.406066	-1.330321
H	-2.275666	3.279697	-0.861190
H	-1.491884	2.033549	-1.826780
H	-3.385693	1.551609	0.523738
H	-3.762650	1.312623	-1.179429
H	1.026690	1.140106	-1.143450
C	-0.762477	3.117599	1.368491
C	0.238338	3.367402	0.263421
H	-0.387728	2.732867	2.310977
H	-1.603718	3.796792	1.452965
H	1.281149	3.143526	0.467707
H	0.071346	4.216522	-0.390221
C	2.682172	-1.670754	0.328577
C	3.189259	-0.453397	-0.497618
C	3.537787	0.779163	0.270032
H	2.691548	1.033918	0.913948

H	4.346738	0.491538	0.955249
C	3.938990	1.945231	-0.616024
H	3.127227	2.218009	-1.295684
H	4.171469	2.814076	0.002076
H	4.819870	1.705439	-1.215121
F	1.883767	-2.457616	-0.391742
F	2.025576	-1.317486	1.437788
F	3.743095	-2.408308	0.709285
O	3.347719	-0.623129	-1.680065
O	0.547498	0.383945	-0.790219
C	-3.477341	-1.259494	-0.099439
C	-4.238128	-1.034854	1.213734
H	-4.969113	-1.836382	1.363597
H	-3.567208	-1.035636	2.077737
H	-4.784526	-0.087423	1.208421
C	-4.484486	-1.261709	-1.259344
H	-3.973832	-1.378451	-2.221510
H	-5.186436	-2.094464	-1.148557
H	-5.072826	-0.341477	-1.297405
C	-2.815429	-2.645351	-0.057073
H	-3.580605	-3.427986	-0.038957
H	-2.187683	-2.808496	-0.940111
H	-2.191476	-2.777507	0.830510

There are no imaginary frequencies

P8b-OH

Charge: 0

C	2.916217	0.670756	0.111784
C	2.478155	-0.045782	1.424669
C	1.274618	-0.974328	1.309786
C	0.088662	-0.518048	0.419911
C	0.477366	0.566922	-0.571008

C	1.924205	0.442051	-1.043082
H	0.900239	-1.211091	2.310651
H	3.304823	-0.625568	1.844728
H	2.245386	0.716235	2.175206
H	2.866690	1.747978	0.318655
H	-0.210862	0.541450	-1.422170
H	0.349303	1.537624	-0.075877
H	2.079992	-0.531443	-1.519547
H	2.098939	1.190305	-1.819016
H	-1.375629	-0.747750	1.693010
C	1.369783	-2.276957	0.472687
C	0.001001	-1.946582	-0.167660
H	1.421163	-3.202783	1.047981
H	2.189900	-2.268032	-0.244383
H	-0.818412	-2.509927	0.284953
H	-0.076273	-2.017251	-1.255191
C	-3.693292	-0.647610	-0.530161
C	-3.620039	0.660270	0.309666
C	-3.151172	1.883598	-0.404681
H	-2.173285	1.660424	-0.839997
H	-3.828159	2.021701	-1.258065
C	-3.103387	3.115592	0.481139
H	-2.411101	2.970312	1.313706
H	-2.762104	3.973851	-0.100204
H	-4.088354	3.350069	0.890441
F	-3.561245	-1.733290	0.231688
F	-2.774043	-0.703937	-1.498086
F	-4.902234	-0.714231	-1.121431
O	-4.031419	0.607394	1.440955
O	-1.071793	-0.051239	1.102964
C	4.401327	0.411850	-0.278727
C	4.696584	-1.074865	-0.511688
H	4.090776	-1.483663	-1.325730
H	5.748890	-1.207963	-0.784040
H	4.509398	-1.674270	0.383821

C	4.745129	1.184540	-1.560567
H	4.474077	2.242032	-1.467926
H	5.820745	1.129750	-1.757409
H	4.229472	0.777410	-2.434350
C	5.314255	0.934997	0.840192
H	6.366240	0.799733	0.568474
H	5.145442	2.003759	1.011322
H	5.149629	0.412549	1.786490

There are no imaginary frequencies

P8c-OH

Charge: 0

C	-2.278716	-0.245131	-0.398879
C	-1.138133	-0.011373	0.602245
C	-0.183501	1.082918	0.135948
C	-0.935821	2.383475	-0.098744
C	-2.074174	2.185292	-1.079585
C	-3.025096	1.078158	-0.623176
H	0.576032	1.233586	1.930960
H	-1.547102	0.287744	1.576544
H	-0.558644	-0.923812	0.758429
H	-1.810342	-0.516158	-1.357770
H	-2.618341	3.126268	-1.205744
H	-1.658014	1.922551	-2.061287
H	-3.511894	1.388569	0.309321
H	-3.812268	0.955188	-1.370562
H	0.265231	0.783999	-0.817734
C	-1.144380	3.335541	1.045770
C	-0.209213	3.693539	-0.090939
H	-0.694469	3.104101	2.005534
H	-2.098396	3.848448	1.103857
H	0.853883	3.688913	0.121555

H	-0.534460	4.450721	-0.796567
C	3.547404	-0.011898	-0.373317
C	2.757683	-1.147096	0.339877
C	1.867172	-1.974249	-0.527344
H	1.075523	-1.313614	-0.900089
H	2.446028	-2.257842	-1.414136
C	1.296448	-3.187030	0.186231
H	0.706246	-2.891822	1.056202
H	0.646710	-3.742600	-0.491700
H	2.093496	-3.854159	0.522534
F	4.010466	0.889710	0.489296
F	2.814975	0.626111	-1.292483
F	4.607249	-0.548809	-1.007440
O	2.965108	-1.317076	1.513483
O	0.918244	1.210692	1.032019
C	-3.197486	-1.445562	-0.028043
C	-4.244938	-1.654003	-1.131898
H	-4.810402	-2.572681	-0.945899
H	-4.963593	-0.832003	-1.179246
H	-3.767993	-1.745810	-2.113940
C	-3.922043	-1.230121	1.306801
H	-4.556690	-2.093896	1.531001
H	-3.217957	-1.114628	2.135867
H	-4.565271	-0.345861	1.281047
C	-2.357353	-2.727909	0.066541
H	-3.009785	-3.599775	0.178434
H	-1.755426	-2.870792	-0.837650
H	-1.680230	-2.713981	0.923736

There are no imaginary frequencies

S1-TS1C2cis

Charge: 0

C	-1.852636	-0.564363	1.123047
C	-1.537553	-0.880302	-0.339647
C	-2.717469	-0.457967	-1.150467
C	-3.884480	-0.308624	-0.178232
C	-3.388087	-0.663643	1.212512
H	-1.341515	-1.239960	1.809150
H	-1.531574	0.451356	1.366115
H	-1.237541	-1.913998	-0.524786
H	-0.596741	-0.276032	-0.682214
H	-2.859808	-0.873709	-2.139267
H	-4.871646	-0.635172	-0.482311
H	-3.797138	-0.013577	1.990550
H	-3.686113	-1.690358	1.444391
C	-3.304125	0.901026	-0.840974
H	-3.887189	1.360351	-1.629939
H	-2.710124	1.592257	-0.250707
C	2.293683	-0.761489	0.062607
C	1.578667	0.578684	-0.220405
C	1.249736	1.353452	1.036237
H	0.735800	0.681750	1.728420
H	2.202014	1.623528	1.502269
C	0.409246	2.593826	0.769111
H	-0.557759	2.332384	0.333546
H	0.225812	3.118222	1.708875
H	0.917996	3.282545	0.091321
F	2.542751	-1.445441	-1.056729
F	1.560694	-1.551259	0.861892
F	3.468975	-0.546888	0.674655
O	2.154897	1.252096	-1.260030
O	0.565334	0.416543	-1.168284

1 imaginary frequency: –1019.30 cm^{-1}

S1-TS1C2trans

Charge: 0

C	2.632911	-0.528557	1.490504
C	1.539418	-1.062777	0.574033
C	1.930676	-0.697987	-0.820870
C	2.980240	0.389343	-0.730916
C	3.214971	0.684706	0.741558
H	2.257966	-0.273856	2.483156
H	3.389962	-1.307414	1.627938
H	0.574069	-0.446778	0.823153
H	1.218683	-2.091636	0.749687
H	1.186982	-0.713215	-1.606472
H	2.976412	1.208955	-1.439173
H	4.270603	0.842548	0.978283
H	2.673403	1.594265	1.017953
C	3.373233	-0.974774	-1.212566
H	3.550212	-1.096692	-2.274501
H	3.977752	-1.609870	-0.573721
C	-2.254899	-0.695806	-0.170199
C	-1.482495	0.584697	0.221441
C	-1.007949	1.383855	-0.972429
H	-0.483577	0.709028	-1.652927
H	-1.903425	1.730959	-1.496576
C	-0.119205	2.558999	-0.592166
H	0.790270	2.223478	-0.091140
H	0.171797	3.101217	-1.493854
H	-0.640165	3.255942	0.067757
F	-2.642349	-1.395190	0.899070
F	-1.508740	-1.509038	-0.933322
F	-3.357285	-0.384253	-0.870088
O	-2.096307	1.255843	1.240254
O	-0.563314	0.321558	1.240889

1 imaginary frequency: –1042.57 cm^{-1}

S1-TS1C3cis

Charge: 0

C	-1.449651	-1.062636	0.506391
C	-2.498357	-1.666502	-0.419904
C	-3.581329	-0.607958	-0.530613
C	-3.365346	0.401860	0.571665
C	-2.152404	-0.028992	1.376222
H	-0.815008	-1.774735	1.032459
H	-0.680931	-0.494283	-0.176220
H	-2.907039	-2.566812	0.053861
H	-2.077599	-1.971215	-1.382321
H	-4.580461	-0.917443	-0.811860
H	-4.206489	0.836282	1.097899
H	-1.496544	0.796464	1.665026
H	-2.478308	-0.518680	2.301704
C	-3.144993	0.794584	-0.867095
H	-3.887702	1.444465	-1.314882
H	-2.132276	0.958926	-1.220508
C	2.330487	-0.653495	0.129471
C	1.433906	0.518321	-0.333355
C	1.208778	1.556490	0.743654
H	0.784932	1.048423	1.614193
H	2.190457	1.935414	1.042434
C	0.305265	2.696291	0.295196
H	-0.686357	2.332822	0.017623
H	0.186733	3.409223	1.113435
H	0.729548	3.228861	-0.558471
F	2.416894	-1.613917	-0.794030
F	1.874456	-1.219154	1.256789
F	3.575892	-0.214968	0.374189
O	1.783715	0.941455	-1.585718
O	0.308001	0.058153	-1.018870

1 imaginary frequency: –967.46 cm^{-1}

S1-TS1C3trans

Charge: 0

C	1.481002	-1.251757	0.702303
C	1.782760	-1.217941	-0.790616
C	2.636595	0.027179	-0.994888
C	3.127307	0.488598	0.361383
C	2.569027	-0.457375	1.417519
H	1.235867	-2.227738	1.124360
H	0.874718	-1.202461	-1.395049
H	2.326940	-2.130310	-1.064297
H	2.333951	0.752322	-1.740756
H	3.187081	1.546045	0.587474
H	3.335592	-1.145686	1.793313
H	2.174763	0.080730	2.282368
C	4.095180	-0.081557	-0.640006
H	4.784312	0.604747	-1.119108
H	4.493984	-1.074276	-0.450045
H	0.496076	-0.637546	0.866712
C	-2.312030	-0.584278	-0.251979
C	-1.440430	0.584225	0.262505
C	-0.823607	1.406634	-0.846913
H	-0.245377	0.734061	-1.485496
H	-1.643770	1.796745	-1.456534
C	0.051566	2.540704	-0.332421
H	0.862170	2.163868	0.293752
H	0.497137	3.068001	-1.178233
H	-0.529124	3.261011	0.247350
F	-2.797898	-1.324260	0.747641
F	-1.622737	-1.404189	-1.059402
F	-3.357292	-0.114269	-0.951163
O	-2.049210	1.240935	1.294348

O -0.599190 0.167610 1.298050

1 imaginary frequency: –968.43 cm^{-1}

S1

Charge: 0

C 1.394177 0.000128 0.414991

C 0.671780 1.222715 -0.181441

C -0.753810 0.754833 -0.417898

C -0.753618 -0.754825 -0.418060

C 0.672140 -1.222773 -0.181386

H 2.463348 0.000406 0.193959

H 1.292619 0.000050 1.503401

H 1.117895 1.497976 -1.143105

H 0.735976 2.097748 0.472824

H -1.377349 1.307386 -1.111142

H -1.377003 -1.307348 -1.111464

H 0.736460 -2.098005 0.472149

H 1.118284 -1.496863 -1.143392

C -1.432567 -0.000192 0.697469

H -2.516595 -0.000253 0.699230

H -0.982247 -0.000416 1.685486

There are no imaginary frequencies

S2-TS1C2cis

Charge: 0

C 2.806059 1.366164 -0.307548

C 1.820179 0.735631 -1.283990

C 1.303254 -0.608056 -0.798456

C 2.298219 -1.502647 -0.120320

C	2.695304	-1.249286	1.325331
C	3.675044	-0.959041	0.236330
C	4.031751	0.467638	-0.129507
H	4.592147	0.453837	-1.071467
H	4.700473	0.883851	0.631077
H	4.485262	-1.672701	0.127955
H	2.215858	-0.411955	1.821303
H	2.858874	-2.119244	1.950089
H	2.244146	-2.547178	-0.403567
H	0.749995	-1.135981	-1.576746
H	0.474130	-0.379226	-0.007254
H	0.982521	1.406457	-1.489410
H	2.325429	0.565685	-2.244659
H	2.312837	1.537285	0.656195
H	3.122399	2.345309	-0.676005
C	-2.525052	-0.707757	-0.312442
C	-1.743314	0.387596	0.447097
C	-1.680508	1.701624	-0.297075
H	-1.262184	1.506852	-1.288327
H	-2.710483	2.038193	-0.446964
C	-0.868704	2.763184	0.430978
H	0.165523	2.444556	0.575490
H	-0.859793	3.681612	-0.159080
H	-1.299292	2.993174	1.407727
F	-2.505596	-1.876950	0.331636
F	-2.025062	-0.916010	-1.539734
F	-3.810587	-0.347670	-0.457238
O	-2.057625	0.400271	1.776213
O	-0.554877	-0.112166	0.978699

1 imaginary frequency: –1090.37 cm^{-1}

S2-TS1C2trans

Charge: 0

C	2.792379	0.743819	1.303161
C	2.339615	-0.675002	1.643718
C	1.292376	-1.150545	0.657001
C	1.637225	-0.967432	-0.782129
C	2.983953	-1.432011	-1.301847
C	2.697426	0.038519	-1.169481
C	3.423380	0.841110	-0.090841
H	3.453550	1.891197	-0.393561
H	4.464130	0.503533	-0.037622
H	2.513294	0.566737	-2.098502
H	3.678766	-1.865860	-0.590356
H	2.998567	-1.877641	-2.289528
H	0.812134	-1.102055	-1.471416
H	0.363113	-0.461648	0.855626
H	0.888476	-2.141453	0.880925
H	3.193866	-1.364542	1.615216
H	1.934312	-0.720883	2.657417
H	3.504924	1.101692	2.050842
H	1.918154	1.401627	1.359017
C	-2.485228	-0.631859	-0.089958
C	-1.632889	0.634456	0.152314
C	-1.140075	1.274778	-1.127003
H	-0.683994	0.503035	-1.751180
H	-2.027560	1.629784	-1.659554
C	-0.163451	2.418029	-0.892434
H	0.105252	2.867625	-1.850306
H	-0.602627	3.196839	-0.265283
H	0.752996	2.066313	-0.416459
F	-1.801049	-1.568225	-0.764766
F	-3.576823	-0.331485	-0.811314
F	-2.898505	-1.185503	1.052638
O	-2.189068	1.444978	1.099822
O	-0.716960	0.431202	1.187767

1 imaginary frequency: –1055.96 cm^{-1}

S2-TS1C3cis

Charge: 0

C	1.492806	-0.690751	-0.785805
C	1.260310	-0.482908	0.692321
C	2.230748	0.490568	1.333083
C	3.679554	0.193060	0.935018
C	4.225390	0.749050	-0.358313
C	3.977907	-0.728217	-0.227040
C	2.841942	-1.403491	-0.969959
H	2.743259	-2.428757	-0.596393
H	3.074538	-1.474154	-2.037451
H	4.866406	-1.342382	-0.121991
H	3.538105	1.326936	-0.968967
H	5.252036	1.096644	-0.376968
H	4.375353	0.153031	1.765415
H	2.133918	0.446865	2.419522
H	1.969335	1.509071	1.026294
H	0.192999	-0.023601	0.855320
H	1.190206	-1.433198	1.230690
H	1.494223	0.273862	-1.303553
H	0.701140	-1.299870	-1.225537
C	-2.602997	-0.776761	-0.004812
C	-1.935314	0.612937	0.109507
C	-1.488951	1.174075	-1.223310
H	-0.948621	0.393749	-1.764713
H	-2.396423	1.390581	-1.794530
C	-0.626163	2.420608	-1.084404
H	0.306605	2.200583	-0.560247
H	-0.374888	2.801010	-2.076369
H	-1.149572	3.211033	-0.542034
F	-2.990207	-1.246463	1.183625

F	-1.773876	-1.683915	-0.542608
F	-3.691509	-0.710018	-0.787651
O	-2.633489	1.435183	0.949710
O	-1.016472	0.641458	1.161978

1 imaginary frequency: –944.69 cm^{-1}

S2-TS1C3trans

Charge: 0

C	2.312317	-1.277912	1.326130
C	1.196057	-1.478354	0.325502
C	1.556687	-1.113675	-1.099614
C	2.392553	0.151837	-1.227866
C	3.886962	0.082219	-1.015375
C	3.020739	0.777043	-0.002274
C	2.771203	0.181728	1.368410
H	1.989499	0.770594	1.861299
H	3.670196	0.270159	1.986431
H	3.072915	1.861038	-0.011046
H	4.297080	-0.888603	-0.752975
H	4.526333	0.690054	-1.645268
H	2.054353	0.845685	-1.989613
H	0.648315	-1.025141	-1.698977
H	2.104984	-1.970364	-1.514654
H	0.716428	-2.456427	0.395591
H	0.340938	-0.750033	0.674287
H	3.143335	-1.932341	1.029640
H	1.997948	-1.599748	2.321338
C	-2.496065	-0.522908	-0.286143
C	-1.584726	0.569219	0.319983
C	-1.007123	1.510192	-0.714484
H	-0.438262	0.912310	-1.430673
H	-1.845847	1.949188	-1.262471

C	-0.133673	2.599001	-0.107606
H	0.677598	2.173485	0.485238
H	0.310094	3.196796	-0.906130
H	-0.716070	3.265925	0.531240
F	-2.930336	-1.383799	0.637729
F	-1.865169	-1.233874	-1.232282
F	-3.575802	0.036541	-0.855326
O	-2.135496	1.104479	1.449396
O	-0.701713	0.026430	1.257563

1 imaginary frequency: –964.47 cm^{-1}

S2

Charge: 0

C	1.325570	-0.764231	0.472931
C	1.566336	0.605125	-0.158389
C	0.345094	1.515684	0.000254
C	-0.972184	0.830214	-0.354000
C	-1.698463	0.015657	0.690909
C	-1.038108	-0.677991	-0.468793
C	0.216035	-1.508716	-0.273996
H	0.598475	-1.788280	-1.263133
H	-0.031909	-2.442825	0.242188
H	-1.715385	-1.062284	-1.225138
H	-1.223220	-0.062568	1.664443
H	-2.781845	0.052730	0.716936
H	-1.609506	1.380821	-1.037671
H	0.467927	2.402408	-0.627804
H	0.303898	1.874348	1.035716
H	2.442030	1.087143	0.286449
H	1.785407	0.466422	-1.224769
H	1.058331	-0.638629	1.529471
H	2.240117	-1.363733	0.449821

There are no imaginary frequencies

S3-Int1

Charge: 0

C	1.989700	1.841109	-0.661094
C	2.976088	1.660343	0.489283
C	2.629309	0.403866	1.283586
C	2.636467	-0.855094	0.471562
C	3.541734	-0.986253	-0.636923
C	2.182763	-0.629590	-1.285702
C	1.609981	0.596092	-1.362512
H	0.715003	0.659672	-1.975591
H	-0.757125	-0.079318	1.844077
H	1.670864	-1.469753	-1.735820
H	4.314885	-0.240680	-0.771075
H	3.826635	-1.992522	-0.922544
C	1.902798	-2.034488	1.012667
H	1.912749	-2.880534	0.325598
H	0.877757	-1.771329	1.277431
H	2.420879	-2.328987	1.932963
H	1.661998	0.507845	1.779405
H	3.379823	0.237302	2.065794
H	4.000593	1.607963	0.117577
H	2.926777	2.527259	1.149204
H	2.378009	2.538775	-1.414321
H	1.052913	2.290832	-0.310575
C	-2.088442	-1.029443	-0.275862
C	-2.059787	0.351582	0.453709
C	-2.117572	1.468168	-0.608629
H	-1.301925	1.339711	-1.325779
H	-3.054399	1.349718	-1.161073
C	-2.050299	2.863136	-0.000178

H	-2.867630	3.029991	0.706127
H	-1.106496	3.023767	0.527651
H	-2.127231	3.621103	-0.784085
F	-1.128767	-1.180782	-1.216460
F	-3.264095	-1.243783	-0.905500
F	-1.928158	-2.055332	0.587239
O	-3.017531	0.383188	1.377705
O	-0.726385	0.423272	1.023304

There are no imaginary frequencies

S3-Int2

Charge: 0

C	3.463343	1.302288	-1.040255
C	1.946685	1.096915	-1.117587
C	1.558451	-0.382089	-1.189238
C	1.405880	-1.100275	0.163636
C	2.249701	-0.464613	1.282715
C	3.710780	-0.271363	0.958632
C	4.208605	0.495663	-0.012380
H	5.292682	0.545350	-0.101758
H	-0.100542	-1.311643	1.416375
H	4.418426	-0.798619	1.593680
H	1.794034	0.496682	1.538755
H	2.155615	-1.096105	2.171457
C	1.720500	-2.585168	0.014198
H	1.476557	-3.122028	0.936193
H	2.782254	-2.734686	-0.201142
H	1.138105	-3.015882	-0.805573
H	2.310374	-0.910971	-1.784043
H	0.608626	-0.505787	-1.717334
H	1.451298	1.583028	-0.273548
H	1.584688	1.608067	-2.014476

H	3.691379	2.364632	-0.887410
H	3.896092	1.053871	-2.018314
C	-2.909458	-0.415907	-0.367832
C	-2.226872	0.633661	0.556372
C	-1.539596	1.771021	-0.121040
H	-0.844269	1.352713	-0.853790
H	-2.308839	2.289687	-0.708445
C	-0.841869	2.709259	0.847248
H	-0.345722	3.508703	0.294202
H	-1.551056	3.162707	1.543014
H	-0.084936	2.175263	1.427830
F	-2.276072	-0.584330	-1.532575
F	-4.159312	0.001343	-0.648375
F	-3.007019	-1.604722	0.226220
O	-2.375977	0.494283	1.743692
O	0.018472	-0.955778	0.530067

There are no imaginary frequencies

S3-TS1C2cis

Charge: 0

C	3.196181	1.495204	-0.947652
C	1.862480	1.540794	-0.196001
C	1.218310	0.170970	-0.269607
C	2.057434	-0.997174	0.136222
C	2.823728	-0.925925	1.435296
C	3.574194	-0.787460	0.140849
C	4.182866	0.553315	-0.254427
H	5.036787	0.375167	-0.912970
H	4.574725	1.048493	0.641108
H	4.132771	-1.664497	-0.170355
H	2.714522	-0.030179	2.038986
H	2.920536	-1.848754	1.996745

C	1.513870	-2.341359	-0.286211
H	2.182748	-3.149782	0.019720
H	1.390382	-2.390640	-1.372771
H	0.535415	-2.520565	0.172462
H	0.767112	0.002014	-1.252487
H	0.295755	0.172612	0.441480
H	2.035304	1.836046	0.844723
H	1.191165	2.280803	-0.637861
H	3.623964	2.499123	-1.016088
H	3.012669	1.151513	-1.972465
C	-2.433729	-0.866451	-0.367095
C	-2.052867	0.442410	0.362262
C	-2.047063	1.654747	-0.541949
H	-1.316141	1.470489	-1.334629
H	-3.028771	1.717860	-1.019919
C	-1.721866	2.945778	0.195960
H	-1.697661	3.774250	-0.514557
H	-2.474723	3.172001	0.953716
H	-0.746138	2.890440	0.683099
F	-1.680963	-1.073858	-1.457305
F	-3.715077	-0.814912	-0.766891
F	-2.302327	-1.936527	0.420375
O	-2.664720	0.523907	1.582546
O	-0.948572	0.267616	1.192524

1 imaginary frequency: –1078.90 cm^{-1}

S3-TS1C2trans

Charge: 0

C	2.228052	-1.872950	0.759940
C	1.524855	-1.577021	-0.562524
C	1.207098	-0.100113	-0.667877
C	2.319752	0.848474	-0.342459

C	3.638476	0.629823	-1.062618
C	3.519642	0.308881	0.405738
C	3.572165	-1.147284	0.862131
H	3.922346	-1.182368	1.896975
H	4.315704	-1.684864	0.263197
H	3.990064	1.022938	1.074186
H	3.716685	-0.208344	-1.747238
H	4.179111	1.520819	-1.362767
C	1.888951	2.274331	-0.092069
H	2.755021	2.928788	0.033561
H	1.279885	2.338139	0.814829
H	1.290977	2.654407	-0.927484
H	0.677626	0.177215	-1.584338
H	2.164274	-1.869386	-1.405858
H	0.602676	-2.154858	-0.655303
H	2.380566	-2.948996	0.877208
H	1.572413	-1.551398	1.577853
H	0.395004	0.099544	0.154341
C	-2.368770	-0.949574	-0.302242
C	-1.936771	0.369602	0.378295
C	-2.085847	1.577694	-0.520854
H	-1.356021	1.468869	-1.328310
H	-3.077890	1.538248	-0.979415
C	-1.890048	2.897336	0.211744
H	-1.925568	3.719921	-0.505248
H	-2.675696	3.060085	0.952115
H	-0.924732	2.934689	0.719717
F	-1.805760	-1.099463	-1.510235
F	-3.700683	-0.962251	-0.475417
F	-2.049540	-2.021188	0.427274
O	-2.396831	0.434997	1.663761
O	-0.726160	0.230940	1.055508

1 imaginary frequency: –1020.19 cm^{-1}

S3-TS1C5cis

Charge: 0

C	-1.916118	-1.816483	-0.992022
C	-2.896909	-0.835183	-1.622178
C	-3.793850	-0.218609	-0.548896
C	-3.034336	0.414069	0.605020
C	-1.879735	1.308755	0.296987
C	-1.613723	-0.074792	0.877054
C	-1.010115	-1.156827	0.036111
H	-0.494319	-1.898556	0.647851
H	-0.136634	-0.678606	-0.572946
H	-1.302389	-0.075986	1.916045
H	-1.600287	1.464521	-0.739022
H	-1.697420	2.154671	0.950713
C	-3.887277	0.746709	1.806876
H	-3.293352	1.206404	2.601346
H	-4.360448	-0.154424	2.210229
H	-4.681827	1.448239	1.529472
H	-4.437778	-1.007257	-0.139490
H	-4.458298	0.532834	-0.990038
H	-2.349181	-0.056150	-2.164210
H	-3.517142	-1.351579	-2.359333
H	-1.310371	-2.317112	-1.751987
H	-2.481871	-2.603059	-0.474795
C	2.582175	-0.540333	0.690514
C	2.021674	0.318658	-0.465742
C	1.596283	1.701810	-0.028184
H	0.785219	1.579647	0.694505
H	2.436157	2.155979	0.505512
C	1.157098	2.585891	-1.186724
H	0.780127	3.534036	-0.798353
H	1.988942	2.802461	-1.859816
H	0.358309	2.118205	-1.765854

F	1.751034	-0.559086	1.743711
F	3.756200	-0.044038	1.112257
F	2.791324	-1.806368	0.321680
O	2.765200	0.184835	-1.602844
O	1.126617	-0.406189	-1.253600

1 imaginary frequency: –1079.31 cm^{-1}

S3-TS1C5trans

Charge: 0

C	1.489585	1.557911	-0.359900
C	2.919996	1.650472	-0.900894
C	3.876047	0.847940	-0.018778
C	3.439578	-0.587549	0.227584
C	3.015566	-1.418135	-0.957455
C	1.981395	-0.928717	0.039474
C	0.996172	0.123331	-0.399429
H	0.509493	-0.129739	-1.345332
H	1.580319	-1.695253	0.695203
H	3.018828	-0.955971	-1.939407
H	3.254342	-2.476124	-0.950798
C	4.195029	-1.284914	1.334629
H	3.852143	-2.315559	1.461029
H	4.059703	-0.761837	2.287192
H	5.267972	-1.309858	1.114863
H	3.949528	1.348808	0.954829
H	4.883841	0.848995	-0.449461
H	2.950152	1.292386	-1.935681
H	3.234907	2.697019	-0.920574
H	0.810167	2.199992	-0.924893
H	1.492097	1.914211	0.676812
H	0.106599	0.066891	0.358749
C	-2.619906	-1.002398	-0.325890

C	-2.225387	0.287594	0.430713
C	-2.305313	1.532250	-0.425982
H	-1.614922	1.399431	-1.264129
H	-3.313596	1.582564	-0.846400
C	-1.977088	2.804818	0.342143
H	-2.012374	3.658623	-0.337258
H	-2.695661	2.981645	1.144973
H	-0.976459	2.760830	0.776845
F	-1.924490	-1.151712	-1.462963
F	-3.922113	-0.967111	-0.651566
F	-2.420206	-2.097416	0.411634
O	-2.775808	0.310733	1.683008
O	-1.057922	0.107202	1.173516

1 imaginary frequency: –973.31 cm^{-1}

S3-TS2C5cis

Charge: 0

C	1.352348	-1.864711	-0.057783
C	2.547514	-1.852796	0.881822
C	3.685686	-1.038860	0.268396
C	3.294463	0.381076	-0.090409
C	2.391625	1.107029	0.789803
C	1.782282	0.599350	-0.545533
C	0.971590	-0.548540	-0.608118
H	0.226357	-0.571657	-1.394066
H	-0.737726	-0.683961	1.470114
H	1.586839	1.378527	-1.270209
H	2.010677	0.645057	1.690448
H	2.449228	2.188570	0.811727
C	4.293837	1.140298	-0.924523
H	3.936949	2.144426	-1.163002
H	4.503493	0.608154	-1.855682

H	5.230576	1.233138	-0.366428
H	4.042190	-1.538493	-0.639064
H	4.531431	-0.985881	0.960407
H	2.261748	-1.453062	1.857702
H	2.880297	-2.878644	1.048600
H	0.477471	-2.371839	0.353474
H	1.600835	-2.433508	-0.969851
C	-2.687266	-0.648949	-0.331538
C	-1.986947	0.497420	0.465901
C	-1.744652	1.692915	-0.474096
H	-1.145483	1.376057	-1.332774
H	-2.719182	2.006452	-0.860143
C	-1.066945	2.862799	0.227806
H	-0.925330	3.689909	-0.472523
H	-1.670892	3.230044	1.061129
H	-0.085279	2.581639	0.616676
F	-2.067839	-0.986339	-1.485803
F	-3.948984	-0.316681	-0.668694
F	-2.768893	-1.782614	0.393979
O	-2.657731	0.742560	1.575464
O	-0.640265	-0.037111	0.760507

1 imaginary frequency: –160.77 cm^{-1}

S3-TS2C5trans

Charge: 0

C	1.507337	1.540802	-1.062871
C	2.643361	1.826099	-0.061487
C	2.863172	0.707974	0.959123
C	2.898730	-0.684161	0.355492
C	3.369573	-0.851995	-1.020415
C	1.835148	-0.946165	-0.751979
C	1.029277	0.151104	-1.129221

H	0.159244	-0.034275	-1.745375
H	-0.029308	0.553965	1.248543
H	1.406277	-1.936346	-0.821629
H	3.713499	-0.002733	-1.596507
H	3.786132	-1.808327	-1.312365
C	3.071554	-1.811336	1.342498
H	3.039232	-2.783840	0.846267
H	2.285043	-1.778613	2.101159
H	4.037550	-1.715114	1.846928
H	2.082301	0.724581	1.726603
H	3.810969	0.879445	1.477944
H	3.571683	1.997190	-0.609940
H	2.422730	2.753887	0.468433
H	1.849698	1.728968	-2.093256
H	0.648173	2.200047	-0.938737
C	-2.361127	-0.982032	-0.219479
C	-1.846764	0.288660	0.520243
C	-2.129783	1.537982	-0.335667
H	-1.609691	1.454830	-1.294309
H	-3.202163	1.562567	-0.552397
C	-1.725354	2.823324	0.376550
H	-1.845398	3.680067	-0.291117
H	-2.342643	2.996600	1.260680
H	-0.680160	2.795203	0.699976
F	-1.873216	-1.128613	-1.472884
F	-3.703000	-0.956522	-0.338479
F	-2.055624	-2.112780	0.439612
O	-2.299270	0.323835	1.754653
O	-0.369150	0.040114	0.506364

1 imaginary frequency: –185.69 cm^{-1}

S3-TS3

Charge: 0

C	3.734122	0.873586	-1.085196
C	2.216721	1.023361	-1.211213
C	1.499960	-0.315405	-1.123996
C	1.462781	-0.959337	0.201209
C	2.075208	-0.296954	1.360923
C	3.570813	-0.474993	1.082819
C	4.245520	0.051312	0.060246
H	5.312741	-0.156377	0.025494
H	-0.935087	-0.769754	1.686613
H	4.088604	-1.113690	1.789986
H	1.812975	0.758491	1.406385
H	1.804094	-0.789410	2.291507
C	1.080167	-2.373858	0.278662
H	0.652046	-2.630008	1.246989
H	2.025458	-2.929563	0.179060
H	0.435959	-2.668150	-0.549164
H	1.925494	-1.051726	-1.820451
H	0.451663	-0.243506	-1.433262
H	1.822631	1.712518	-0.460575
H	1.991761	1.468370	-2.182473
H	4.204208	1.862324	-1.040071
H	4.122086	0.410987	-2.001605
C	-2.597111	-0.506012	-0.448644
C	-1.917976	0.487950	0.545260
C	-1.464912	1.742121	-0.227240
H	-0.812919	1.460278	-1.058453
H	-2.359252	2.197680	-0.662742
C	-0.753771	2.752452	0.663789
H	-0.460135	3.629052	0.080692
H	-1.401893	3.092230	1.475402
H	0.150694	2.327413	1.106684
F	-1.838018	-0.823178	-1.523380
F	-3.751461	-0.010803	-0.941129
F	-2.905583	-1.676041	0.146473

O	-2.720288	0.702998	1.579014
O	-0.690301	-0.203287	0.946309

1 imaginary frequency: –80.21 cm^{-1}

S3

Charge: 0

C	1.933522	0.081547	-0.502395
C	1.366157	1.137420	0.442952
C	-0.103291	1.405057	0.116082
C	-0.957764	0.150901	0.021217
C	-0.908129	-0.851336	1.142517
C	-0.266822	-1.179610	-0.190312
C	1.252382	-1.274534	-0.297877
H	1.512074	-1.939948	-1.125971
H	1.653732	-1.739967	0.610101
H	-0.816860	-1.888698	-0.801806
H	-0.235883	-0.662600	1.974348
H	-1.830140	-1.357226	1.410351
C	-2.293602	0.368948	-0.652673
H	-2.879241	-0.554226	-0.683495
H	-2.159580	0.720526	-1.681604
H	-2.881481	1.122683	-0.116342
H	-0.148946	1.916096	-0.854611
H	-0.544069	2.090145	0.850084
H	1.474301	0.799073	1.480766
H	1.929576	2.071064	0.357275
H	3.013149	-0.025414	-0.360795
H	1.778655	0.418133	-1.535361

There are no imaginary frequencies

S4-TS1C2cis

Charge: 0

C	-2.978647	-0.538013	-1.202000
C	-2.810310	-1.563225	-0.099598
C	-1.878049	-1.293796	1.047113
C	-1.537545	-2.361538	0.034832
H	-0.738228	-2.138872	-0.664011
H	-1.598878	-3.397136	0.347894
C	-1.121071	-0.003391	1.151121
C	-1.949644	1.267521	1.245413
C	-2.606495	1.736856	-0.056503
C	-3.601253	0.776487	-0.714781
H	-4.048897	1.295908	-1.568626
H	-4.421884	0.556676	-0.020348
H	-3.117056	2.682864	0.151457
H	-1.812805	1.966514	-0.778444
H	-1.313404	2.073320	1.623064
H	-2.723612	1.091772	2.005497
H	-0.470375	0.109590	0.177985
H	-0.364051	-0.053657	1.937159
H	-2.195061	-1.661388	2.020462
H	-3.724638	-2.083980	0.169824
H	-2.009911	-0.324096	-1.667096
H	-3.604886	-0.974699	-1.986598
C	2.381836	-0.842567	0.060833
C	1.767581	0.503767	-0.390680
C	1.995087	1.630083	0.594632
H	1.591162	1.315828	1.561042
H	3.075623	1.742871	0.720342
C	1.362834	2.942326	0.151928
H	0.283181	2.840616	0.023346
H	1.542155	3.706237	0.910904
H	1.790960	3.293833	-0.789024
F	2.108529	-1.829028	-0.796562

F 1.930706 -1.222138 1.265750

F 3.718244 -0.740105 0.145015

O 2.018639 0.737418 -1.715966

O 0.468530 0.331604 -0.863598

1 imaginary frequency: –923.06 cm^{-1}

S4-TS1C2trans

Charge: 0

C 3.717515 0.199408 -1.027741

C 3.010278 1.343928 -0.335078

C 1.706087 1.092159 0.400138

C 1.722178 1.928429 -0.849615

H 1.302037 1.477279 -1.743418

H 1.557848 2.995456 -0.753471

C 1.162918 -0.298347 0.396508

C 1.958779 -1.309369 1.204331

C 3.148257 -1.872589 0.408728

C 4.214998 -0.870742 -0.043594

H 5.021351 -1.439668 -0.518121

H 4.656978 -0.378566 0.831699

H 3.628215 -2.637449 1.028513

H 2.756639 -2.389644 -0.476190

H 1.312878 -2.146516 1.485562

H 2.309481 -0.842958 2.131394

H 0.947259 -0.665555 -0.610416

H 1.601358 1.616981 1.344504

H 3.676142 2.035273 0.172835

H 3.054169 -0.266572 -1.765131

H 4.566402 0.604398 -1.587806

H 0.103157 -0.236171 0.885284

C -2.475061 -0.920022 -0.566651

C -2.148955 0.273346 0.360619

C	-1.870802	1.553999	-0.394384
H	-1.032880	1.364032	-1.070330
H	-2.744821	1.764159	-1.017384
C	-1.563755	2.732970	0.517709
H	-0.693504	2.533615	1.146040
H	-1.346343	3.614010	-0.089227
H	-2.411728	2.968572	1.164146
F	-2.607955	-2.061768	0.112783
F	-1.523010	-1.110883	-1.491819
F	-3.629649	-0.700571	-1.216881
O	-2.992855	0.309361	1.436488
O	-1.246364	-0.088199	1.360883

1 imaginary frequency: –1000.84 cm^{-1}

S4-TS1C3cis

Charge: 0

C	4.105606	0.740434	0.171431
C	3.890250	-0.537899	-0.613714
C	2.639181	-1.352788	-0.411936
C	3.972401	-1.890074	0.046843
H	4.154314	-1.905318	1.117024
H	4.412276	-2.714432	-0.502885
C	1.589747	-0.895685	0.583964
C	0.980150	0.422625	0.135260
C	1.730673	1.689974	0.497119
C	3.100530	1.840833	-0.185821
H	3.503970	2.812754	0.115949
H	2.969245	1.879170	-1.273699
H	1.111763	2.553482	0.234646
H	1.878331	1.714056	1.583010
H	0.682385	0.398877	-0.917957
H	2.025100	-0.773500	1.580091

H	0.813406	-1.661686	0.664641
H	2.226175	-1.801639	-1.310088
H	4.244182	-0.491008	-1.639601
H	4.051263	0.538354	1.246996
H	5.121662	1.099537	-0.022146
H	-0.045547	0.514435	0.692404
C	-2.417533	-1.135626	-0.146399
C	-2.341095	0.370442	0.196867
C	-2.342681	1.260821	-1.026593
H	-1.554289	0.913506	-1.699411
H	-3.295820	1.099775	-1.538812
C	-2.146199	2.732927	-0.693947
H	-1.169864	2.907354	-0.236547
H	-2.199334	3.322810	-1.610961
H	-2.918494	3.096713	-0.012990
F	-2.305138	-1.904037	0.939511
F	-1.452367	-1.504531	-1.001749
F	-3.596540	-1.419125	-0.723005
O	-3.172120	0.685938	1.235603
O	-1.376693	0.622824	1.174282

1 imaginary frequency: –959.56 cm^{-1}

S4-TS1C3trans

Charge: 0

C	-3.655997	-1.150036	-0.401795
C	-3.392905	0.264440	-0.873674
C	-2.510560	1.178970	-0.068215
C	-3.988931	1.458877	-0.172721
H	-4.589956	1.262262	0.709921
H	-4.315072	2.304138	-0.767922
C	-1.887995	0.698484	1.225779
C	-0.916163	-0.457895	1.048627

C	-1.479597	-1.842474	0.787869
C	-2.393706	-2.020796	-0.426611
H	-2.680669	-3.076325	-0.468005
H	-1.828997	-1.816286	-1.344455
H	-0.643576	-2.545195	0.716510
H	-2.038665	-2.121140	1.693380
H	-0.180897	-0.490647	1.855936
H	-0.263359	-0.185853	0.110897
H	-2.667264	0.366145	1.923015
H	-1.371444	1.533055	1.708722
H	-1.862945	1.827985	-0.649836
H	-3.281973	0.355797	-1.950535
H	-4.069418	-1.140937	0.613132
H	-4.423520	-1.596547	-1.042247
C	2.677522	-0.742145	0.017048
C	1.903608	0.539375	-0.371595
C	1.962054	1.618465	0.688280
H	1.557934	1.198419	1.613436
H	3.017023	1.841324	0.871660
C	1.204874	2.879959	0.298365
H	0.147160	2.672143	0.124636
H	1.276987	3.610565	1.106284
H	1.621029	3.332126	-0.604174
F	2.514367	-1.717486	-0.880248
F	2.287462	-1.222650	1.207266
F	3.992489	-0.483480	0.097762
O	2.142519	0.882161	-1.673732
O	0.646923	0.234111	-0.895208

1 imaginary frequency: –930.83 cm^{-1}

S4

Charge: 0

C	-0.116135	-1.512707	0.347868
C	-1.181094	-0.752511	-0.415181
C	-1.181095	0.752488	-0.415183
C	-2.279803	-0.000010	0.294476
H	-2.254251	-0.000014	1.380209
H	-3.274659	0.000003	-0.136752
C	-0.116158	1.512684	0.347869
C	1.289928	1.306435	-0.230699
C	1.966780	0.000030	0.198271
C	1.289986	-1.306418	-0.230663
H	1.932048	-2.135740	0.086033
H	1.240126	-1.355573	-1.326069
H	2.992197	0.000064	-0.188552
H	2.052066	0.000041	1.293243
H	1.931993	2.135794	0.085898
H	1.239983	1.355543	-1.326106
H	-0.118889	1.212035	1.401734
H	-0.368108	2.578546	0.333272
H	-1.460014	1.212834	-1.359123
H	-1.460020	-1.212832	-1.359126
H	-0.118893	-1.212078	1.401782
H	-0.368027	-2.578575	0.333012

There are no imaginary frequencies

S5-TS1C2cis

Charge: 0

C	-2.782787	1.556067	-0.576485
C	-2.881784	1.400630	0.953866
H	-3.636049	0.644282	1.195906
H	-3.246840	2.336969	1.386293
C	-1.573453	1.063135	1.668390
C	-0.898317	-0.230071	1.257274

C	-1.792211	-1.420359	1.099875
C	-1.426983	-2.610052	0.255684
C	-2.584756	-1.725343	-0.144788
C	-2.508122	-0.898681	-1.410613
C	-3.333474	0.391294	-1.409264
H	-3.415252	0.732930	-2.447031
H	-4.356860	0.168694	-1.080071
H	-2.860465	-1.533310	-2.232139
H	-1.466880	-0.660867	-1.643299
H	-3.572733	-2.134375	0.049034
H	-0.523494	-2.526552	-0.340682
H	-1.629364	-3.601372	0.644095
H	-2.309148	-1.645048	2.032096
H	-0.042921	-0.448780	1.901155
H	-0.358784	-0.033589	0.222337
H	-0.860767	1.887297	1.558157
H	-1.770625	0.980582	2.746181
H	-1.740876	1.743264	-0.863536
H	-3.338685	2.452299	-0.868748
C	2.616073	-0.746563	0.056498
C	1.856728	0.517790	-0.411597
C	2.017087	1.696156	0.525176
H	1.573717	1.415212	1.484974
H	3.086798	1.845036	0.697510
C	1.375957	2.968582	-0.011256
H	0.313524	2.822364	-0.219547
H	1.468287	3.763827	0.731006
H	1.863757	3.303559	-0.928789
F	2.306572	-1.817405	-0.678846
F	2.362120	-1.050964	1.337554
F	3.940933	-0.551946	-0.052887
O	2.045819	0.719000	-1.752473
O	0.558342	0.217863	-0.821758

1 imaginary frequency: –941.43 cm^{-1}

S5-TS1C2trans

Charge: 0

C	3.179155	-1.752757	-0.460728
C	2.829576	-1.659497	1.037139
H	3.488013	-0.929563	1.519926
H	3.035728	-2.620453	1.518020
C	1.367476	-1.307977	1.351662
C	0.912597	-0.023438	0.685105
C	1.739983	1.177326	0.966280
C	1.645900	2.409421	0.100109
C	2.860677	1.536324	-0.003503
C	3.132337	0.739342	-1.261302
C	3.928790	-0.554834	-1.062089
H	4.308657	-0.861868	-2.042629
H	4.812356	-0.342861	-0.446211
H	3.697003	1.390140	-1.938946
H	2.193113	0.514603	-1.778094
H	3.756894	1.904580	0.488105
H	0.942518	2.369218	-0.726124
H	1.729381	3.374800	0.585196
H	1.969987	1.339078	2.014890
H	-0.157477	0.191060	1.100947
H	0.729441	-0.153134	-0.382739
H	0.715289	-2.124730	1.025954
H	1.253873	-1.216319	2.436971
H	2.268300	-1.938800	-1.043916
H	3.813229	-2.632042	-0.610443
C	-2.429049	-1.084942	-0.453816
C	-2.326968	0.327937	0.166969
C	-2.020502	1.408427	-0.846488
H	-1.062470	1.161243	-1.312789
H	-2.782143	1.352913	-1.629671

C	-1.974172	2.801722	-0.235813
H	-1.225325	2.863700	0.556511
H	-1.708613	3.527562	-1.006891
H	-2.942682	3.087819	0.179177
F	-2.519387	-2.036459	0.478345
F	-1.371133	-1.377615	-1.224872
F	-3.524769	-1.169198	-1.225659
O	-3.324244	0.537748	1.078811
O	-1.572290	0.323523	1.338849

1 imaginary frequency: –1033.08 cm^{-1}

S5-TS1C3cis

Charge: 0

C	1.894554	1.814743	-0.019932
C	1.276879	0.869974	-1.065426
H	2.014583	0.652351	-1.849090
H	0.449564	1.374203	-1.573286
C	0.754324	-0.454713	-0.547169
C	1.678366	-1.311460	0.299592
C	2.988831	-1.554490	-0.418215
C	4.216877	-1.993255	0.337302
C	4.150997	-0.608050	-0.258761
C	4.103488	0.600972	0.652260
C	3.426090	1.839516	0.058352
H	3.710667	2.702242	0.670518
H	3.831550	2.029149	-0.943918
H	5.136960	0.860965	0.909045
H	3.617463	0.342267	1.599319
H	4.739597	-0.453042	-1.159209
H	4.132811	-2.062084	1.417894
H	4.864649	-2.733955	-0.117503
H	2.873423	-1.980336	-1.411315

H	1.185421	-2.263870	0.515872
H	1.865314	-0.826357	1.261216
H	0.278662	-1.030917	-1.345568
H	1.484060	1.586177	0.971481
H	1.566066	2.832413	-0.251476
H	-0.146645	-0.199509	0.158211
C	-3.036140	-0.946581	-0.142308
C	-2.412529	0.373409	0.367245
C	-2.422011	1.474595	-0.669319
H	-1.858435	1.116848	-1.535571
H	-3.457042	1.611593	-0.995539
C	-1.841520	2.783722	-0.154268
H	-0.813858	2.656007	0.191170
H	-1.837659	3.521163	-0.959266
H	-2.435237	3.185279	0.669440
F	-2.894344	-1.941649	0.736640
F	-2.485472	-1.348470	-1.297522
F	-4.351618	-0.783309	-0.357958
O	-2.825956	0.658218	1.637860
O	-1.210073	0.143031	1.038517

1 imaginary frequency: –966.30 cm^{-1}

S5-TS1C3trans

Charge: 0

C	2.504259	2.080158	-0.503465
C	1.070750	1.543781	-0.280657
H	0.865104	0.751814	-1.005321
H	0.352649	2.344122	-0.476843
C	0.821488	1.039389	1.127489
C	1.679037	-0.095300	1.653120
C	1.823948	-1.230298	0.671579
C	2.974713	-2.198294	0.759554

C	2.900214	-1.217008	-0.384461
C	3.925304	-0.106899	-0.492218
C	3.460524	1.141712	-1.247820
H	4.350070	1.723531	-1.513610
H	2.997907	0.840167	-2.196381
H	4.801898	-0.516116	-1.007743
H	4.274653	0.182856	0.504966
H	2.603336	-1.632041	-1.344204
H	3.726006	-2.004818	1.519761
H	2.782685	-3.247487	0.566317
H	0.877210	-1.660120	0.358456
H	1.252478	-0.457005	2.594577
H	2.663107	0.314407	1.909716
H	0.759508	1.860383	1.847889
H	2.949532	2.365874	0.457155
H	2.428519	3.002580	-1.086689
H	-0.281151	0.635334	1.139018
C	-2.077152	-1.120672	-0.621941
C	-2.398833	0.137345	0.217750
C	-2.523918	1.394062	-0.615637
H	-1.663958	1.449035	-1.287405
H	-3.412101	1.269858	-1.242288
C	-2.624337	2.659271	0.223881
H	-1.710129	2.825437	0.798028
H	-2.773206	3.519481	-0.431570
H	-3.466282	2.613817	0.918103
F	-1.887301	-2.199757	0.140934
F	-0.979536	-0.961474	-1.374752
F	-3.098290	-1.388258	-1.453249
O	-3.371327	-0.128897	1.141015
O	-1.617133	0.203999	1.373813

1 imaginary frequency: –978.83 cm^{-1}

S5

Charge: 0

C	1.912704	0.630191	-0.261988
C	1.722515	-0.629600	0.605287
H	1.182742	-0.359121	1.519602
H	2.702956	-0.990390	0.932667
C	1.010748	-1.800918	-0.079932
C	-0.376644	-1.480413	-0.643419
C	-1.271548	-0.810456	0.371789
C	-2.500319	-0.043800	-0.046315
C	-1.306087	0.690740	0.513242
C	-0.500742	1.613921	-0.377691
C	0.964373	1.801107	0.028645
H	1.347769	2.681354	-0.499638
H	1.014561	2.042401	1.098653
H	-0.991135	2.594632	-0.367928
H	-0.545222	1.269085	-1.416598
H	-1.411638	1.053118	1.532777
H	-2.679194	0.057499	-1.113102
H	-3.396600	-0.127963	0.557992
H	-1.362697	-1.358311	1.306937
H	-0.842626	-2.410206	-0.989461
H	-0.281475	-0.843188	-1.527858
H	1.642929	-2.177971	-0.892709
H	0.918826	-2.619451	0.644338
H	1.837725	0.363601	-1.324146
H	2.933073	1.000283	-0.119230

There are no imaginary frequencies

S7-Int1C4

Charge: 0

C	-2.468706	1.949323	0.359217

C	-2.209955	1.399404	-1.042087
C	-1.829497	-0.018720	-1.095083
C	-1.920278	-0.872284	-0.012098
C	-2.287615	-0.348248	1.355296
C	-3.147155	0.909731	1.250025
H	-1.541126	-0.423663	-2.059776
H	-3.110434	1.462216	-1.673948
H	-1.456007	1.969694	-1.594970
H	-1.518267	2.245323	0.811945
H	-2.799606	-1.133447	1.916168
H	-1.357614	-0.127319	1.889300
H	-4.125807	0.645912	0.832435
H	-3.319349	1.322383	2.246261
H	1.427563	-0.121812	-2.176575
C	-2.857442	-2.079499	-0.649413
C	-1.501556	-2.330984	-0.195598
H	-3.078476	-2.047999	-1.707449
H	-3.699656	-2.220740	0.016758
H	-0.740808	-2.514506	-0.943896
H	-1.365913	-2.828959	0.756889
C	2.262958	-0.867434	0.296015
C	2.002501	0.496656	-0.418944
C	1.347847	1.455839	0.595098
H	0.391608	1.035386	0.919505
H	1.993400	1.508158	1.477386
C	1.137431	2.854557	0.031617
H	0.506292	2.837057	-0.860714
H	0.649903	3.492585	0.773400
H	2.088219	3.321849	-0.236543
F	2.654184	-1.824003	-0.572915
F	1.186286	-1.365996	0.943341
F	3.241831	-0.774090	1.222009
O	3.127946	0.921689	-0.987757
O	0.964873	0.194491	-1.393518
H	-3.080727	2.849968	0.281536

There are no imaginary frequencies

S7-TS1C4ax

Charge: 0

C	-2.539391	-1.988744	-0.839632
C	-1.848260	-1.910487	0.522025
C	-1.230771	-0.543686	0.761921
C	-2.154154	0.599412	0.488664
C	-2.857608	0.514935	-0.851059
C	-3.537138	-0.844497	-1.028665
H	-0.721434	-0.460143	1.724325
H	-2.584574	-2.080454	1.319439
H	-1.086952	-2.688659	0.620636
H	-1.779422	-1.937549	-1.629076
H	-3.585021	1.327355	-0.938684
H	-2.116770	0.657540	-1.648704
H	-4.344535	-0.941859	-0.292181
H	-3.997548	-0.906204	-2.019001
H	-0.365320	-0.456348	-0.022665
C	-2.944887	1.113699	1.680468
C	-1.846809	1.946878	1.078213
H	-2.763676	0.653605	2.645145
H	-3.973416	1.400970	1.491010
H	-0.929326	2.051293	1.646504
H	-2.127698	2.816203	0.494060
H	-3.042680	-2.954018	-0.944219
C	2.608798	-0.535232	0.491595
C	1.815778	0.254947	-0.574240
C	1.619588	1.710447	-0.215390
H	1.159700	1.751588	0.775019
H	2.612854	2.159724	-0.125616
C	0.774431	2.467149	-1.230018

H	-0.221000	2.028977	-1.323554
H	0.657432	3.502848	-0.905123
H	1.245099	2.472763	-2.215431
F	2.698209	-1.833020	0.190973
F	2.044084	-0.442355	1.704965
F	3.859269	-0.056540	0.591771
O	2.228850	-0.060180	-1.838761
O	0.695247	-0.454735	-1.004309

1 imaginary frequency: –1009.76 cm^{-1}

S7-TS1C4eq

Charge: 0

C	3.236362	1.675463	-0.692892
C	1.897463	1.588592	0.059744
C	1.254846	0.247236	-0.219417
C	2.134691	-0.922466	0.121952
C	3.467402	-0.836456	-0.610883
C	4.151407	0.503595	-0.336418
H	0.294819	0.169516	0.452333
H	2.080726	1.689784	1.135216
H	1.234011	2.401384	-0.246077
H	3.041336	1.669137	-1.772123
H	4.103146	-1.671621	-0.301589
H	3.290477	-0.943097	-1.688602
H	4.417858	0.561098	0.725988
H	5.083557	0.569604	-0.905476
H	0.855711	0.181285	-1.236904
C	2.127977	-1.451138	1.530265
C	1.525128	-2.261444	0.401226
H	1.448235	-0.996122	2.242394
H	3.072120	-1.786162	1.945483
H	0.444436	-2.343711	0.363356

H	2.065459	-3.140082	0.065929
H	3.720527	2.627777	-0.458789
C	-2.366677	-0.823860	-0.537424
C	-2.020311	0.402968	0.339088
C	-1.997492	1.703499	-0.435731
H	-1.235632	1.610774	-1.215081
H	-2.961681	1.806179	-0.941324
C	-1.719723	2.913925	0.444636
H	-0.766274	2.815702	0.967758
H	-1.673625	3.811281	-0.175375
H	-2.508108	3.055657	1.186567
F	-2.275604	-1.968353	0.144303
F	-1.559500	-0.925486	-1.603643
F	-3.626049	-0.725963	-0.993424
O	-2.698931	0.360475	1.527303
O	-0.926466	0.145817	1.165987

1 imaginary frequency: –895.44 cm^{-1}

S7-TS1C5ax

Charge: 0

C	-1.847452	2.102535	0.479557
C	-1.018046	0.944920	0.996883
C	-1.812676	-0.312031	1.290785
C	-2.707381	-0.683433	0.125839
C	-3.583041	0.461656	-0.345070
C	-2.736017	1.687555	-0.694314
H	-1.147392	-1.139082	1.550915
H	-0.289621	0.669841	0.117327
H	-0.336399	1.225375	1.802962
H	-2.476606	2.449545	1.311626
H	-4.174004	0.147438	-1.210758
H	-4.290879	0.724217	0.452198

H	-2.101711	1.452049	-1.557345
H	-3.378291	2.522643	-0.987184
H	-2.430857	-0.105717	2.177129
C	-2.142935	-1.615794	-0.913785
C	-3.222956	-2.088773	0.034615
H	-1.138234	-1.994743	-0.763888
H	-2.417230	-1.443720	-1.949195
H	-2.937572	-2.785870	0.815557
H	-4.219831	-2.235734	-0.367417
H	-1.198930	2.938451	0.204038
C	2.096690	-1.147266	0.178911
C	1.957372	0.281734	-0.394937
C	2.533616	1.346695	0.512737
H	2.132097	1.194549	1.518039
H	3.611652	1.169988	0.567342
C	2.238244	2.760082	0.031225
H	1.164972	2.962549	0.035852
H	2.721159	3.479338	0.695405
H	2.617584	2.925849	-0.979363
F	1.541678	-2.064694	-0.616382
F	1.520433	-1.259301	1.385207
F	3.393077	-1.466803	0.321293
O	2.278446	0.317660	-1.723771
O	0.679167	0.498956	-0.908602

1 imaginary frequency: –949.11 cm^{-1}

S7-TS1C5eq

Charge: 0

C	-1.248784	-1.715751	-0.445785
C	-0.953603	-0.249562	-0.211924
C	-2.028673	0.677808	-0.741890
C	-3.351915	0.297849	-0.090661

C	-3.712673	-1.160973	-0.292813
C	-2.593078	-2.074494	0.209533
H	-1.784802	1.721846	-0.529052
H	-0.705891	-0.035815	0.832291
H	0.033834	0.010698	-0.783544
H	-1.311810	-1.907450	-1.522992
H	-4.649361	-1.387909	0.225504
H	-3.880319	-1.345173	-1.361878
H	-2.501906	-1.971760	1.297204
H	-2.830280	-3.121803	0.002096
H	-2.113458	0.563435	-1.828793
C	-3.688406	0.936781	1.230414
C	-4.439053	1.329447	-0.023372
H	-2.985279	1.653112	1.641960
H	-4.211176	0.334103	1.965361
H	-4.237823	2.307555	-0.447060
H	-5.463464	0.987539	-0.126901
H	-0.448282	-2.339401	-0.041430
C	2.710883	-0.780770	0.337615
C	2.250329	0.566902	-0.265047
C	1.940179	1.613589	0.783459
F	2.875235	-1.718477	-0.598627
F	1.831335	-1.255979	1.231661
F	3.886098	-0.629537	0.969290
O	3.030402	0.924768	-1.330519
O	1.310027	0.374087	-1.278185
H	1.259681	1.171991	1.515951
H	2.876882	1.830367	1.305303
C	1.338506	2.883312	0.198145
H	1.188859	3.615040	0.994324
H	1.995611	3.329489	-0.551367
H	0.369283	2.684410	-0.264299

1 imaginary frequency: –927.07 cm^{-1}

S7-TS1C6ax

Charge: 0

C	0.811118	-0.702656	-0.922862
C	1.539845	0.552934	-1.354664
C	2.444461	1.094401	-0.246524
C	3.354619	0.005941	0.287975
C	2.598477	-1.212229	0.780952
C	1.695864	-1.774496	-0.318933
H	3.036021	1.935292	-0.619979
H	2.151570	0.290468	-2.229152
H	0.826055	1.312611	-1.684453
H	0.085968	-0.373246	-0.057225
H	3.298059	-1.980280	1.123225
H	1.981378	-0.928910	1.643132
H	2.318008	-2.178058	-1.130144
H	1.088070	-2.601597	0.056802
H	1.820979	1.475396	0.572456
C	4.669051	-0.218526	-0.412124
C	4.631184	0.395694	0.971393
H	4.918477	0.429664	-1.245626
H	5.029637	-1.237869	-0.501784
H	4.856827	1.453286	1.059635
H	4.968179	-0.214439	1.802881
H	0.128317	-1.085864	-1.683124
C	-2.887322	-0.722314	-0.081159
C	-2.045758	0.483374	0.398100
C	-2.000436	1.615359	-0.604306
H	-1.555896	1.222030	-1.523120
H	-3.032090	1.891713	-0.839493
C	-1.219597	2.823133	-0.106091
H	-0.189003	2.558524	0.139366
H	-1.194946	3.586494	-0.886178
H	-1.684433	3.260158	0.779988

F	-2.817667	-1.750971	0.767314
F	-2.490628	-1.165585	-1.283613
F	-4.179253	-0.372302	-0.186295
O	-2.299482	0.772610	1.709154
O	-0.827052	0.069973	0.940296

1 imaginary frequency: –957.23 cm^{-1}

S7-TS1C6eq

Charge: 0

C	0.880927	-0.705663	0.706166
C	1.814188	0.092955	1.591755
C	3.263400	-0.365795	1.356484
C	3.612485	-0.287659	-0.115629
C	2.660289	-1.073561	-0.993568
C	1.210471	-0.608542	-0.767824
H	3.946176	0.252953	1.946169
H	1.731680	1.156507	1.340117
H	1.543586	-0.024457	2.643778
H	0.774056	-1.743006	1.039764
H	2.923639	-0.953424	-2.048550
H	2.740531	-2.141417	-0.755555
H	1.120304	0.431708	-1.098559
H	0.520835	-1.211352	-1.362363
H	3.376632	-1.400214	1.703850
C	4.135957	1.021627	-0.644434
C	5.053530	-0.175416	-0.518787
H	4.269573	1.836143	0.059624
H	3.833554	1.323388	-1.641700
H	5.799760	-0.158194	0.268521
H	5.364367	-0.671078	-1.432381
H	-0.193053	-0.271786	0.882851
C	-2.957690	-0.727054	-0.231909

C	-2.262506	0.590167	0.181149
C	-1.733815	1.381091	-0.995678
H	-1.171293	0.702450	-1.641860
H	-2.603504	1.719454	-1.566461
C	-0.868515	2.561659	-0.577972
H	0.026307	2.230059	-0.046384
H	-0.550211	3.111255	-1.465904
H	-1.418052	3.250845	0.066929
F	-3.420411	-1.402656	0.822830
F	-2.121475	-1.540844	-0.893743
F	-4.000102	-0.474954	-1.039478
O	-2.977240	1.257037	1.136172
O	-1.398926	0.380886	1.259216

1 imaginary frequency: –964.23 cm^{-1}

S7-TS2'C4

Charge: 0

C	-3.083646	1.558411	0.044222
C	-3.333897	0.506289	-1.037614
C	-2.312121	-0.620558	-0.996433
C	-1.326586	-0.687785	0.085908
C	-1.483766	0.107616	1.334816
C	-2.793553	0.901556	1.389442
H	-1.891105	-0.881830	-1.965511
H	-4.328863	0.070497	-0.904007
H	-3.321276	0.969755	-2.025489
H	-2.228674	2.182954	-0.240100
H	-1.348576	-0.544723	2.199467
H	-0.645140	0.807595	1.336024
H	-3.617406	0.232759	1.660022
H	-2.710450	1.649667	2.179926
H	0.448135	-0.198333	-1.690425

C	-2.602470	-2.034480	-0.301464
C	-1.083265	-2.134134	0.098513
H	-2.905177	-2.778855	-1.032467
H	-3.283872	-1.974819	0.542760
H	-0.523266	-2.519090	-0.749322
H	-0.797319	-2.579335	1.045660
C	2.291354	-0.723738	0.244959
C	1.820913	0.559297	-0.508445
C	1.799109	1.738710	0.482468
H	1.309579	1.447507	1.416443
H	2.842202	1.956840	0.728458
C	1.127831	2.980931	-0.088390
H	0.058298	2.819386	-0.245673
H	1.241711	3.821305	0.600945
H	1.573767	3.269536	-1.043896
F	2.242643	-1.817039	-0.541329
F	1.561147	-1.016542	1.343539
F	3.569007	-0.608097	0.662985
O	2.540313	0.739630	-1.602320
O	0.410267	0.272212	-0.848674
H	-3.951792	2.216845	0.120488

1 imaginary frequency: –185.86 cm^{-1}

S7-TS2C4

Charge: 0

C	-2.276504	-2.077064	-0.795352
C	-1.598372	-1.836740	0.550952
C	-1.401911	-0.415824	0.885857
C	-2.138328	0.607059	0.298983
C	-2.932062	0.344452	-0.957445
C	-3.428421	-1.098477	-1.018673
H	-0.755419	-0.176506	1.725753

H	-2.222579	-2.213868	1.377884
H	-0.645462	-2.359470	0.653104
H	-1.536479	-1.955177	-1.592429
H	-3.761325	1.053049	-1.017041
H	-2.268651	0.552330	-1.805091
H	-4.194823	-1.249689	-0.249733
H	-3.899275	-1.283767	-1.986539
H	0.141938	-0.042268	-1.586718
C	-2.948000	1.120511	1.625208
C	-2.045880	2.001989	0.879487
H	-2.652227	0.758540	2.601236
H	-4.008480	1.093809	1.406143
H	-1.113600	2.274024	1.358452
H	-2.486935	2.752651	0.234965
H	-2.632191	-3.108332	-0.838844
C	2.527048	-0.588510	0.422980
C	1.822803	0.307397	-0.639487
C	1.534522	1.686745	-0.007155
H	0.970529	1.551055	0.920387
H	2.492464	2.144014	0.258580
C	0.772216	2.610329	-0.949576
H	-0.186649	2.179108	-1.252584
H	0.561845	3.563784	-0.458432
H	1.350287	2.819231	-1.852997
F	2.760619	-1.834206	-0.031609
F	1.828240	-0.732710	1.572773
F	3.726265	-0.079631	0.773427
O	2.529369	0.342924	-1.765091
O	0.556662	-0.406022	-0.797668

1 imaginary frequency: –47.57 cm^{-1}

S7

Charge: 0

C	2.105522	0.000010	-0.037656
C	1.299593	1.264592	0.268084
C	-0.034313	1.265685	-0.482934
C	-0.817844	-0.000005	-0.191549
C	-0.034301	-1.265695	-0.482916
C	1.299614	-1.264587	0.268087
H	-0.627520	2.145320	-0.212882
H	1.102774	1.314687	1.346648
H	1.879161	2.155816	0.008010
H	2.382579	0.000008	-1.100127
H	-0.627505	-2.145330	-0.212864
H	0.158867	-1.326260	-1.562495
H	1.102816	-1.314699	1.346653
H	1.879188	-2.155799	0.007989
H	0.158861	1.326252	-1.562507
C	-1.729141	0.000010	1.007615
C	-2.304161	-0.000012	-0.393599
H	-1.809967	0.914501	1.585948
H	-1.809962	-0.914468	1.585968
H	-2.769169	0.914472	-0.747278
H	-2.769159	-0.914512	-0.747253
H	3.039221	0.000023	0.533401

There are no imaginary frequencies

S8-Int1

Charge: 0

C	-2.024019	-0.119788	-0.085125
C	-1.739540	0.639249	1.217167
C	-0.698678	1.699007	1.075393
C	-0.267671	2.179433	-0.155158
C	-0.827607	1.641229	-1.438087

C	-2.138067	0.886511	-1.235403
H	-0.159441	1.990293	1.969605
H	-2.643481	1.120031	1.610591
H	-1.390489	-0.032632	2.003536
H	-1.141848	-0.741222	-0.281621
H	-0.940049	2.458074	-2.154473
H	-0.055680	0.973622	-1.835752
H	-2.933698	1.607834	-1.018535
H	-2.401456	0.389548	-2.170222
H	1.772451	-0.542344	2.099297
C	-1.032028	3.527770	0.504980
C	0.362800	3.512752	-0.074028
H	-1.127909	3.807033	1.546355
H	-1.891932	3.727518	-0.122276
H	1.145184	3.658083	0.661950
H	0.514833	4.002695	-1.027629
C	3.160389	-0.001162	-0.189984
C	2.325150	-1.202651	0.355935
C	1.519689	-1.805347	-0.812881
H	0.872666	-1.037166	-1.246522
H	2.230367	-2.104733	-1.589199
C	0.685047	-3.006185	-0.388402
H	-0.046707	-2.733497	0.376535
H	0.136872	-3.408846	-1.244236
H	1.314318	-3.804686	0.012719
F	3.788685	0.667783	0.799635
F	2.429987	0.921031	-0.858145
F	4.123852	-0.403179	-1.046770
O	3.128328	-2.045069	1.002389
O	1.348518	-0.581740	1.235874
C	-3.225064	-1.099371	0.024757
C	-4.567114	-0.357408	0.043720
H	-4.758413	0.156200	-0.902568
H	-5.383690	-1.068279	0.205421
H	-4.610795	0.383971	0.847877

C	-3.197101	-2.059719	-1.172780
H	-2.259805	-2.625471	-1.195122
H	-4.021912	-2.775774	-1.101735
H	-3.299610	-1.534710	-2.126174
C	-3.101301	-1.940170	1.303634
H	-3.853542	-2.735028	1.302102
H	-2.114669	-2.410310	1.374126
H	-3.258360	-1.341343	2.204935

There are no imaginary frequencies

S8-Int1a

Charge: 0

C	2.264029	-0.367325	0.217040
C	1.462003	-0.178020	-1.083233
C	0.519800	0.983906	-1.035158
C	0.917014	2.223243	-0.360330
C	1.751251	2.015732	0.889330
C	2.852368	0.984125	0.647295
H	-0.238964	1.052456	-1.812210
H	2.164463	-0.020048	-1.919837
H	0.901006	-1.082820	-1.331638
H	1.544626	-0.665638	0.993867
H	2.179875	2.969144	1.212631
H	1.099071	1.664742	1.699791
H	3.525095	1.358657	-0.134205
H	3.448936	0.872861	1.555964
H	-0.673538	0.026092	0.370888
C	1.276171	3.413938	-1.262751
C	0.018567	3.428549	-0.445250
H	1.186881	3.275230	-2.333956
H	2.108156	4.031692	-0.941503
H	-0.926492	3.311478	-0.964807

H	-0.014188	4.075444	0.424711
C	-3.121088	-1.228525	-0.539261
C	-2.569234	-0.346493	0.616970
C	-2.840256	1.133838	0.375807
H	-2.361261	1.426433	-0.562814
H	-3.918573	1.254038	0.243897
C	-2.347315	2.008282	1.522462
H	-1.267304	1.921232	1.661023
H	-2.571875	3.054399	1.306587
H	-2.839458	1.742434	2.460888
F	-2.874248	-2.525179	-0.329012
F	-2.542241	-0.891877	-1.699366
F	-4.446422	-1.088722	-0.688646
O	-3.164702	-0.821493	1.796975
O	-1.220745	-0.669531	0.790448
C	3.307316	-1.519838	0.144850
C	4.497528	-1.168058	-0.757176
H	5.174765	-2.025213	-0.832870
H	4.177728	-0.909893	-1.771288
H	5.072361	-0.327497	-0.358473
C	3.829635	-1.822056	1.557213
H	3.006265	-2.086181	2.229575
H	4.525839	-2.666555	1.530752
H	4.362015	-0.971855	1.991418
C	2.641288	-2.796826	-0.388340
H	3.322646	-3.647421	-0.285328
H	1.727526	-3.027676	0.170363
H	2.379713	-2.712176	-1.446401

There are no imaginary frequencies

S8-TS1ax

Charge: 0

C	2.173547	-0.182957	0.277718
C	1.273053	-0.022858	-0.955357
C	0.191093	1.017130	-0.737791
C	0.670432	2.327121	-0.208997
C	1.609583	2.191729	0.970033
C	2.730719	1.193807	0.672534
H	-0.484181	1.121170	-1.590497
H	1.871189	0.298467	-1.818947
H	0.801113	-0.967978	-1.228710
H	1.523193	-0.517498	1.100139
H	2.025082	3.170301	1.228209
H	1.037155	1.845189	1.840250
H	3.347071	1.586818	-0.145053
H	3.377363	1.111592	1.549156
H	-0.493284	0.582310	0.104374
C	0.940033	3.410039	-1.237980
C	-0.238342	3.519510	-0.307559
H	0.747236	3.179797	-2.279593
H	1.797879	4.048376	-1.056413
H	-1.227121	3.362368	-0.725615
H	-0.187040	4.248017	0.494280
C	-2.420786	-1.642333	-0.419383
C	-2.588782	-0.391867	0.474583
C	-3.402827	0.706854	-0.172186
H	-2.889751	0.995147	-1.093934
H	-4.367654	0.280361	-0.461319
C	-3.594659	1.915254	0.733294
H	-2.635713	2.342799	1.033557
H	-4.154365	2.685096	0.198624
H	-4.154388	1.653851	1.633565
F	-1.542017	-2.508996	0.090758
F	-2.004273	-1.327876	-1.654653
F	-3.595422	-2.282007	-0.541887
O	-2.853165	-0.741937	1.769476
O	-1.384144	-0.019603	1.065266

C	3.257065	-1.288368	0.121849
C	4.323316	-0.909080	-0.913769
H	5.036896	-1.730835	-1.034584
H	3.881972	-0.708082	-1.894649
H	4.887970	-0.023971	-0.607500
C	3.941987	-1.527901	1.475476
H	3.207034	-1.788898	2.244672
H	4.654574	-2.355247	1.397157
H	4.495643	-0.650678	1.819542
C	2.596836	-2.608169	-0.305607
H	3.320235	-3.427465	-0.245213
H	1.751584	-2.855465	0.346308
H	2.230589	-2.570772	-1.334858

1 imaginary frequency: –1022.39 cm^{-1}

S8-TS1eq

Charge: 0

C	-2.636649	0.019515	-0.469975
C	-1.312955	-0.325420	0.248496
C	-0.236286	0.656656	-0.152713
C	-0.589419	2.089959	0.113691
C	-1.902275	2.447934	-0.564835
C	-3.010667	1.476455	-0.157814
H	0.712751	0.406799	0.490183
H	-1.461232	-0.280109	1.332960
H	-0.989456	-1.336186	-0.004294
H	-2.437415	-0.040840	-1.550332
H	-2.175439	3.475294	-0.304869
H	-1.764963	2.413223	-1.652908
H	-3.199466	1.585093	0.916687
H	-3.932471	1.752875	-0.675031
H	0.105124	0.499297	-1.180906

C	-0.295194	2.675054	1.467336
C	0.492153	3.115789	0.249963
H	0.209063	2.048584	2.195012
H	-1.016879	3.370667	1.881410
H	1.520128	2.779205	0.167227
H	0.299227	4.107752	-0.143983
C	3.450084	-0.005500	-0.683289
C	2.687865	-0.832648	0.378270
C	2.043169	-2.081553	-0.184858
H	1.325590	-1.765729	-0.947687
H	2.820597	-2.656749	-0.695302
C	1.357789	-2.928061	0.878746
H	0.596987	-2.356617	1.414504
H	0.867117	-3.780164	0.404315
H	2.076354	-3.312697	1.605255
F	3.913070	1.143689	-0.185242
F	2.675845	0.296053	-1.736105
F	4.503932	-0.699117	-1.143134
O	3.412426	-0.931702	1.534738
O	1.884118	-0.015436	1.173692
C	-3.771911	-1.008172	-0.188596
C	-4.980735	-0.686215	-1.080279
H	-5.750041	-1.456132	-0.963538
H	-5.436544	0.273892	-0.825892
H	-4.691977	-0.656706	-2.136505
C	-4.219399	-0.990307	1.278552
H	-4.994218	-1.746892	1.440316
H	-3.392294	-1.215002	1.958341
H	-4.639978	-0.021680	1.562616
C	-3.294590	-2.424359	-0.543583
H	-4.135055	-3.124400	-0.504585
H	-2.876153	-2.457038	-1.555556
H	-2.533212	-2.789033	0.150762

1 imaginary frequency: –904.89 cm^{-1}

S8-TS2'

Charge: 0

C	-2.401217	-0.095703	0.026411
C	-2.219645	1.010874	1.076284
C	-0.864376	1.694098	0.981100
C	0.058921	1.371117	-0.105304
C	-0.417363	0.676244	-1.332423
C	-1.928194	0.431448	-1.332230
H	-0.361162	1.816119	1.938453
H	-2.998053	1.772407	0.962777
H	-2.324289	0.599153	2.080148
H	-1.729001	-0.917970	0.309577
H	-0.083011	1.227331	-2.213726
H	0.103659	-0.283090	-1.346567
H	-2.447834	1.366048	-1.567462
H	-2.153266	-0.270380	-2.136108
H	1.474795	0.209318	1.682340
C	-0.624056	3.097598	0.241320
C	0.823335	2.621050	-0.159130
H	-0.622569	3.917757	0.953567
H	-1.287811	3.272924	-0.600529
H	1.493223	2.795694	0.678156
H	1.242064	2.904540	-1.118807
C	3.429034	0.023544	-0.214799
C	2.480489	-0.990427	0.496451
C	2.027959	-2.049453	-0.526609
H	1.689824	-1.571010	-1.450267
H	2.915655	-2.635297	-0.781769
C	0.937202	-2.966050	0.011149
H	0.005263	-2.421064	0.181805
H	0.729997	-3.765286	-0.704920
H	1.237791	-3.431708	0.953406

F	3.785112	1.038409	0.597273
F	2.896454	0.592429	-1.318715
F	4.574051	-0.569441	-0.612228
O	3.045637	-1.456600	1.595812
O	1.283974	-0.184430	0.822414
C	-3.831746	-0.703238	-0.000748
C	-4.849788	0.260290	-0.623805
H	-4.633122	0.449845	-1.678910
H	-5.854883	-0.169502	-0.564515
H	-4.870848	1.222365	-0.102819
C	-3.810644	-2.004548	-0.815960
H	-3.105087	-2.722731	-0.384784
H	-4.803147	-2.466208	-0.816521
H	-3.527523	-1.835958	-1.858414
C	-4.280643	-1.051328	1.425364
H	-5.217581	-1.616306	1.395860
H	-3.531805	-1.667346	1.935005
H	-4.454334	-0.157060	2.029815

1 imaginary frequency: –186.40 cm^{-1}

S8-TS2

Charge: 0

C	2.218698	-0.178252	0.300230
C	1.296749	-0.137144	-0.923790
C	0.348237	0.986885	-0.944447
C	0.535378	2.154946	-0.214119
C	1.559477	2.184072	0.891599
C	2.722606	1.240148	0.597361
H	-0.455486	0.953347	-1.674900
H	1.871946	-0.009708	-1.856343
H	0.728510	-1.057207	-1.066481
H	1.595188	-0.488209	1.148903

H	1.910181	3.208674	1.035210
H	1.052249	1.884461	1.815615
H	3.282723	1.623368	-0.263220
H	3.402557	1.238425	1.450717
H	-0.916238	0.213946	1.577448
C	0.705647	3.213844	-1.444007
C	-0.379600	3.334776	-0.467179
H	0.481330	2.891041	-2.452031
H	1.637340	3.749450	-1.309112
H	-1.382376	3.112957	-0.810286
H	-0.294761	4.100530	0.294461
C	-2.600830	-1.500426	-0.488898
C	-2.518260	-0.455961	0.664440
C	-3.150182	0.868888	0.182891
H	-2.654619	1.196897	-0.735812
H	-4.198564	0.674825	-0.063528
C	-3.068232	1.965797	1.238013
H	-2.033328	2.174301	1.525905
H	-3.494451	2.895625	0.852892
H	-3.621801	1.691228	2.139052
F	-1.989315	-2.658044	-0.173535
F	-2.047628	-1.087437	-1.652737
F	-3.882289	-1.810683	-0.773239
O	-3.042534	-0.953279	1.779563
O	-1.070309	-0.271990	0.760698
C	3.339773	-1.247528	0.176088
C	4.395943	-0.856322	-0.864895
H	5.130626	-1.661051	-0.969461
H	3.953784	-0.685807	-1.851520
H	4.935914	0.048611	-0.572970
C	4.023470	-1.421605	1.539493
H	3.296122	-1.703553	2.308055
H	4.777775	-2.212631	1.483477
H	4.528419	-0.509349	1.867435
C	2.724473	-2.598299	-0.218350

H	3.478756	-3.388076	-0.148881
H	1.895768	-2.863264	0.447258
H	2.348193	-2.594122	-1.244953

1 imaginary frequency: –54.76 cm^{-1}

S8

Charge: 0

C	0.716158	0.000218	-0.411185
C	-0.068141	1.253337	0.011156
C	-1.486966	1.258364	-0.563073
C	-2.234112	-0.000009	-0.175991
C	-1.486822	-1.258173	-0.563503
C	-0.068177	-1.252900	0.011080
H	-2.032283	2.142730	-0.217452
H	-0.130741	1.293930	1.105717
H	0.449764	2.159490	-0.312054
H	0.748906	0.000404	-1.512180
H	-2.032047	-2.142718	-0.218194
H	-1.433574	-1.320869	-1.658773
H	-0.131312	-1.292992	1.105613
H	0.450074	-2.159127	-0.311442
H	-1.433859	1.321413	-1.658335
C	-2.987907	-0.000272	1.127353
C	-3.733736	-0.000056	-0.191311
H	-2.995918	0.914126	1.711501
H	-2.995871	-0.914877	1.711178
H	-4.239692	0.914434	-0.483471
H	-4.239623	-0.914480	-0.483793
C	2.203622	-0.000069	0.051641
C	2.336637	-0.005290	1.580295
H	3.394651	-0.002493	1.863205
H	1.872805	0.876732	2.031080

H	1.879370	-0.894064	2.024446
C	2.921674	-1.236860	-0.509669
H	2.793268	-1.306299	-1.595533
H	3.994891	-1.177478	-0.301677
H	2.553915	-2.164978	-0.065085
C	2.919810	1.241700	-0.500983
H	3.993639	1.180615	-0.296701
H	2.788141	1.320781	-1.585804
H	2.553253	2.165783	-0.047101

There are no imaginary frequencies

----

cis-P3-OH2

Charge: 0

C	2.813586	1.683827	-0.906701
C	1.751285	1.501432	0.172075
C	1.205525	0.083540	0.136884
C	2.268881	-1.000308	0.135660
C	3.340021	-0.957855	1.192339
C	3.686052	-0.613421	-0.237517
C	4.042320	0.820073	-0.615416
H	4.696306	0.807489	-1.491280
H	4.616287	1.281093	0.196484
H	4.229003	-1.382219	-0.778540
H	3.326173	-0.138208	1.904150
H	3.683260	-1.904528	1.595761
C	1.768132	-2.357874	-0.309627
H	2.574166	-3.095956	-0.289104
H	1.374583	-2.311000	-1.330525
H	0.968067	-2.727557	0.341973
H	0.630432	-0.031799	-0.791892
H	-0.069616	-0.940015	1.228524
H	2.177717	1.709581	1.160804

H	0.919644	2.196611	0.025812
H	3.105492	2.734982	-0.978719
H	2.386915	1.403437	-1.877543
C	-2.682168	-0.880631	-0.327248
C	-2.554141	0.525734	0.330500
C	-2.054899	1.625136	-0.545577
H	-1.090887	1.309990	-0.957014
H	-2.733405	1.673421	-1.407115
C	-1.948099	2.961435	0.166754
H	-1.562361	3.714914	-0.522125
H	-2.921886	3.296222	0.530713
H	-1.266642	2.896413	1.018047
F	-1.681342	-1.145823	-1.175228
F	-3.829540	-0.947560	-1.024444
F	-2.707654	-1.846859	0.590176
O	-2.916380	0.632621	1.473735
O	0.304457	-0.054775	1.245552

There are no imaginary frequencies

----

cis-P3-OH5

Charge: 0

C	2.021279	-2.006207	-0.608895
C	3.128579	-1.888938	0.433143
C	3.962789	-0.635955	0.168632
C	3.135308	0.631441	0.031683
C	2.095955	0.922623	1.074660
C	1.666033	0.497233	-0.313863
C	1.016212	-0.857141	-0.535885
H	0.468031	-0.817151	-1.483985
H	-0.356886	-1.928130	0.357913
H	1.260980	1.295012	-0.927893
H	1.958057	0.211886	1.881677

H	1.926220	1.958132	1.350012
C	3.886738	1.803016	-0.557372
H	3.251304	2.690897	-0.619704
H	4.245969	1.569546	-1.565387
H	4.757347	2.053835	0.058728
H	4.516943	-0.781826	-0.767603
H	4.710375	-0.495080	0.957575
H	2.696494	-1.861859	1.439505
H	3.774255	-2.770326	0.395250
H	1.480451	-2.953549	-0.512466
H	2.471326	-1.997917	-1.608783
C	-3.055320	-0.562443	-0.118414
C	-2.292681	0.702817	0.370521
C	-1.739662	1.587089	-0.695644
H	-1.040525	0.986726	-1.287183
H	-2.568380	1.817015	-1.377451
C	-1.088281	2.847859	-0.155841
H	-0.677533	3.432145	-0.981151
H	-1.812964	3.468518	0.375922
H	-0.273677	2.606416	0.529944
F	-2.519010	-1.098606	-1.220551
F	-4.323249	-0.221751	-0.415245
F	-3.100892	-1.506211	0.820816
O	-2.271232	0.912008	1.556175
O	0.056093	-1.073700	0.514269

There are no imaginary frequencies

----

trans-P3-OH2

Charge: 0

C	2.810257	-1.850145	0.692068
C	1.850421	-1.648123	-0.478593
C	1.189648	-0.270953	-0.415699

C	2.185156	0.871361	-0.277920
C	3.370545	0.890082	-1.204927
C	3.570955	0.558074	0.255916
C	3.978184	-0.859693	0.647939
H	4.465180	-0.834274	1.626448
H	4.729339	-1.222882	-0.062493
H	4.016960	1.347416	0.852898
H	3.493808	0.078651	-1.914613
H	3.697277	1.859669	-1.565360
C	1.573853	2.213209	0.062350
H	2.345413	2.986932	0.095748
H	1.070634	2.197061	1.032514
H	0.835723	2.504613	-0.693221
H	0.588896	-0.108685	-1.315728
H	2.383465	-1.760059	-1.428483
H	1.057825	-2.401214	-0.468018
H	3.194490	-2.873690	0.692409
H	2.261128	-1.729028	1.633360
H	0.701236	-0.156471	1.489535
C	-2.620707	-0.965426	-0.237358
C	-2.440317	0.430995	0.425156
C	-2.132587	1.564027	-0.495492
H	-1.202975	1.318707	-1.018367
H	-2.912141	1.561591	-1.268630
C	-2.042471	2.905083	0.209960
H	-1.795260	3.685440	-0.511930
H	-2.990501	3.166342	0.685462
H	-1.265374	2.892304	0.977154
F	-1.835027	-1.148304	-1.303013
F	-3.896308	-1.081570	-0.657969
F	-2.391462	-1.956482	0.622481
O	-2.648307	0.508303	1.609224
O	0.232360	-0.241575	0.654202

There are no imaginary frequencies

----

trans-P3-OH5

Charge: 0

C	1.435078	1.442993	-0.879046
C	2.595870	1.890736	0.045624
C	3.187833	0.786568	0.931863
C	3.336791	-0.562191	0.247348
C	3.552824	-0.612437	-1.243850
C	2.202455	-0.965572	-0.657948
C	1.024587	-0.025713	-0.764923
H	0.418122	-0.283266	-1.636743
H	0.573048	0.009516	1.157374
H	1.934067	-2.018066	-0.664594
H	3.654086	0.314169	-1.797336
H	4.146735	-1.432413	-1.632390
C	3.986060	-1.626128	1.100015
H	4.018127	-2.586595	0.578181
H	3.430975	-1.766278	2.033977
H	5.013091	-1.346687	1.359452
H	2.560381	0.640105	1.821689
H	4.163885	1.114943	1.306111
H	3.396485	2.300363	-0.576139
H	2.264911	2.709231	0.689049
H	1.718149	1.616007	-1.920833
H	0.545153	2.050401	-0.701777
C	-2.752956	-1.030586	-0.114014
C	-2.501595	0.381524	0.488607
C	-2.371743	1.504016	-0.486523
H	-1.592673	1.235505	-1.205365
H	-3.307335	1.523341	-1.060774
C	-2.092859	2.841644	0.175534
H	-1.988560	3.615918	-0.586570
H	-2.904584	3.129489	0.847040

H	-1.166512	2.805143	0.754217
F	-2.136582	-1.222890	-1.284111
F	-4.075712	-1.175710	-0.332447
F	-2.377296	-1.999872	0.718554
O	-2.532060	0.479928	1.689100
O	0.133896	-0.254140	0.343568

There are no imaginary frequencies

S8-Int1a-1

Charge: 0

Multiplicity: 2

H	1.42538200	1.47720600	1.06942100
C	-1.09198100	-0.17346400	0.03198800
C	0.37182000	0.30711200	-0.06750800
C	1.35297000	-0.84539400	0.12488500
H	1.16395900	-1.31102100	1.09622100
H	1.13611500	-1.58608700	-0.65080200
C	2.80705600	-0.38829700	0.01592000
H	3.10738200	0.25114800	0.85216900
H	3.46666900	-1.25758900	0.03360400
H	2.98167400	0.15108800	-0.91888500
F	-1.95144600	0.84452200	-0.05337600
F	-1.30915400	-0.79831300	1.19409800
F	-1.37062100	-1.03208300	-0.96015500
O	0.47973800	0.89261300	-1.33965100
O	0.49033900	1.32593300	0.87233300

There are no imaginary frequencies

S8-Int1a-2

Charge: -1

Multiplicity: 1

H	-0.40867700	2.12114000	-0.59976000
C	1.06097400	-0.17470900	-0.00297900
C	-0.39805600	0.34507400	0.13708300
C	-1.35255300	-0.83630900	-0.14716500
H	-1.16402300	-1.22983600	-1.15273800
H	-1.12026600	-1.63485600	0.56735400
C	-2.81231300	-0.42171600	-0.00856800
H	-3.05943300	0.36625300	-0.72582700
H	-3.47896600	-1.26987200	-0.18994500
H	-3.00682500	-0.03831500	0.99610900
F	1.96919600	0.81767800	0.10704700
F	1.32006300	-0.78757000	-1.18512400
F	1.37147200	-1.07993600	0.95410100
O	-0.55152000	0.95617200	1.29515700
O	-0.53556700	1.25131600	-1.00136200

There are no imaginary frequencies

S8-Int1a-3

Charge: 0

Multiplicity: 2

C	0.71414800	0.00661900	-0.39758300
C	-0.02659700	1.30580700	-0.02601100
C	-1.48250400	1.26755300	-0.37745200
C	-2.25383900	0.03094900	-0.17093200
C	-1.48464800	-1.24137600	-0.48289500
C	-0.08637300	-1.19217000	0.13574000
H	-2.02829300	2.20766400	-0.42221400
H	0.08333500	1.47431700	1.06238700
H	0.43869200	2.16908100	-0.51177300

H	0.70525400	-0.05872900	-1.49875200
H	-2.04111100	-2.11118100	-0.11522400
H	-1.39531400	-1.35157500	-1.57275100
H	-0.18678700	-1.10625000	1.22710400
H	0.43804200	-2.13210100	-0.06521000
C	-3.21878800	-0.01021500	1.01374700
C	-3.74039400	0.04518000	-0.39442100
H	-3.30481800	0.88395100	1.62252400
H	-3.29842800	-0.94862100	1.55534300
H	-4.18981200	0.97452500	-0.73165500
H	-4.18813600	-0.85523800	-0.80471900
C	2.21208400	-0.00661600	0.02379700
C	2.38316600	-0.20788100	1.53635200
H	3.44184100	-0.12031200	1.80527500
H	1.83109100	0.54340900	2.11145700
H	2.04114500	-1.19855100	1.85208700
C	2.93734600	-1.14517500	-0.71036100
H	2.87271000	-1.01066100	-1.79627900
H	3.99759000	-1.15770500	-0.43431200
H	2.52120300	-2.12627200	-0.46316200
C	2.88871400	1.31231500	-0.37895100
H	3.97212800	1.23596600	-0.23612400
H	2.70201100	1.54471500	-1.43444400
H	2.53376400	2.15362500	0.22425500

There are no imaginary frequencies

S8-Int1a-4

Charge: 1

Multiplicity: 1

C	-0.65697100	0.08792200	-0.39233500
C	0.12708700	-1.21803700	-0.18496600
C	1.60918500	-1.06765400	-0.39617800

C	2.24025500	0.18150300	-0.48764100
C	1.46412300	1.45689800	-0.31629600
C	0.08526600	1.23411600	0.30552900
H	2.15126500	-1.94779100	-0.73080300
H	-0.03194000	-1.62732300	0.82141800
H	-0.20564400	-1.99198500	-0.88140100
H	-0.64462900	0.29656100	-1.47364000
H	2.06042300	2.17343600	0.25525900
H	1.36728700	1.86496500	-1.33059900
H	0.19898600	1.00867600	1.37359000
H	-0.47642600	2.16872100	0.23964500
C	2.79118200	-0.54803800	0.90925400
C	3.66930400	0.09689200	-0.17197800
H	3.05162300	-1.57467600	1.14031800
H	2.42097400	0.03584400	1.74447900
H	4.27765000	-0.62869500	-0.70147300
H	4.15428600	1.03626400	0.06703600
C	-2.15262000	-0.03231600	0.01259000
C	-2.32910700	-0.06705200	1.53694100
H	-2.03483700	0.88024700	1.99952600
H	-3.38200800	-0.24194400	1.78157200
H	-1.74630600	-0.87019600	2.00111000
C	-2.92656800	1.16846300	-0.55184300
H	-2.84324400	1.20844400	-1.64361700
H	-3.98785100	1.08109800	-0.29691900
H	-2.56964200	2.11942800	-0.14584500
C	-2.75507700	-1.30909300	-0.59297400
H	-3.83934000	-1.31588400	-0.44216500
H	-2.56387200	-1.36274300	-1.67136900
H	-2.35310100	-2.21407100	-0.12673300

There are no imaginary frequencies

S8-Int1a-5

Charge: 1

Multiplicity: 1

C	-0.66103300	0.09419000	-0.40127700
C	0.12830300	-1.21123600	-0.22271300
C	1.60540800	-1.05989700	-0.41459900
C	2.23769900	0.18181300	-0.48825800
C	1.46312500	1.45420700	-0.33339900
C	0.08817300	1.23695100	0.29155300
H	2.15444200	-1.94309800	-0.72232000
H	-0.02940800	-1.64377100	0.77175900
H	-0.20040700	-1.97055600	-0.93499600
H	-0.66586200	0.31480500	-1.47760800
H	2.05765500	2.17316800	0.23391700
H	1.36549500	1.85252200	-1.35030000
H	0.21410000	1.00771500	1.35532900
H	-0.47173000	2.17077000	0.22832500
C	2.80089200	-0.54880500	0.93080400
C	3.66625200	0.09078900	-0.14777800
H	3.02346600	-1.58377200	1.15579400
H	2.41514500	0.04455600	1.75011300
H	4.27433000	-0.62952600	-0.68304400
H	4.14623400	1.03303700	0.08491700
C	-2.15195900	-0.03333200	0.02049100
C	-2.31043900	-0.12516200	1.54348100
H	-1.99266200	0.79621300	2.03923800
H	-3.36203700	-0.29139900	1.79768400
H	-1.73480500	-0.95445300	1.96600900
C	-2.92712300	1.19046400	-0.48792600
H	-2.84565900	1.28008100	-1.57626800
H	-3.98804600	1.09471000	-0.23666300
H	-2.56766400	2.12170300	-0.04237600
C	-2.76978800	-1.28282500	-0.62327600
H	-3.85046700	-1.29655600	-0.45170700
H	-2.60031500	-1.29339600	-1.70544000

H	-2.35885900	-2.20570000	-0.20497500

There are no imaginary frequencies

S8a

Charge: 0

Multiplicity: 1

C	0.07129900	0.00152200	-0.32564400
C	-0.66724200	1.25374300	0.17349300
C	-2.13935100	1.25559200	-0.24506400
C	-2.86302500	-0.00173300	0.23203300
C	-2.13723200	-1.25636000	-0.24912000
C	-0.66733400	-1.24976600	0.17572300
H	-2.63513000	2.15109500	0.14340800
H	-0.60940600	1.29851100	1.26866900
H	-0.18785500	2.15928800	-0.20637800
H	-0.00814400	0.00254800	-1.42416800
H	-2.63197200	-2.15393200	0.13586500
H	-2.19486600	-1.30775100	-1.34430900
H	-0.61806300	-1.28486700	1.27147000
H	-0.18254700	-2.15770400	-0.19193200
H	-2.19970100	1.31032600	-1.33997800
C	1.59829900	-0.00067100	-0.01689700
C	1.88676200	-0.06814600	1.48875000
H	2.96727100	-0.03510600	1.66431500
H	1.43946600	0.77361900	2.02544500
H	1.50936400	-0.99343200	1.93318000
C	2.26286200	-1.20523400	-0.70079900
H	2.04826100	-1.21361000	-1.77513900
H	3.34961500	-1.15882200	-0.57620800
H	1.92663500	-2.15657100	-0.28101400
C	2.24765900	1.27035100	-0.58539900
H	3.33780600	1.20303200	-0.51024700

H	1.99140300	1.40465500	-1.64219800
H	1.93803000	2.16852600	-0.04514800
H	-2.89587000	-0.00400000	1.32945900
H	-3.90047400	-0.00159300	-0.11754800

There are no imaginary frequencies

S8a-Int1

Charge: 1

Multiplicity: 1

C	0.05590100	-0.04586600	-0.37552000
C	-0.65417600	1.27621500	-0.06558900
C	-2.08895300	1.25926000	-0.01305800
C	-2.88539900	0.06990700	0.05036300
C	-2.16882700	-1.23711800	-0.25528600
C	-0.72969200	-1.20311300	0.25508800
H	-2.59890700	2.21711300	0.07396200
H	-0.43612000	1.65148000	0.96243100
H	-0.34391200	2.13564900	-0.67290900
H	0.00723000	-0.17004700	-1.46480100
H	-2.72155700	-2.06132500	0.19795000
H	-2.17739100	-1.39718800	-1.33737000
H	-0.73829000	-1.09487500	1.34570700
H	-0.24716000	-2.15486800	0.02935300
C	1.56724500	-0.01275300	-0.01137700
C	1.79438700	-0.00228600	1.50527300
H	1.44583500	-0.92670300	1.97326500
H	2.86365900	0.09386900	1.71773600
H	1.28874900	0.83729400	1.99345900
C	2.25274000	-1.24896300	-0.60980100
H	2.10587800	-1.29103400	-1.69408700
H	3.32911200	-1.21049100	-0.41658400
H	1.87474200	-2.17919200	-0.17827200

C	2.22048900	1.23446700	-0.62472000
H	3.30657400	1.18500800	-0.50265200
H	2.00449400	1.30677300	-1.69610000
H	1.88011900	2.15604700	-0.14433800
H	-3.17666700	0.13665400	1.12817800
H	-3.84868700	0.22733500	-0.44716300

There are no imaginary frequencies

S5.3 Supplementary Figures of Computational Results

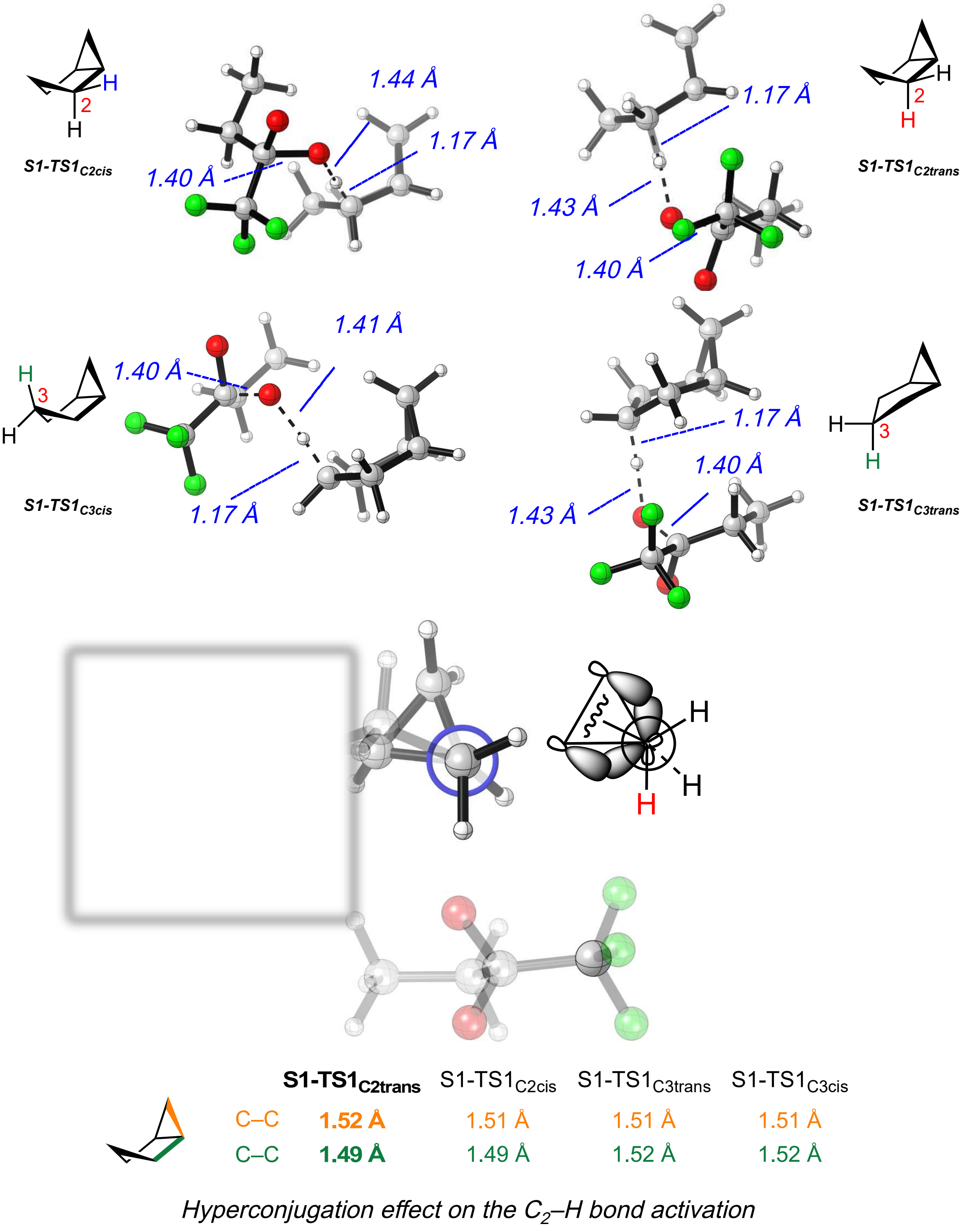

Figure S7. Transition state structures and structural analysis in C–H bond activation of bicyclo[3.1.0]hexane (**S1**).

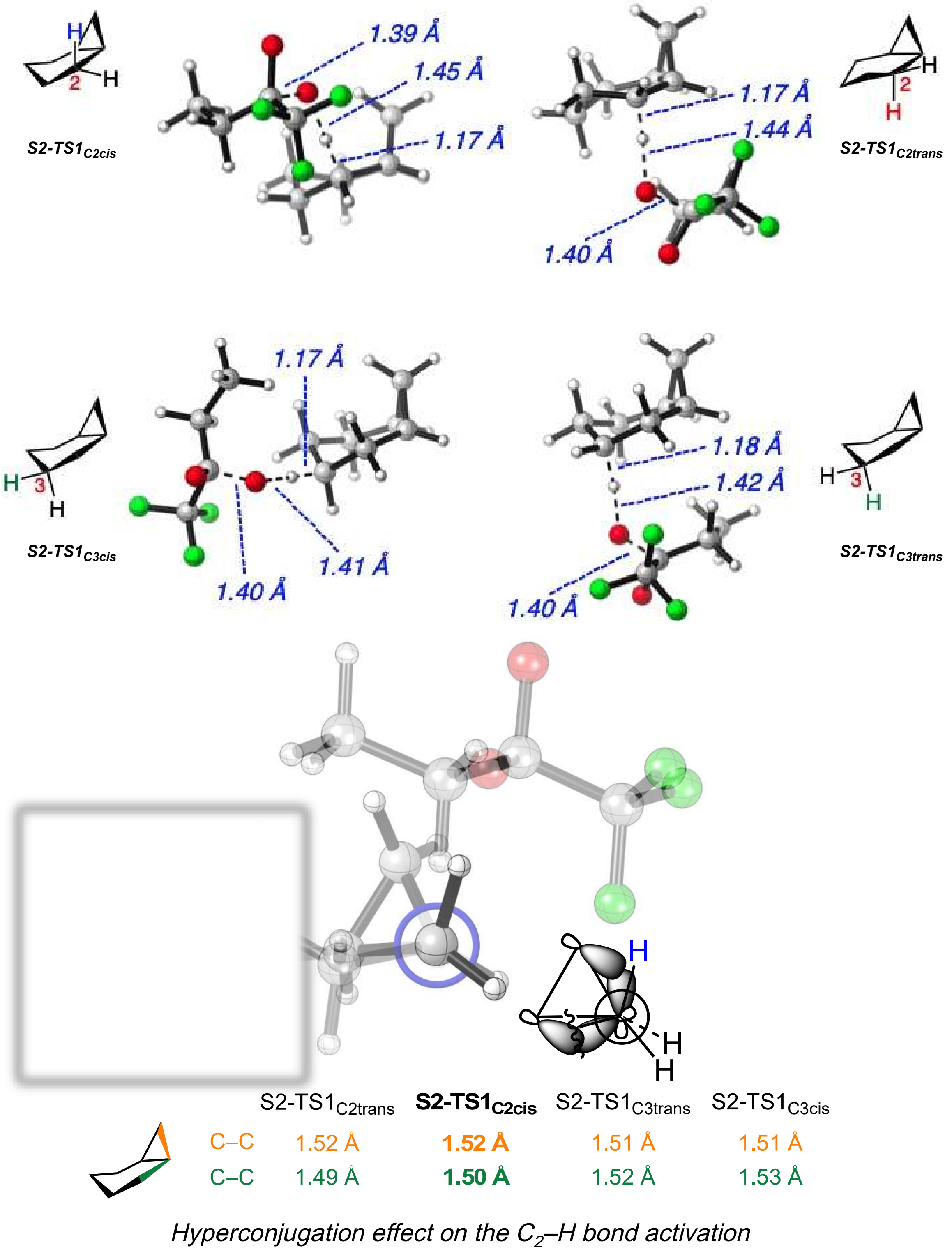

Figure S8. Transition state structures and structural analysis in C–H bond activation of bicyclo[4.1.0]heptane (**S2**).

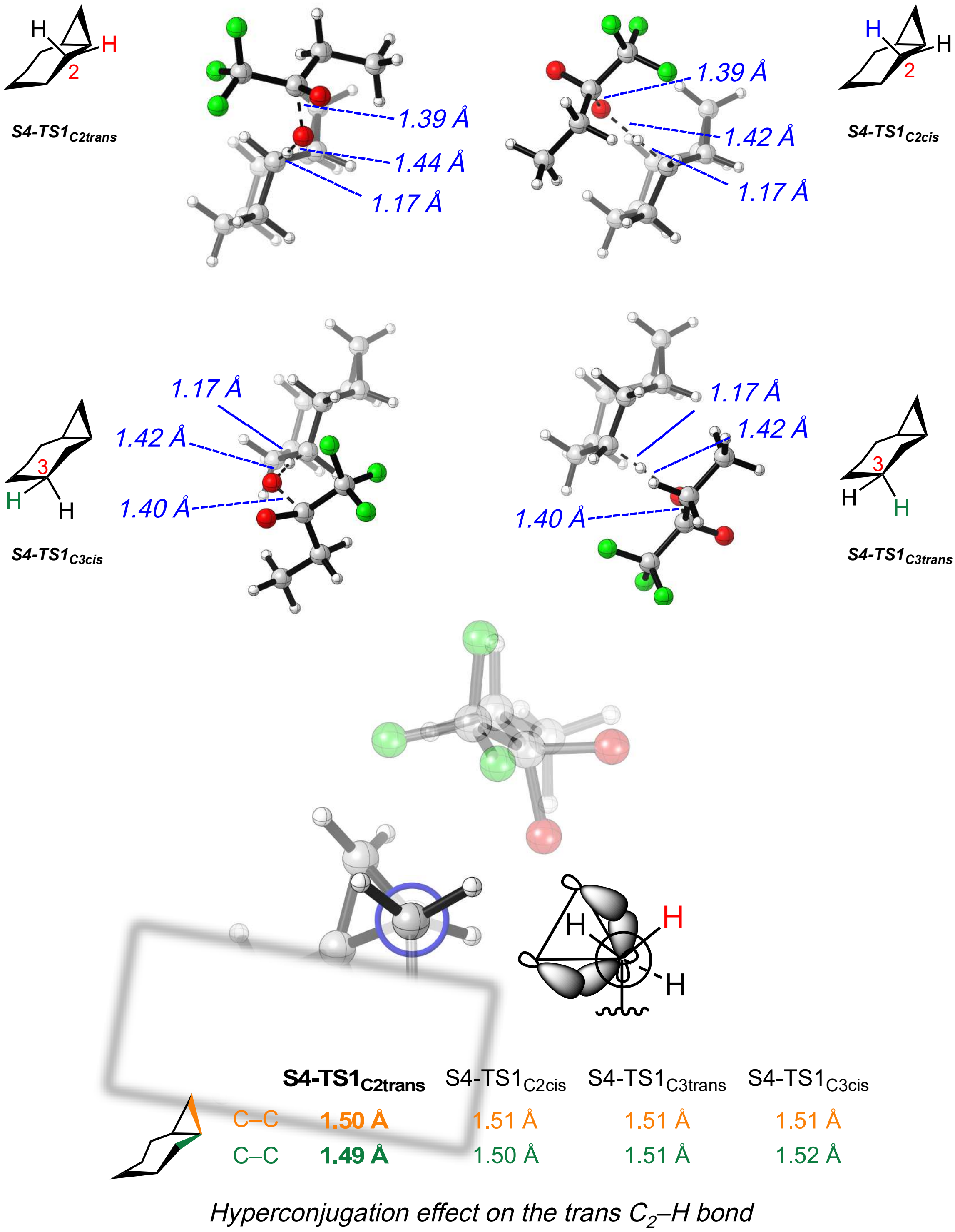

Figure S9. Transition state structures and structural analysis in C–H bond activation of bicyclo[5.1.0]octane (**S4**).

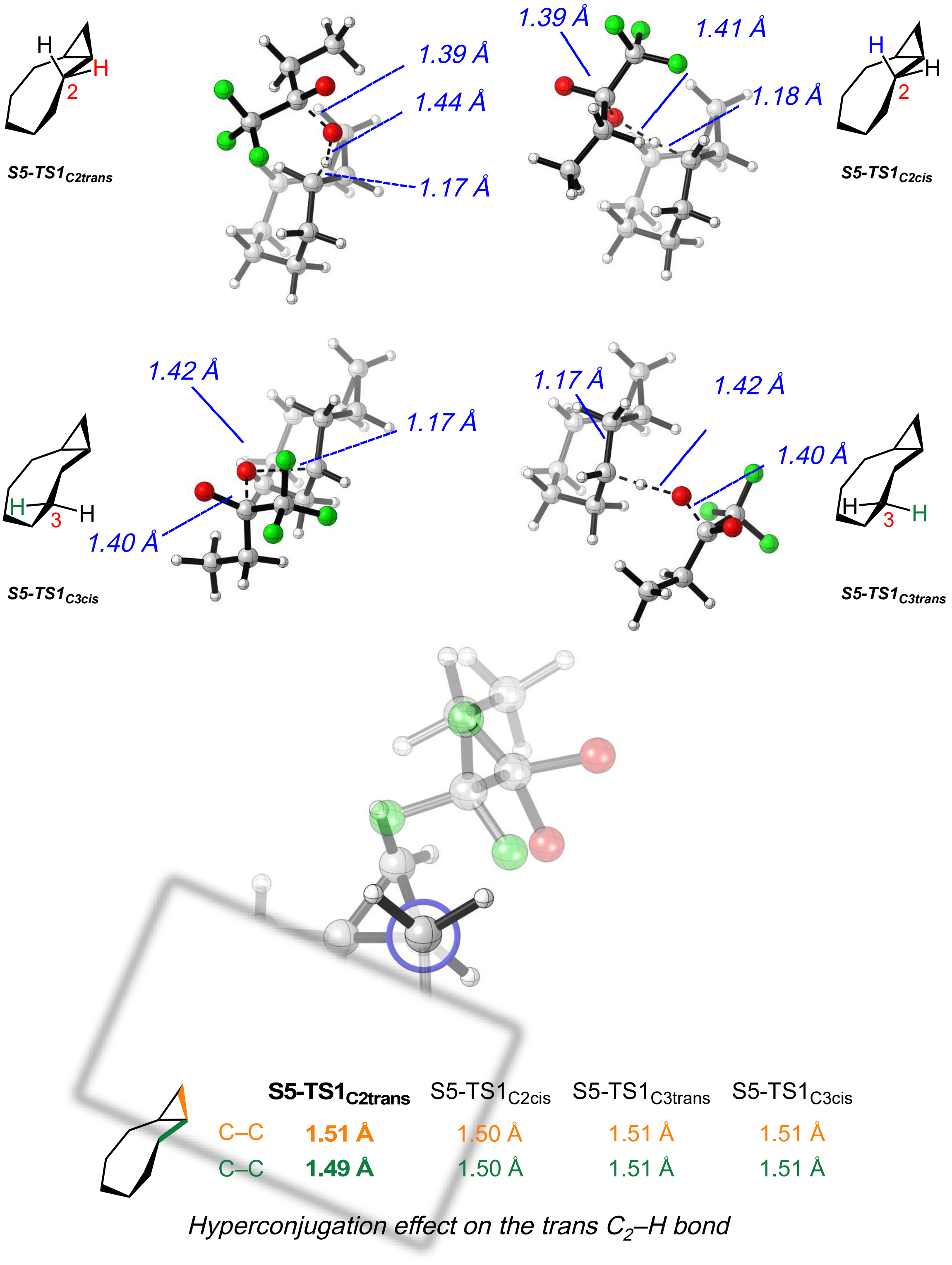

Figure S10. Transition state structures and structural analysis in C–H bond activation of bicyclo[6.1.0]nonane (**S5**).

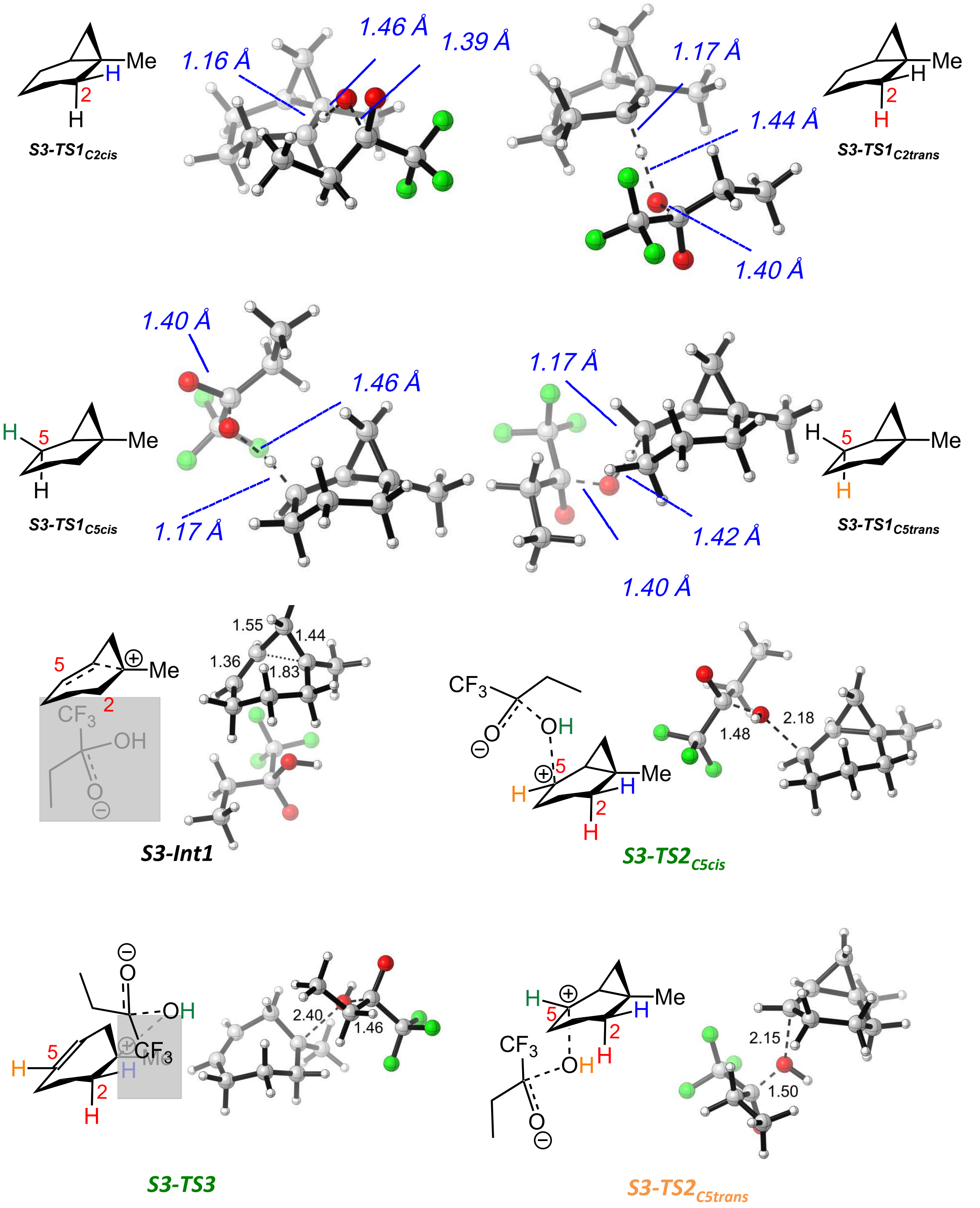

Figure S11. Transition state and intermediate structures in the oxygenation with 1-methylbicyclo[4.1.0]heptane (**S3**).

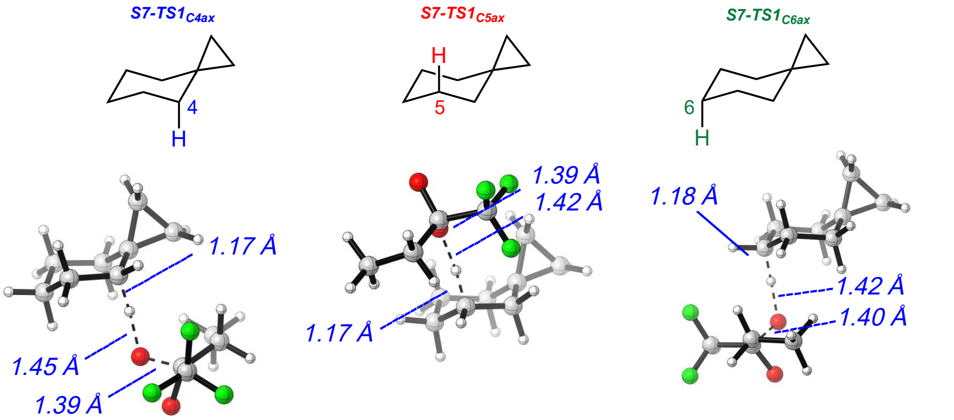

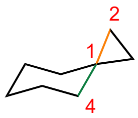

	**S7-TS1$_{C4ax}$**	S7-TS1$_{C5ax}$	S7-TS1$_{C6ax}$
C_1–C_2	**1.52 Å**	1.51 Å	1.51 Å
C_1–C_4	**1.49 Å**	1.52 Å	1.52 Å

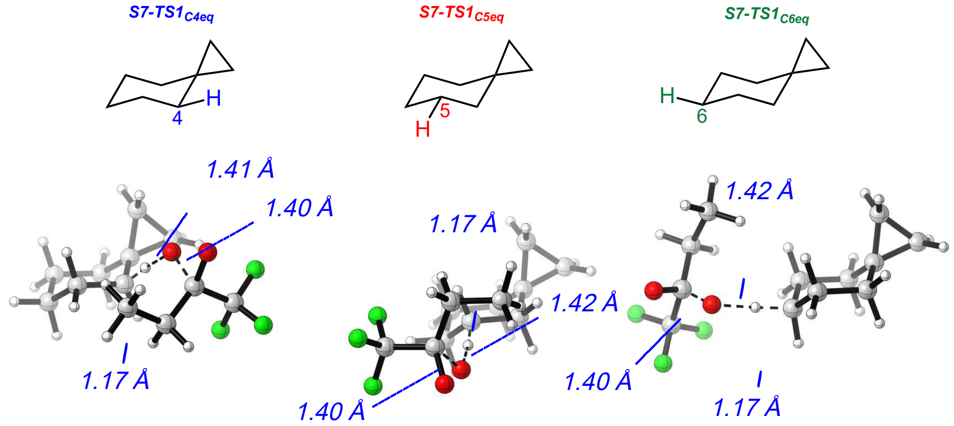

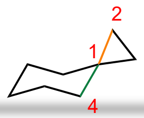

	S7-TS1$_{C4eq}$	S7-TS1$_{C5eq}$	S7-TS1$_{C6eq}$
C_1–C_2	1.50 Å	1.51 Å	1.51 Å
C_1–C_4	1.50 Å	1.52 Å	1.51 Å

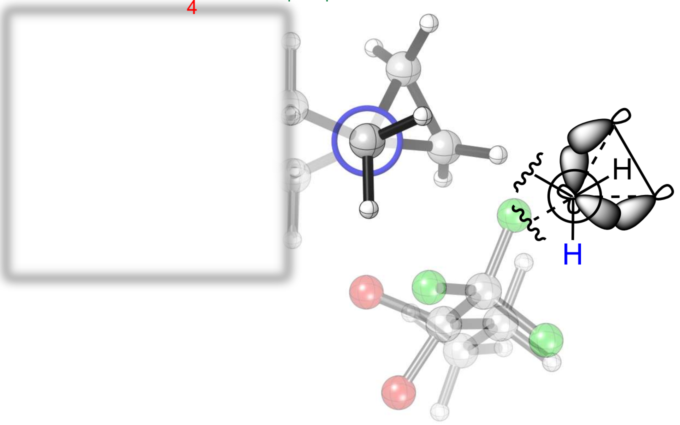

Hyperconjugation effect on the axial C–H bond activation

Figure S12. Transition state and intermediate structures in the oxygenation with spiro[2.5]octanes (**S7**).

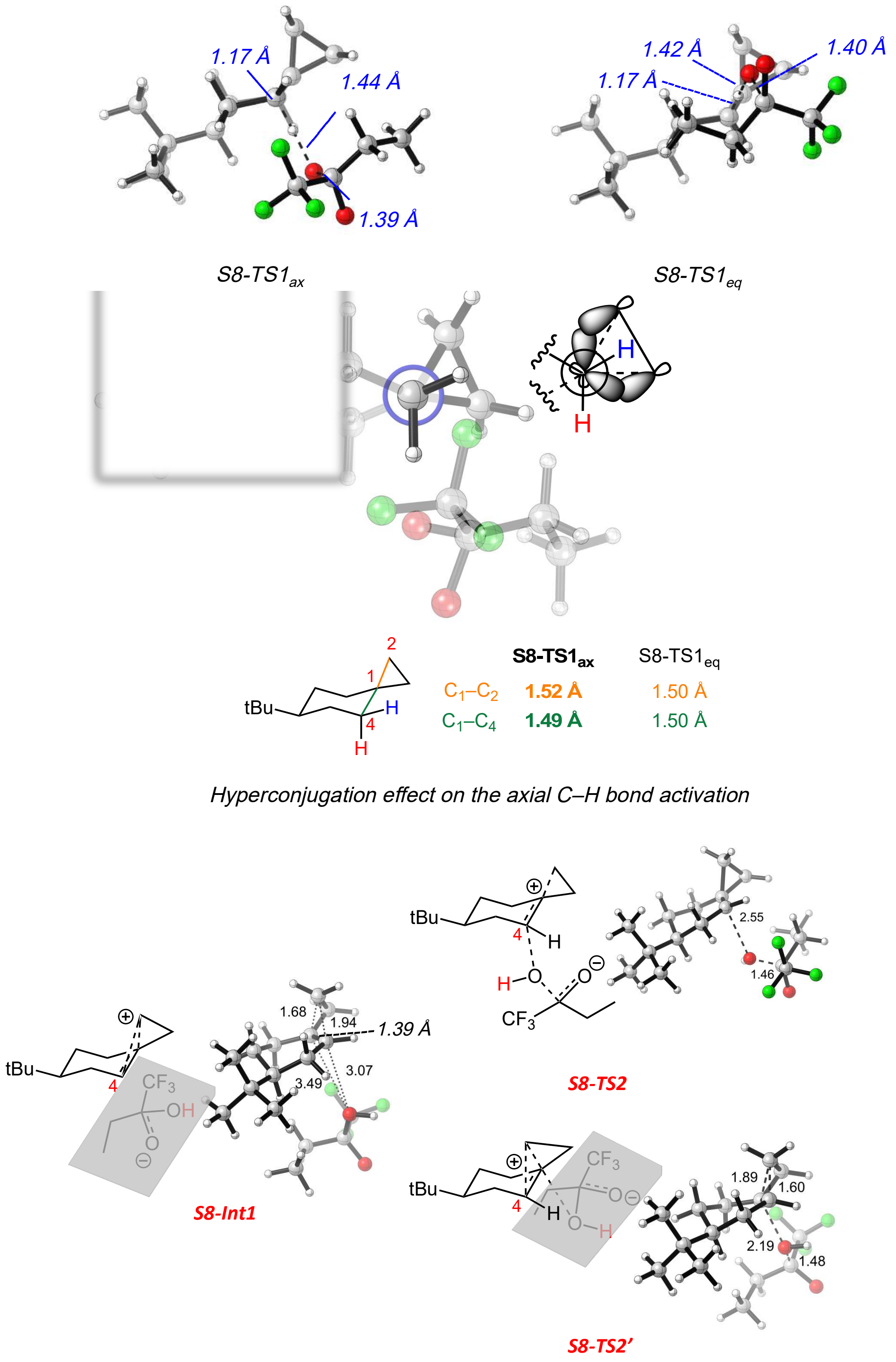

Figure S13. Transition state and intermediate structures in the oxygenation with 6-(tert-butyl)spiro[2.5]octanes (**S8**).

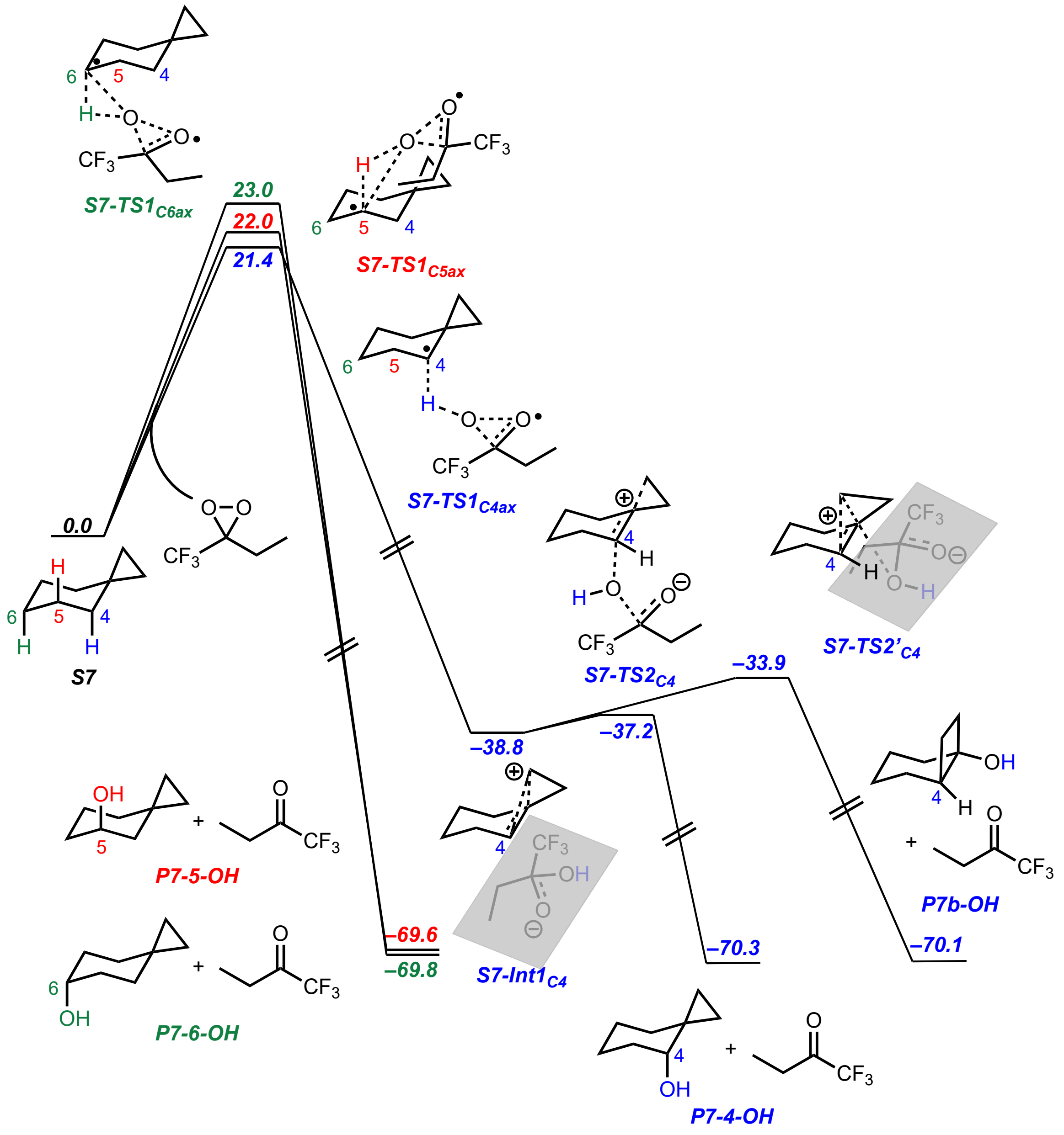

Figure S14. Energy profile in the oxygenation with spiro[2.5]octanes **S7**.

(u)M062X/6-31+g(d,p), CPCM(acetonitrile)

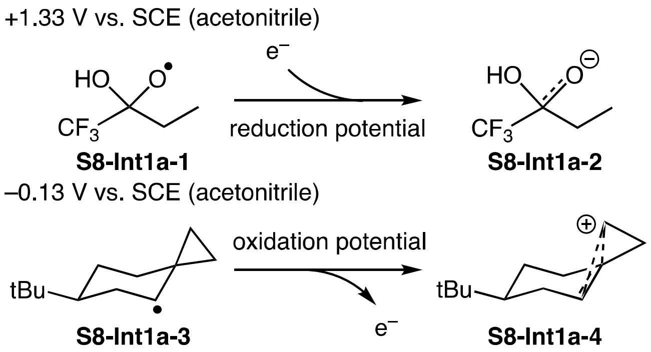

Figure S15. In silico redox potentials of 1,1,1-trifluoro-2-hydroxybutoxy radical **S8-Int1a-1** and 6-(*tert*-butyl)spiro[2.5]octan-4-yl radical **S8-Int1a-3**.

Nicewicz et al. reported a computational method for determining in silico redox potentials.[28] Following their procedures, we calculated the redox potential in the formation of 1,1,1-trifluoro-2-hydroxybutan-2-olate and 6-(*tert*-butyl)spiro[2.5]octan-4-ylium from the hypothetical radical pair S8-Int1a. We found that reduction potential of 1,1,1-trifluoro-2-hydroxybutoxy radical is +1.33 V *vs.* SCE (acetonitrile), and oxidation potential from 6-(*tert*-butyl)spiro[2.5]octan-4-yl radical intermediate to 6-(*tert*-butyl)spiro[2.5]octan-4-ylium cation is –0.13 V *vs.* SCE (acetonitrile). The in silico redox potentials suggest formation of the charged species via an exergonic redox process.

S6. References

1. Galeotti, M.; Vicens, L.; Salamone, M.; Costas, M.; Bietti, M. *J. Am. Chem. Soc.* **2022**, *144*, 7391-7401

2. Jackson, J. E.; Misalitz, U.; Jones, M.; De Meijere, A. *Tetrahedron*. **1987**, *43*, 653-662.

3. Friedrich, E. C.; Lunetta, S. E.; Lewis, E. J. *J. Org. Chem.* **1989**, *54*, 2388-2390.

4. O'Connor, E. J.; Brandt, S. Helquist, P. *J. Am. Chem. Soc.* **1987**, *109*, 3739-3747.

5. Tchawou, A. A. S. W.; Raducan, M.; Chen, P. *Organometallics* **2017**, *36*, 180-191.

6. Martin, T.; Galeotti, M.; Salamone, M.; Liu, F.; Yu, Y.; Duan, M.; Houk, K. N.; Bietti, M. *J. Org. Chem.* **2021**, *86*, 9925-9937.

7. Shuler, W. G.; Johnson, S. L.; Hilinski, M. K. *Org. Lett.* **2017**, *19*, 4790-4793.

8. Uyanik, M.; Akakura, M.; Ishihara, K. *J. Am. Chem. Soc.* **2009**, *131*, 251-262.

9. Alorati, A. D.; Bio, M. M.; Brands, K. M. L.; Cleator, E.; Davies, A. J.; Wilson, R. D.; Wise, C. S. *Org. Process Res. Dev.* **2007**, 11, 637-641.

10. Friedrich, E. C.; Biresaw, G. *J. Org. Chem.* **1982**, *47*, 1615-1618.

11. De Costa, B. R.; George, C.; Burke, T. R.; Rafferty, M. F.; Contreras, P. C.; Mick, S. J.; Jacobson, A. E.; Rice, K. C. *J. Med. Chem.* **1988**, *31*, 1571-1575.

12. Denmark, S. E.; Edwards, J. P. *J. Org. Chem.* **1991**, *56*, 6974-6981.

13. Rodriguez-Garcia, C.; Ibarzo, J.; Alvarez-Larena, A.; Branchadell, V.; Oliva, A.; Ortuno, R. M. *Tetrahedron* **2001**, *57*, 1025-1034.

14. Newcomb, M.; Shen, R.; Lu, Y.; Coon, M. J.; Hollenberg, P. F.; Kopp, D. A.; Lippard, S. J. *J. Am. Chem. Soc.* **2002**, *124*, 6879-6886.

15. Newcomb, M.; Dharmika, R. E. P.; Lansakara, D. S. P.; Kim, H.-Y.; Lippard, S. J.; Beauvais, L. G.; Murray, L. J.; Izzo, V.; Hollemberg, P. F.; Coon, M. J. *J. Org. Chem.* **2007**, *72*, 1121-1127.

16. Kirschberg, T.; Mattay, J. *J. Org. Chem.* **1996**, *61*, 8885-8896.

17. Mash, E. A.; Nelson, K. A. *Tetrahedron* **1987**, *43*, 679-692.

18. Gassman, P. G.; Atkins, T. J. *J. Am. Chem. Soc.* **1972**, *94*, 7741-7748.

19. Li, P.-F.; Yi, C.-B.; Qu, J. *Org. Biomol. Chem.* **2015**, *13*, 5012.

20. Mash, E. A.; Gregg, T. M.; Kaczynski, M. A. *J. Org. Chem.* **1996**, *61*, 2743-2752.

21. (a) Cope, A. C.; Moon, S.; Park, C. H. *J. Am. Chem. Soc.* **1964**, *84*, 4843-4849. (b) Hanold, N.; Meier, H. *Chem. Ber.* **1985**, *118*, 198-209.

22. Poulter, C. D.; Friedrich, E. C.; Winstein, S. *J. Am. Chem. Soc.* **1970**, *92* ,4274-4281.

23. Herbert, M.; Antony-Meyer, C.; Schulz-Popitz, C.; Zerban, G. *Liebigs Ann. Chem.* **1987**, 1087-1094.

24. Chen, M. S.; White, M. C. *Science* **2010**, *327*, 783-787.

25. (a) Hanack, v. M.; Schneider, H.-J.; *Liebigs Ann. Chem.* **1965**, *686*, 8-18. (b) Wiberg, K. B.; Hiatt, J. E.; Hseih, K. *J. Am. Chem. Soc.* **1970**, *92*, 544-553.

26. Lillie, T. S.; Ronald, R. C. *J. Org. Chem.* **1985**, *50*, 5084-5088.

27. Liu, F.; Ma, S.; Lu, Z.; Nangia, A.; Duan, M.; Yu, Y.; Xu, G.; Mei, Y.; Bietti, M.; Houk, K. N. *J. Am. Chem. Soc.* **2022**, 144, 6802–6812.

28. Roth, H. G.; Romero, N. A.; Nicewicz, D. A. *Synlett* **2016**, 27, 714–723.
